# Safety and immunogenicity of a typhoid conjugate vaccine among children aged 9 months to 12 years in Malawi: a nested substudy of a double-blind, randomised controlled trial

**DOI:** 10.1016/S2214-109X(22)00275-3

**Published:** 2022-08-09

**Authors:** Nginache Nampota-Nkomba, Osward M Nyirenda, Lameck Khonde, Victoria Mapemba, Maurice Mbewe, John M Ndaferankhande, Harrison Msuku, Clemens Masesa, Theresa Misiri, Felistas Mwakiseghile, Priyanka D Patel, Pratiksha Patel, Ifayet Johnson-Mayo, Marcela F Pasetti, Robert S Heyderman, J Kathleen Tracy, Shrimati Datta, Yuanyuan Liang, Kathleen M Neuzil, Melita A Gordon, Matthew B Laurens, Typhoid Vaccine Acceleration Consortium team

**Affiliations:** a Blantyre Malaria Project, Kamuzu University of Health Sciences, Blantyre, Malawi; b Malawi-Liverpool-Wellcome Trust Clinical Research Programme, Kamuzu University of Health Sciences, Blantyre, Malawi; c Center for Vaccine Development and Global Health, University of Maryland School of Medicine, Baltimore, MD, USA; d Department of Infection, Division of Infectious Diseases, University College London, London, UK; e University of Liverpool, Liverpool, UK

## Abstract

**Background:**

Typhoid fever is a substantial public health problem in Africa, yet there are few clinical trials of typhoid conjugate vaccine (TCV). We assessed immunogenicity and safety of Typbar TCV in Malawi.

**Methods:**

This substudy was nested within a phase 3, double-blind, parallel design, randomised controlled trial of TCV in children from Ndirande Health Centre in Ndirande township, Blantyre, Malawi. To be eligible, participants had to be aged between 9 months and 12 years with no known immunosuppression or chronic health conditions, including HIV or severe malnutrition; eligible participants were enrolled into three strata of approximately 200 children (9–11 months, 1–5 years, and 6–12 years), randomly assigned (1:1) to receive TCV or control (meningococcal serogroup A conjugate vaccine [MCV-A]) intramuscularly. Serum was collected before vaccination and at 28 days and 730–1035 days after vaccination to measure anti-Vi antibodies by ELISA. Because of COVID-19, day 730 visits were extended up to 1035 days. This nested substudy evaluated reactogenicity, safety, and immunogenicity by age stratum. Safety outcomes, analysed in the intention-to-treat population, included solicited adverse events within 7 days of vaccination (assessed on 3 separate days) and unsolicited adverse events within 28 days of vaccination. This trial is registered with ClinicalTrials.gov, NCT03299426.

**Findings:**

Between Feb 22 and Sept 6, 2018, 664 participants were screened, and 631 participants were enrolled and randomly assigned (320 to the TCV group and 311 to the MCV-A group). 305 participants in the TCV group and 297 participants in the MCV-A group were vaccinated. Among TCV recipients, anti-Vi IgG geometric mean titres increased more than 500 times from 4·2 ELISA units (EU)/mL (95% CI 4·0–4·4) at baseline to 2383·7 EU/mL (2087·2–2722·3) at day 28, then decreased to 48·0 EU/mL (39·9–57·8) at day 730–1035, remaining more than 11 times higher than baseline. Among MCV-A recipients, anti-Vi IgG titres remained unchanged: 4·3 EU/mL (4·0–4·5) at baseline, 4·4 EU/mL (4·0–4·7) on day 28, and 4·6 EU/mL (4·2–5·0) on day 730–1035. TCV and MCV-A recipients had similar solicited local (eight [3%] of 304, 95% CI 1·3–5·1 and three [1%] of 293, 0·4–3·0) and systemic (27 [9%] of 304, 6·2–12·6 and 27 [9%] of 293, 6·4–13·1) reactogenicity. Related unsolicited adverse events occurred similarly in TCV and MCV-A recipients in eight (3%) of 304 (1·3–5·1) and eight (3%) of 293 (1·4–5·3).

**Interpretation:**

This study provides evidence of TCV safety, tolerability, and immunogenicity up to 730–1035 days in Malawian children aged 9 months to 12 years.

**Funding:**

Bill & Melinda Gates Foundation.

## Introduction

Typhoid fever is an acute enteric disease caused by *Salmonella enterica* serovar Typhi (*S* Typhi). *S* Typhi is a rod-shaped, gram-negative, anaerobic bacterium, which expresses a surface polysaccharide capsule (Vi antigen), an important determinant of virulence. *S* Typhi is transmitted by the faecal-oral route through ingestion of contaminated food and water, often resulting from inadequate hygiene and sanitation.^1^

The non-specific symptoms of typhoid fever make it difficult to distinguish from other febrile illnesses including malaria, dengue fever, and influenza.^2^ This characteristic, together with an absence of sensitive, affordable, and readily available diagnostic testing for laboratory confirmation of *S* Typhi in many low-income and middle-income countries, often leads to a delay in diagnosis.^3^ If left untreated, serious complications including intestinal perforation, haemorrhage, and neurological complications, such as meningitis, neuropsychiatric conditions, and encephalopathy, can arise.^4^, ^5^ Even after successful treatment, up to 5% of people can have gall bladder colonisation, which results in prolonged shedding and transmission.^6^ Although typhoid fever is treatable with antibiotics, multidrug-resistant strains are present in sub-Saharan Africa and extensively drug-resistant strains in Asia.^7^, ^8^

**Research in context**

**Evidence before this study**

In 2017, WHO recommended typhoid conjugate vaccine (TCV) be introduced in typhoid-endemic countries and prioritised in countries with the highest disease burden or prevalence of antimicrobial resistant *Salmonella enterica* serovar Typhi. This recommendation was informed by immunogenicity data, including data on measles coadministration in Asian children and efficacy data from an adult human challenge study in the UK. At the time of the recommendation, there were no data from African populations on immunogenicity, or on coadministration of any TCV with routine vaccines given at 9 months of age.

We did a PubMed search using the terms “typhoid”, “conjugate”, and “vaccine” between Jan 1, 1980, and July 5, 2021, with no language restrictions, and looked for clinical trials among children from 6 months to 15 years. The first TCV evaluated in children (Vi-polysaccharide conjugated to *Pseudomonas aeruginosa* exotoxin A) showed high efficacy and strong immunogenicity in Vietnamese children aged 2–5 years but did not advance in clinical development. Other TCVs assessed in children include Vi polysaccharide conjugated to diphtheria toxoid (Vi-DT), CRM_197_ protein (Vi-CRM_197_), and tetanus toxoid (Vi-TT). The most advanced Vi-DT vaccine candidates are in phase 3 trials in children and adults in Indonesia and Nepal. Three Vi-TT vaccines and one Vi-CRM_197_ vaccine have been licensed in India on the basis of immunogenicity and efficacy data in Indian children as young as 6 months. Before the start of this study (February, 2018), no immunogenicity, safety, or efficacy data were available from the African continent, for any TCV.

**Added value of this study**

Individual response to vaccines can vary on the basis of demographic, environmental, and genetic factors. As a result, data from participants in various parts of the world are essential. This is the first study to assess immunogenicity of a TCV in African children as young as 9 months of age, including coadministration with measles–rubella vaccine given as part of routine immunisation. Our results show that Typbar TCV is safe and highly immunogenic in Malawian children 9 months to 12 years of age and does not interfere with measles–rubella vaccine when coadministered at 9 months. These immunogenicity data can be used to support TCV introduction in typhoid-endemic countries without data, which is important particularly in sub-Saharan Africa, where young children carry a high burden of typhoid fever.

**Implications of all the available evidence**

Our study provides data that can help to support TCV introduction in Africa. These data will provide a framework for comparative immunogenicity studies with other typhoid vaccines.

Annually, there are more than 9 million cases of typhoid fever and more than 110 000 deaths worldwide, mostly among children and young adults in Asia and sub-Saharan Africa.^9^ Children younger than 15 years are disproportionately affected, with a substantial burden in infants and children younger than 2 years of age.^10^ In Malawi, the incidence of typhoid fever is highest among children aged 5–9 years at 861 cases per 100 000 person-years of observation, followed by children under 5 years at 632 person-years of observation.^11^ Despite broad improvements in prevention and control, such as advancement in water and sanitation infrastructure, improved food handling, and increased access to antibiotics, annual disease burden remains high.^12^

Although whole-cell and polysaccharide typhoid vaccines have been available since the 1980s, uptake in endemic regions has been hindered by moderate and short-lived protection, no regulatory approval for children younger than 2 years of age, and the absence of funding from Gavi, the Vaccine Alliance.^13^ The typhoid conjugate vaccine (TCV), Typbar TCV (Bharat Biotech International, Hyderabad, India), which contains the Vi-antigen polysaccharide conjugated to a tetanus-toxoid protein carrier reached WHO prequalification in 2017.^14^ In 2018, WHO recommended a single-dose TCV for control of typhoid fever beginning as early as 6 months of age in typhoid-endemic regions, with priority in countries that have the highest burden of typhoid disease or a high burden of antimicrobial-resistant *S* Typhi.^15^ This recommendation was made on the basis of safety and immunogenicity data from a study in Asian children and efficacy data from an adult human challenge study in the UK. At the time this trial was done, there were no data from Africa on TCV efficacy and immunogenicity, including coadministration of TCV with routine vaccines given at 9 months of age.^16^

Human vaccine response might differ on the basis of regional demographic, environmental, and genetic factors, and coadministration with other vaccines. We did an immunogenicity, safety, and coadministration substudy nested within a phase 3, double-blind, randomised controlled trial in healthy Malawian children aged 9 months to 12 years. The primary objective of the parent study was to measure TCV efficacy in reducing blood culture-confirmed typhoid fever in children 9 months to 12 years of age.^17^ The primary objective of the substudy was to evaluate the immunogenicity and safety of TCV versus meningococcal serogroup-A conjugate vaccine (MCV-A). We present safety, immunogenicity, and coadministration data from a subset of children enrolled in the first TCV efficacy trial done in Africa.

## Methods

### Study design

This double-blind, randomised controlled, safety and immunogenicity study was done at Ndirande Health Centre in Ndirande township, Blantyre, Malawi. Ethical approval was obtained from the Malawi National Health and Science Review Committee (FWA00005976), the University of Liverpool Ethical Review Board (FWA00005266), and the University of Maryland Institutional Review Board (FWA00007145).

### Participants

Participants were recruited from Ndirande Health Centre, a government-supported primary-health-care facility, and Ndirande township in Blantyre, Malawi. Ndirande is a densely populated peri-urban and urban township in Malawi's commercial centre with approximately 200 000 inhabitants. Community engagement ensured acceptance of local leaders and parent–teacher groups. Recruitment strategies were broad and varied, and included study advertisement via radio, television, and print media. The main trial (target n=28 000) recruited 28 130 participants. Substudy participants (target n=600 with 200 in each stratum) were recruited from three age strata as follows: 9–11 months (n=200), 1–5 years (n=200), and 6–12 years (n=200). For the 9–11-month stratum, we recruited children who had not yet received a measles–rubella-containing vaccine. For this substudy, children who were HIV exposed but uninfected, children with HIV, children with other immune-suppressed states, including tuberculosis, children with severe malnutrition, and children with chronic cardiovascular, pulmonary, renal, and neurological diseases were excluded (full eligibility criteria are listed in appendix p 1). Written informed consent was sought from parents or guardians. Written assent was obtained from children aged 8 years and older. Eligibility criteria were then reviewed, a physical exam was conducted, and anthropometry data collected.

### Randomisation and masking

Eligible participants, within each age stratum, were randomly assigned at a 1:1 ratio to receive either TCV or MCV-A using stratified block randomisation with varying block sizes of six to 12. An unmasked biostatistician generated and maintained the allocation sequence using the blockrand package (version 1.3) in R software, version 3.4.1 (R Foundation for Statistical Computing). The randomisation sequence was populated onto tablets accessible only to unmasked nurses. Unmasked nurses assigned the treatment group, prepared and administered vaccines in a private area inaccessible to masked study staff, and were not involved in study-related assessments after vaccination. Participants, caregivers, and study staff who were involved in follow-up activities were masked to study treatment assignments. Data collection and entry were summarised previously.^18^

### Procedures

The study vaccine, Typbar TCV (Bharat Biotech International, Hyderabad, India) consists of 25 μg of Vi-antigen polysaccharide conjugated to a non-toxic inactivated tetanus toxoid-protein carrier. TCV was administered intramuscularly (day 0) as a 0·5 mL dose. The control vaccine, MCV-A (Serum Institute of India PVT; Hyderabad, India), is a lyophilised vaccine that consists of meningococcal group A polysaccharide conjugated to tetanus toxoid protein in aluminium phosphate adjuvant. We administered 5 μg/0·5 mL to participants 9–11 months and 10 μg/0·5 mL to those aged 1 year or older as an intramuscular dose on day 0. Study or control vaccine was administered in the left thigh in infants and left arm in older children. For children aged 9–11 months, we coadministered measles–rubella vaccine in the right thigh in accordance with the Malawi Expanded Programme on Immunisation. Participants were observed, in accordance with the protocol, by study clinicians for 30 min after vaccination for any adverse events. Participants were followed up in person, through home or clinic visits, for safety assessments on days 0, 3, 7, 28, 180, and 730–1035 (because of COVID-19, day 730 visits were extended up to 1035 days) after vaccination. Immunogenicity blood samples were collected on day 0 (before vaccination), on day 28, and 730–1035 days after vaccination. In-person follow-up was paused from March 25, 2020 because of COVID-19 restrictions. This occurred after all day 0 to day 180 visits and 76 day 730 visits were completed. In-person follow-up resumed on Oct 7, 2020, and as a result, the duration of follow-up for the substudy was increased from 730 days (2 years) after vaccination to up to 1035 days (3 years) after vaccination. The protocol amendment to extend this follow-up window was approved by the Malawi National Health and Science Review Committee, the University of Liverpool Ethical Review Board, and the University of Maryland, Baltimore Institutional Review Board.

### Outcomes

This nested substudy evaluated reactogenicity, safety, and immunogenicity by age stratum. Safety outcomes were measured by comparing the proportions of participants who had predefined safety events among children who received TCV and those who received MCV-A at each visit. We followed up and assessed solicited and unsolicited local and systemic reactions on the evening of vaccination, and on day 3 and day 7 after vaccination. We documented all non-serious unsolicited adverse events for 28 days after vaccination and serious adverse events (SAEs) for 6 months after vaccination. Immediate reactions (within 30 min of vaccination) and SAEs were reported with the main trial efficacy results for the entire trial cohort (n=28 130).^17^ Adverse events were defined as any untoward medical occurrences in participants to whom TCV or MCV-A had been administered, including occurrences that were not necessarily caused by or related to the vaccine. SAEs were any untoward medical occurrences that resulted in death, were life threatening, required inpatient hospitalisation or prolongation of existing hospitalisation, or resulted in persistent or substantial disability.

To assess immunogenicity outcomes, blood specimens were processed, and serum stored on the day of collection. In samples collected at days 0, 28, and 730–1035, we measured and compared among children who received TCV and those who received MCV-A, geometric mean titres (GMTs) of anti-Vi immunoglobulin G (IgG) using VaccZyme Human anti-*S* Typhi Vi ELISA kits (The Binding Site Group, Birmingham, UK). In the participants aged 9–11 months, for samples collected on days 0 and 28, we measured measles antibodies through plaque-reduction neutralisation (PRN) and rubella IgG by ELISA. PRN assays were done as previously described^19^ using the third WHO (97/648) standard (NIBSC, Hertfordshire, UK). The rubella IgG ELISA followed standard procedures^20^, ^21^ with modifications: plates were coated with 0·5 μg/mL of rubella K1S antigen (Microbix Biosystems #EL-05-10) and the anti-rubella immunoglobulin WHO RUBI-1-94 (NIBSC) was used as standard.

### Statistical analysis

The main trial sample size and power calculations have been reported previously, with target enrolment of 28 000 children.^17^ This substudy was a descriptive analysis and sample size was not formally calculated. The sample size chosen was based on the feasibility of conducting detailed follow-up of study participants within the larger study. The substudy participant target enrolment was 600 children with 200 children in each of three age strata as follows: 9–11 months (n=200), 1–5 years (n=200), and 6–12 years (n=200). Participants were randomly assigned at a 1:1 ratio to receive study vaccine (TCV group, n=300; 9–11 months, n=100; 1–5 years, n=100; and 6–12 years, n=100) or control vaccine (MCV-A group, n=300; 9–11 months, n=100; 1–5 years, n=100; and 6–12 years, n=100).

Safety outcomes were analysed on the basis of an intention-to-treat (ITT) analysis that included all children who underwent randomisation and received a dose of a vaccine. All vaccinated participants with any safety data after vaccination were included in the safety analyses. To assess safety, we calculated the number of participants, and proportion in each vaccine group, with solicited local and systemic reactions for each visit and unsolicited adverse events. Immunogenicity outcomes were analysed both in the per-protocol population, which included only participants whose samples were collected within the allowable window for their scheduled visit, and the ITT population. Log_10_-transformed Vi antigen, measles, and rubella titres were compared between vaccine groups using a two-sample t test. The distribution of each antibody measure of interest at each time point in each vaccine group was examined graphically and described in terms of sample size, geometric mean, and corresponding 95% CIs. When computing geometric means, zeros or those values that were lower than the limit of detection were replaced by one half the limit of detection (ie, 3·7 EU/mL for Vi antigen, 0·85 mIU/mL for measles, and 0·05 IU/mL for rubella). Proportions of seroconversion for Vi antigen, and of seroprotection for measles and rubella, were compared using Wilson CIs. Seroconversion was defined as a four-fold rise or more in antibody titre from day 0 to day 28 and from day 0 to day 730–1035 after vaccination. Seroprotection was defined in accordance with the WHO standard reference values of GMT of 120 mIU/mL for measles or higher and 10 IU/mL or higher for rubella.^22^, ^23^ Study results were analysed using SAS software, version 9.4.

Log_10_-transformed anti-Vi IgG was analysed using a restricted maximum likelihood-based linear mixed-effects model using all available measures from the three measurement times and accounting for the correlations among the repeated measurements from the same subject, using the missing at random assumption. Analyses included vaccine group, measurement time, and interaction of vaccine group by measurement time as fixed categorical effects, and gender, age stratum, interaction of vaccine group by age stratum, interaction of age stratum by measurement time, and interaction of vaccine group by age stratums by measurement time as categorical fixed covariates. A common unstructured covariance was used to model the within-subject errors and there was no convergence issue. The Kenward-Roger approximation was used to estimate denominator degrees of freedom. The model-based estimated marginal mean IgG (log_10_ scale) and the corresponding 95% CIs were then estimated for each combination of vaccine group and age stratum at each measurement time and compared between vaccine groups and age strata with Scheffe's adjustment for multiple comparison. Analyses were implemented using mixed procedure in Stata Statistical Software, release 17.

An independent data safety monitoring board met before the study started and reviewed data every 6 months during recruitment and follow-up and no amendments were requested on the basis of these reviews. The trial is registered at ClinicalTrials.gov (NCT03299426).

### Role of the funding source

The funder of the study had no role in study design, data collection, data analysis, data interpretation, or writing of the report.

## Results

Between Feb 22, 2018, and Sept 6, 2018, we screened 664 participants, of whom 33 were ineligible. We enrolled and randomly assigned 631 participants (320 to the TCV group and 311 to the MCV-A group) and vaccinated 602 participants (305 in the TCV group and 297 in the MCV-A group). Of the 29 participants who were randomly assigned and not vaccinated, 14 in both the TCV group and MCV-A group did not have blood drawn at enrolment, and one participant from the TCV group left before vaccination. Five participants were excluded after vaccination and 597 were included in this analysis: 304 in the TVC group and 293 in the MCV-A group (figure). The age distribution of the five participants who were excluded was 9–11 months (n=2), 1–5 years (n=1), and 6–12 years (n=2). Demographic characteristics were similar between the study groups (table 1).

**Figure:**
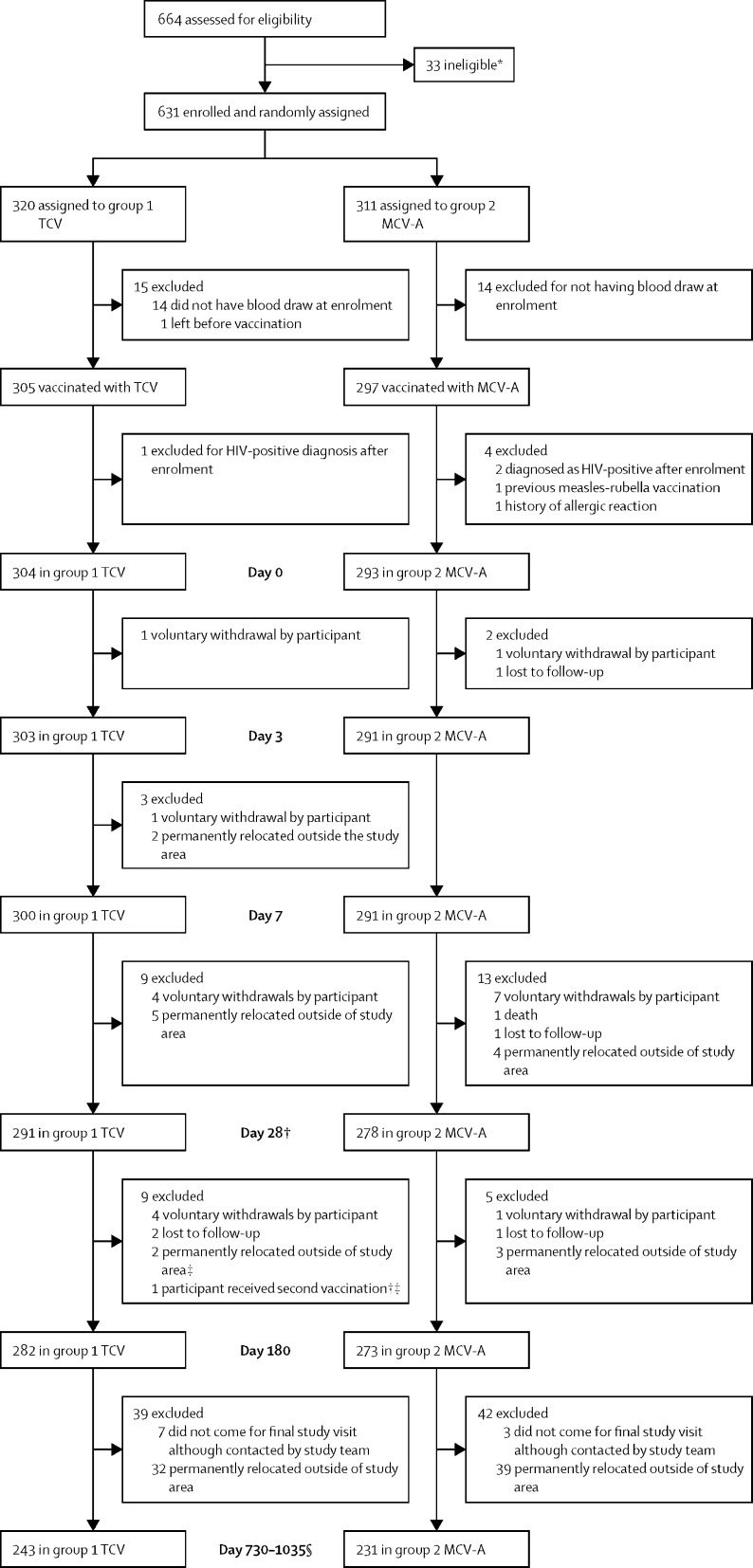
CONSORT flow diagram MCV-A=meningococcal serogroup A conjugate vaccine. TCV=typhoid conjugate vaccine. *Five participants had fever within 24 h of eligibility assessment; three participants used anti-pyretics within 4 h of assessment; nine participants had known HIV infection or exposure or other immunosuppressive conditions; three participants received measles–rubella vaccine 1 month before enrolment; one participant received measles-containing vaccine; one participant had an allergy to study or control vaccine; two participants received a systemic immunosuppressant or corticosteroids; one participant had a history of chronic illness; six participants were not included on investigator decision; and two participants did not pass the screening because of malnutrition. †26 participants were excluded from the immunogenicity analysis because had a late day-28 visit. ‡One participant was dually enrolled into the main efficacy study and did not disclose concurrent participation in the safety and immunogenicity study. §Four participants were excluded from the immunogenicity analysis because they had an early day-730–1035 visit.

**Table 1:** Summary of study, demographics, and serostatus of participants at enrolment for the intention-to-treat population

			**Group 1 TCV**	**Group 2 MCV-A**
Enrolled			320	311
Vaccinated			304	293
Sex				
	Female		135 (44%)	149 (51%)
	Male		169 (56%)	144 (49%)
Median age, years			3·0 (0·8–7·0)	3·0 (0·9–7·0)
Ethnicity				
	Black		304 (100%)	293 (100%)
Anti-Vi antibody at baseline				
	Detectable titre, ≥7·4 EU/mL		22 (7%)	24 (8%)
	GMT, EU/mL		4·2 (4·0–4·4)	4·3 (4·0–4·5)
Age stratum: 9–11 months			105 (35%)	93 (32%)
	Median weight, kilograms		8·5 (8·0–9·3)	8·8 (8·0–9·1)
	Median height, centimetres		71·0 (68·0–72·0)	70·5 (68·5–73·0)
	Median BMI		17·3 (16·3–18·5)	17·3 (16·2–18·5)
	Anti-Vi antibody at baseline			
		Detectable titre, ≥7·4 EU/mL	3 (3%)	6 (6%)
		GMT, EU/mL	3·9 (3·7–4·1)	4·0 (3·7–4·4)
	Anti-measles antibody at baseline			
		Detectable titre, >1·7 mIU/mL	103 (98%)*	93 (100%)
		GMT, mIU/mL	8·9 (7·9–10·0)	9·2 (8·0–10·6)
		Percentage seropositive, ≥120 mIU/mL	1·0 (0·2–5·3)	1·1 (0·2–5·8))
Anti-rubella antibody at baseline				
	Detectable titre, >0·1 IU/mL		97 (92%)	91 (98%)
	GMT, IU/mL		0·3 (0·3–0·4)	0·4 (0·3–0·5)
	Percentage seropositive, ≥10 IU/mL		1·0 (0·2–5·3)	2·2 (0·6–7·5)
Age stratum: 1–5 years			99 (33%)	101 (35%)
	Median weight, kilograms		13·0 (11·1–15·5)	12·4 (11·0–15·0)
	Median height, centimetres		93·0 (84·5–102·0)	90·0 (83·5–100·0)
	Median BMI		15·5 (14·4–16·4)	15·4 (14·5–16·2)
	Anti-Vi antibody at baseline			
		Detectable titre, ≥7·4 EU/mL	7 (7%)	8 (8%)
		GMT, EU/mL	4·2 (3·8–4·7)	4·4 (3·9–4·9)
Age stratum: 6–12 years			100 (33%)	99 (34%)
	Anti-Vi antibody at baseline			
		Detectable titre, ≥7·4 EU/mL	12 (12%)	10 (10%)
		GMT, EU/mL	4·5 (4·0–5·0)	4·4 (3·9–4·9)

Data are n (%), median (quartile 1–quartile 3), mean (95% CI), or percentage (95% CI). TCV=typhoid conjugate vaccine. MCV-A=meningococcal serogroup A conjugate vaccine. EU=ELISA units. GMT=geometric mean titre. IU=International units.

* Baseline titre not available for two participants.

Baseline anti-Vi IgG antibody concentrations were similar between the TCV and MCV-A groups. At day 28 after vaccination, anti-Vi IgG GMT rose by more than 500 times the baseline value in the TCV group and remained unchanged in the MCV-A group. On average, anti-Vi IgG (log_10_ transformed) did not decrease between day 730 and day 1035 in both the TCV and MCV-A groups, and therefore, we elected to analyse the days 730–1035 data together. At days 730–1035 after vaccination, anti-Vi IgG GMT values decreased to 48·0 EU/mL (95% CI 39·9–57·8) among TCV recipients, 10 times higher than MCV-A recipient amounts (table 2; appendix p 7). Similar results were seen in the per-protocol analysis (appendix p 2) and there was no difference by sex (appendix p 3).

**Table 2:** Anti-Vi immunoglobulin G antibody immunogenicity 28 days and 730–1035 days after vaccination by ELISA, in the intention-to-treat population

		**Group 1 TCV**		**Group 2 MCV-A**	
		n or n/N	Mean or percentage (95% CI)	n or n/N	Mean or percentage (95% CI)
**All age strata**					
GMT					
	Day 28	287	2383·7 (2087·2–2722·3)	275	4·4 (4·0–4·7)
	Day 730*	221	48·0 (39·9–57·8)	208	4·6 (4·2–5·0)
GMFR					
	Day 0 to 28	283	564·7 (492·3–647·8)	269	1·0 (1·0–1·1)
	Day 0 to 730*	219	11·6 (9·6–13·9)	203	1·1 (1·0–1·2)
Seroconversion four times or higher increase from:					
	Day 0 to 28	279/283	98·6 (96·4–99·5)	1/269	0·4 (0·1–2·1))
	Day 0 to 730*	175/219	79·9 (74·1–84·7)	9/203	4·4 (2·4–8·2)
**Age stratum: 9–11 months**					
GMT					
	Day 28	98	2594·8 (2115·8–3182·2)	83	4·0 (3·7–4·3)
	Day 730*	60	24·2 (18·3–31·9)	53	3·9 (3·6–4·3)
GMFR					
	Day 0 to 28	97	661·8 (534·3–819·8)	83	1·0 (0·9–1·0)
	Day 0 to 730*	60	6·2 (4·6–8·3)	53	1·0 (0·8–1·1)
Seroconversion four times or higher increase from:					
	Day 0 to 28	96/97	99·0 (94·4–99·8)	0/83	0 (0–4·4)
	Day 0 to 730*	41/60	68·3 (55·8–78·7)	1/53	1.9 (0.3–9.9)
**Age stratum: 1–5 years**					
GMT					
	Day 28	91	2085·9 (1635·6–2660·2)	99	4·6 (3·9–5·4)
	Day 730*	74	36·9 (27·1–50·3)	77	4·8 (4·1–5·5)
GMFR					
	Day 0 to 28	90	490·6 (378·6–635·6)	95	1·1 (0·9–1·2)
	Day 0 to 730*	74	8·9 (6·5–12·1)	74	1·0 (0·9–1·2)
Seroconversion four times or higher increase from:					
	Day 0 to 28	88/90	97·8 (92·3–99·4)	1/95	1·1 (0·2–5·7)
	Day 0 to 730*	58/74	78·4 (67·7–86·2)	3/74	4·1 (1·4–11·3)
**Age stratum: 6–12 years**					
GMT					
	Day 28	98	2478·7 (1953·0–3145·9)	93	4·4 (4·0–4·9)
	Day 730*	87	96·3 (73·2–126·7)	78	4·9 (4·1–5·9)
GMFR					
	Day 0 to 28	96	549·0 (434·1–694·3)	91	1·0 (0·9–1·1)
	Day 0 to 730*	85	22·9 (17·5–29·8)	76	1·2 (1·0–1·4)
Seroconversion four times or higher increase from:					
	Day 0 to 28	95/96	99·0 (94·3–99·8)	0/91	0 (0–4·1)
	Day 0 to 730*	76/85	89·4 (81·1–94·3)	5/76	6·6 (2·8–14·5)

Data are mean (95% CI) or percentage (95% CI). TCV=typhoid conjugate vaccine. MCV-A=meningococcal serogroup A conjugate vaccine. n=number of participants. N=total number. GMT=geometric mean titre. GMFR=geometric mean-fold rise.

* Day 730 visits were extended by a year because of COVID-19 restrictions (730–1035 days).

Seroconversion occurred in 279 (99%) of 283 (95% CI 96–100) TCV recipients and one (<1%) of 269 MCV-A recipients (0·1–2·1) at day 28. At days 730–1035, 175 (80%) of 219 TCV recipients (74·1–84·7) and nine (4%) of 203 MCV-A recipients (2·4–8·2) demonstrated seroconversion (table 2).

Among TCV recipients at day 28, anti-Vi IgG antibody titres and seroconversion rates among the three age strata were similar. By days 730–1035, when compared with their respective baseline titres, anti-Vi IgG GMT were highest among children in the 6–12-year stratum with an increase 22·9 times greater (95% CI 17·5–29·8) followed by the 1–5-year stratum with an increase 8·9 times greater (6·5–12·1), and finally the 9–11-month stratum with an increase 6·2 times greater (4·6–8·3; table 2; appendix p 2). The proportion of participants who showed seroconversion at days 730–1035 followed a similar pattern with greatest seroconversion in the older age stratum (table 2).

In infants aged 9–11 months at day 28 after vaccination, measles PRN seroprotection rates were similar in TCV recipients. Similar results were obtained for rubella with 73 (75%) of 97 participants (95% CI 65·8–82·8) in the TCV group reaching seroprotection versus 65 (80%) of 81 participants (70·3–87·5) in the MCV-A group (table 3; appendix pp 8–9).

**Table 3:** Anti-measles (plaque reduction neutralisation) and anti-rubella (ELISA) immunoglobulin G antibody immunogenicity 28 days after vaccination, age stratum 9–11 months, in the intention-to-treat population

	**Group 1 TCV**		**Group 2 MCV-A**	
	n or n/N	Mean or percentage (95% CI)	n or n/N	Mean or percentage (95% CI)
**Anti-measles antibody (PRN)**				
GMT (mIU/mL)	97	242·0 (201·0–291·4)	81	312·0 (251·3–387·2)
Percent seropositive (≥120 mIU/mL)	81/97	83·5% (74·9–89·6)	72/81	88·9% (80·2–94·0)
**Anti-rubella antibody (ELISA)**				
GMT (IU/mL)	97	18·2 (14·4–23·0)	81	17·5 (13·5–22·5)
Percent seropositive (≥10 IU/mL)	73/97	75·3% (65·8–82·8)	65/81	80·3% (70·3–87·5)

Data are mean (95% CI) or percentage (95% CI). TCV=typhoid conjugate vaccine. MCV-A=meningococcal serogroup A conjugate vaccine. n=number of participants. N= total number. GMT=geometric mean titre. IU=International units.

Collectively, solicited reactogenicity was similar among TCV and MCV-A recipients for both injection sites, as were systemic symptoms. Injection-site reactions on the day of vaccination included mild or moderate pain or tenderness in seven (2%) of 304 (95% CI 1·1–4·7) in the TCV group and two (<1%) of 293 (0·2–2·5) in the MCV-A group, and mild swelling only in the participants vaccinated with MCV-A (one [<1%, 0·1–1·9]). The systemic adverse events most frequently reported on the day of vaccination were subjective fever, followed by irritability and malaise. Myalgia and arthralgia only occurred in participants vaccinated with TCV (table 4; appendix pp 4–6, 11).

**Table 4:** Summary of reactogenicity and safety parameters (adverse events) by vaccine group in the intention-to-treat population

			**Group 1 TCV**	**Group 2 MCV-A**
Local reactions at injection site				
	Day 0		n=304	n=293
		Pain or tenderness	7 (2%; 1·1–4·7)	2 (<1%; 0·2–2·5)
		Swelling	0 (0%; 0–1·3)	1 (<1%; 0·1–1·9)
		Erythema	0 (0%; 0–1·3)	0 (0%; 0–1·3)
		Any local reaction	7 (2%; 1·1–4·7)	2 (<1%; 0·2–2·5)
	Day 3		n=295	n=287
		Pain or tenderness	1 (<1%; 0·1–1·9)	1 (<1%; 0·1–2·0)
		Swelling	0 (0%; 0–1·3)	0 (0%; 0–1·3)
		Erythema	0 (0%; 0–1·3)	0 (0%; 0–1·3)
		Any local reaction	1 (<1%; 0·1–1·9)	1 (<1%; 0·1–2·0)
	Day 7		n=294	n=288
		Pain or tenderness	0 (0%; 0–1·3)	0 (0%; 0–1·3)
		Swelling	1 (0·3%; 0·1–1·9)	0 (0%; 0–1·3)
		Erythema	0 (0%; 0–1·3)	0 (0%; 0–1·3)
		Any local reaction	1 (<1%; 0·1–1.9)	0 (0%; 0–1·3)
Days 0, 3, and 7				
		Any local reaction	8 (3%; 1·3–5·1)	3 (1%; 0·4–3·0)
Systemic reactions				
	Day 0		n=304	n=293
		Fever	15 (5%; 3·0–8·0)	10 (3%; 1·9–6·2)
		Irritability	9 (3%; 0·7–3·8)	3 (1%; 0·4–3·0)
		Malaise	5 (2%; 0·5–3·8)	4 (1%; 0·5–3·5)
		Myalgia	4 (1%; 0·5–3·3)	0 (0%; 0–1·3)
		Arthralgia	2 (<1%; 0·2–2·4)	0 (0%; 0–1·3)
		Any systemic reaction	18 (6%; 3·8–9·2)	12 (4%; 2·4–7·0)
	Day 3		n=295	n=287
		Fever	6 (2%; 0·9–4·4)	6 (2%; 1·0–4·5)
		Irritability	2 (<1%; 0·2–2·4)	5 (2%; 0·8–4·0)
		Malaise	2 (<1%; 0·2–2·4)	1 (<1%; 0·1–2·0)
		Myalgia	1 (<1%; 0·1–1·9)	0 (0%; 0–1·3)
		Arthralgia	1 (<1%; 0·1–1·9)	0 (0%; 0–1·3)
		Any systemic reaction	6 (2%; 0·9–4·4)	9 (3%; 1·7–5·9)
	Day 7		n=294	n–288
		Fever	9 (3%; 1·6–5·7)	11 (4%; 2·2–6·7)
		Irritability	3 (1%; 0·4–3·0)	2 (<1%; 0·2–2·5)
		Malaise	1 (<1%; 0·1–1·9)	1 (<1%; 0·1–1·9)
		Myalgia	1 (<1%; 0·1–1·9)	0 (0%; 0–1·3)
		Arthralgia	1 (<1%; 0·1–1·9)	0 (0%; 0–1·3)
		Any systemic reaction	10 (3%; 1·9–6·2)	12 (4%; 2·4–7·1)
	Days 0, 3, and 7			
		Any systemic reaction	27 (9%; 6·2–12·6)	27 (9%; 6·4–13·1)
Unsolicited adverse events			n=304	n=293
	Related		8 (3%; 1·3–5·1)	8 (3%; 1·4–5·3)
	Not Related		67 (22%; 17·7–27·0)	43 (15%; 11·1–19·2)
	Any unsolicited adverse event		74 (24%; 19·9–29·5)	49 (17%; 12·9–21·4)

Data are n (%; 95% CI). TCV=typhoid conjugate vaccine. MCV-A=meningococcal serogroup A conjugate vaccine. n=number of participants.

At day 3 after vaccination, the proportion of participants with fever was similar between the two groups and lower than on the day of vaccination. The proportion of participants with irritability remained low in both groups. By day 7 after vaccination, nine (3%) of 294 (1·6–5·7) participants vaccinated with TCV and 11 (4%) of 288 (2·2–6·7) participants vaccinated with MCV-A had fever, with irritability lower than or equal to 1% in both groups. All other solicited reactions were present in less than 1% of the participants at both 3 days and 7 days after vaccination (table 4). Unsolicited adverse events occurring within 28 days of vaccination occurred in 74 (24%) of 304 (19·9–29·5) in the TCV group and 49 (17%) of 293 (12·9–21·4) in the MCV-A group. Less than 5% of the unsolicited adverse events were considered related to vaccination and included rash, fever with no source, painful right arm, diarrhoea, and upper respiratory illness. Related unsolicited adverse events occurred at a similar frequency between participants vaccinated with TCV and MCV-A (table 4; appendix pp 4–6).

## Discussion

In this nested substudy of a randomised, controlled trial, TCV was safe, well tolerated, and immunogenic in Malawian children aged 9 months to 12 years. Furthermore, in children younger than 1 year of age, TCV showed strong immunogenicity and no interference or safety concerns when coadministered with measles–rubella vaccine.

Our findings in Malawian children are consistent with results from four other clinical trials of Typbar TCV in Africa and Asia. The first paediatric immunogenicity data generated in Indian participants showed a 98% seroconversion rate in children aged 6–11 months, 99% in children aged 2–4 years, and 99% in children aged 5–15 years, 42 days after vaccination, and a 59% seroconversion rate in children aged 6–11 months, 77% in children aged 2–4 years, and 75% in children aged 5–15 years, 720 days after vaccination.^14^ The Typhoid Vaccine Acceleration Consortium (TyVAC) trials of Typbar TCV in Nepalese and Bangladeshi children aged 9 months to 15 years each showed a 99% seroconversion rate at 28 days after vaccination,^24^, ^25^ which is similar to the 98% seroconversion rate at day 28 in this study. More recently, Typbar TCV was shown to be immunogenic in Burkinabe infants aged 9–11 months (88%) 28 days after vaccination.^26^ A similar TCV, produced by another manufacturer, which is also conjugated to tetanus toxoid and licensed for use in India, showed a 100% seroconversion in Indian children from 6 months to 12 years of age 42 days after vaccination, and an 84% seroconversion rate 12 months after vaccination.^27^ In the five aforementioned trials,^14^, ^24^, ^25^, ^26^, ^27^ TCVs were well tolerated with mild reactogenicity, most commonly fever and pain at injection site.

The WHO recommendation, for programmatic use of TCV to control typhoid fever, encourages reliance on local data when making decisions on the age of administration, target population, and delivery strategy for routine and catch-up campaigns.^15^ In typhoid-endemic regions, WHO suggests TCV administration at 9 months of age, or within the first 2 years of life, as part of routine childhood immunisation, to provide early protection.^15^ Additionally, if children are vaccinated at a young age, when programmatically feasible, the duration of protection is even more important because exposure will persist for many more years.

In this substudy, most infants who received TCV with routine measles–rubella vaccine successfully achieved anti-Vi seroconversion at day 28 after vaccination (99%) and 68% were seroconverted 2–3 years after vaccination. By contrast, 89% of children in the 6–12-year age stratum were seroconverted at 730–1035 days, although variation in collection times could have influenced this result in either group. As no correlate of protection exists for TCV, it is unclear whether decreased seroconversion indicates diminished protection, a need for a TCV booster, or both. Ongoing and planned studies of booster vaccination will help to answer this question.

Infants who received TCV also successfully achieved seroprotective amounts of anti-measles and anti-rubella antibodies. The 84% measles seroprotection rate in this study is similar to the 85% rate previously reported in Malawian infants unexposed to HIV 90 days after vaccination who received measles vaccine at 9 months of age.^28^ Overall, measles seroprotection in our trial after the first dose of measles–rubella vaccine supports the WHO recommendation for a two-dose vaccine schedule, which was introduced in 2015 in the routine vaccination schedule in Malawi.

In addition to routine vaccination with TCV, WHO recommends catch-up campaigns for older children, when feasible, to accelerate vaccine impact and potentially increase indirect protection of unvaccinated individuals. Vaccinating school-age children through schools is a delivery strategy that can lead to high coverage.^29^ Our data confirm that school-age children achieve and maintain elevated anti-Vi immunogenicity. The feasibility and effect of TCV vaccination of children in a community setting was shown in 2019 when Zimbabwe became the first African country to use TCV outside a study setting.^30^

These results also show that TCV has a favourable tolerability and reactogenicity profile that is similar to our control vaccine, MCV-A, a vaccine used routinely in many African countries. Consequently, administration of TCV at regular child health visits facilitates community acceptance and requires fewer health-care resources. For these reasons, incorporating TCV into routine vaccination schedules is advantageous.

One strength of this trial is that it is nested within an ongoing efficacy study that will yield essential information on TCV performance, particularly in young children. This is especially important because an immunological correlate of protection has not yet been established for typhoid fever. A four-fold rise in GMT at 1 month after vaccination has been used to define seroconversion for previous TCV trials and has been associated with field efficacy at the population level. Published data from the Malawi trial showed an ITT efficacy of 80·7% against blood-culture-confirmed typhoid fever after 18–24 months of follow-up among 28 130 Malawian children. Similarly, in Bangladesh, TCV effectiveness was 85% among children aged 9 months to 15 years and vaccine protection was consistent in different age strata, including children younger than 2 years of age.^25^

As the children in this study remain in the main efficacy trial, our study has not yet assessed the effect of a booster dose on longevity of seroprotection from TCV. Based on previous TCV studies, seroprotection likely persists for at least 5 years.^14^ A limitation of this study is that is provides safety and immunogenicity data only in children unexposed to HIV and does not include children exposed to HIV who remain uninfected, an important population in sub-Saharan Africa. Additional studies of safety and immunogenicity in African children are ongoing and will further characterise the immunogenicity, safety, and tolerability of two doses of TCV, and the longer-term safety and immunogenicity of single-dose TCV in different populations, including infants exposed to HIV who are uninfected. These follow-up studies will further inform programmatic decisions for TCV implementation, including the need for two-dose or booster-dose schedules.

This clinical trial presents evidence of TCV safety and immunogenicity in African children across a wide age range at 730 days and beyond, and includes coadministration data with the measles–rubella vaccine. Results are especially valuable to African countries considering TCV introduction into routine childhood immunisation, with or without catch-up campaigns, and for outbreak response.

## Data sharing

All of the individual participant data collected during the trial will be available beginning 9 months and ending 36 months following publication to researchers who provide a methodologically sound proposal to achieve aims in the approved proposal. Proposals should be directed to mlaurens@som.umaryland.edu.

## Declaration of interests

We declare no competing interests.

## References

[1] Brockett S, Wolfe MK, Hamot A, Appiah GD, Mintz ED, Lantagne D (2020). Associations among water, sanitation, and hygiene, and food exposures and typhoid fever in case-control studies: a systematic review and meta-analysis. Am J Trop Med Hyg.

[2] Dougan G, Baker S (2014). *Salmonella enterica* serovar typhi and the pathogenesis of typhoid fever. Annu Rev Microbiol.

[3] Ajibola O, Mshelia MB, Gulumbe BH, Eze AA (2018). Typhoid fever diagnosis in endemic countries: a clog in the wheel of progress?. Medicina.

[4] Cruz Espinoza LM, McCreedy E, Holm M (2019). Occurrence of typhoid fever complications and their relation to duration of illness preceding hospitalization: a systematic literature review and meta-analysis. Clin Infect Dis.

[5] Birkhold M, Coulibaly Y, Coulibaly O (2020). Morbidity and mortality of typhoid intestinal perforation among children in sub-Saharan Africa 1995–2019: a scoping review. World J Surg.

[6] Gunn JS, Marshall JM, Baker S, Dongol S, Charles RC, Ryan ET (2014). Salmonella chronic carriage: epidemiology, diagnosis, and gallbladder persistence. Trends Microbiol.

[7] Feasey NA, Gaskell K, Wong V (2015). Rapid emergence of multidrug resistant, H58-lineage *Salmonella typhi* in Blantyre, Malawi. PLoS Negl Trop Dis.

[8] Klemm EJ, Shakoor S, Page AJ (2018). Emergence of an extensively drug-resistant *Salmonella enterica* serovar typhi clone harboring a promiscuous plasmid encoding resistance to fluoroquinolones and third-generation cephalosporins. MBio.

[9] Global Burden of Disease Collaborative Network (2020). Global burden of disease, typhoid fever—level 4 cause. http://www.healthdata.org/results/gbd_summaries/2019/typhoid-fever-level-4-cause.

[10] Britto C, Pollard AJ, Voysey M, Blohmke CJ (2017). An appraisal of the clinical features of pediatric enteric fever: systematic review and meta-analysis of the age-stratified disease occurrence. Clin Infect Dis.

[11] Meiring JE, Shakya M, Khanam F (2021). Burden of enteric fever at three urban sites in Africa and Asia: a multicentre population-based study. Lancet Glob Health.

[12] Barac R, Als D, Radhakrishnan A, Gaffey MF, Bhutta ZA, Barwick M (2018). Implementation of interventions for the control of typhoid fever in low- and middle-income countries. Am J Trop Med Hyg.

[13] Khan MI, Franco-Paredes C, Sahastrabuddhe S, Ochiai RL, Mogasale V, Gessner BD (2017). Barriers to typhoid fever vaccine access in endemic countries. Res Rep Trop Med.

[14] Mohan VK, Varanasi V, Singh A (2015). Safety and immunogenicity of a Vi polysaccharide-tetanus toxoid conjugate vaccine (Typbar-TCV) in healthy infants, children, and adults in typhoid endemic areas: a multicenter, 2-cohort, open-label, double-blind, randomized controlled phase 3 study. Clin Infect Dis.

[15] WHO (2018). Typhoid vaccines: WHO position paper, March 2018: recommendations. Vaccine.

[16] The SAGE Working Group on Typhoid Vaccines WS (2017).

[17] Patel PD, Patel P, Liang Y (2021). Safety and efficacy of a typhoid conjugate vaccine in Malawian children. N Engl J Med.

[18] Meiring JE, Laurens MB, Patel P (2019). Typhoid vaccine acceleration consortium Malawi: a phase III, randomized, double-blind, controlled trial of the clinical efficacy of typhoid conjugate vaccine among children in Blantyre, Malawi. Clin Infect Dis.

[19] Cohen BJ, Audet S, Andrews N, Beeler J (2007). Plaque reduction neutralization test for measles antibodies: description of a standardised laboratory method for use in immunogenicity studies of aerosol vaccination. Vaccine.

[20] Simon JK, Ramirez K, Cuberos L (2011). Mucosal IgA responses in healthy adult volunteers following intranasal spray delivery of a live attenuated measles vaccine. Clin Vaccine Immunol.

[21] Tapia MD, Sow SO, Medina-Moreno S (2005). A serosurvey to identify the window of vulnerability to wild-type measles among infants in rural Mali. Am J Trop Med Hyg.

[22] WHO (2011). Rubella vaccines: WHO position paper. Recommendations. Vaccine.

[23] WHO (2019). Measles vaccines: WHO position paper, April 2017. Recommendations. Vaccine.

[24] Shakya M, Colin-Jones R, Theiss-Nyland K (2019). Phase 3 efficacy analysis of a typhoid conjugate vaccine trial in Nepal. N Engl J Med.

[25] Qadri F, Khanam F, Liu X (2021). Protection by vaccination of children against typhoid fever with a Vi-tetanus toxoid conjugate vaccine in urban Bangladesh: a cluster-randomised trial. Lancet.

[26] Sirima SB, Ouedraogo A, Barry N (2021). Safety and immunogenicity of Vi-typhoid conjugate vaccine co-administration with routine 9-month vaccination in Burkina Faso: a randomized controlled phase 2 trial. Int J Infect Dis.

[27] Mitra M, Shah N, Ghosh A (2016). Efficacy and safety of Vi-tetanus toxoid conjugated typhoid vaccine (PedaTyph™) in Indian children: school based cluster randomized study. Hum Vaccin Immunother.

[28] Fowlkes A, Witte D, Beeler J (2011). Persistence of vaccine-induced measles antibody beyond age 12 months: a comparison of response to one and two doses of Edmonston-Zagreb measles vaccine among HIV-infected and uninfected children in Malawi. J Infect Dis.

[29] Meiring JE, Gibani M, Ty VACCMG (2017). The Typhoid Vaccine Acceleration Consortium (TyVAC): vaccine effectiveness study designs: accelerating the introduction of typhoid conjugate vaccines and reducing the global burden of enteric fever. Report from a meeting held on 26–27 October 2016, Oxford, UK. Vaccine.

[30] Olaru ID, Mtapuri-Zinyowera S, Feasey N, Ferrand RA, Kranzer K (2019). Typhoid Vi-conjugate vaccine for outbreak control in Zimbabwe. Lancet Infect Dis.

