# Supplementary material for: Safety and immunogenicity of a typhoid conjugate vaccine among children aged 9 months to 12 years in Malawi: a nested substudy of a double-blind, randomised controlled trial
Source: Lancet Glob Health. 2022 Aug 9;10(9):e1326–35. doi: 10.1016/S2214-109X(22)00275-3 (PMC9380257; doi:10.1016/S2214-109X(22)00275-3)
Supplement: Supplementary appendix [file mmc1.pdf]

THE LANCET
Global Health

Supplementary appendix

This appendix formed part of the original submission and has been peer reviewed. We post it as supplied by the authors.

Supplement to: Nampota-Nkomba N, Nyirenda OM, Khonde L, et al. Safety and immunogenicity of a typhoid conjugate vaccine among children aged 9 months to 12 years in Malawi: a nested substudy of a double-blind, randomised controlled trial. *Lancet Glob Health* 2022; **10:** e1326–35.

Eligibility criteria for the main efficacy trial

Inclusion criteria

1. A healthy male or female child between the ages of 9 months and 12 years/364 days at the time of study vaccination.
2. A child whose parent or guardian resides primarily within the Ndirande or Zingwangwa study areas at the time of study vaccinations, and who intends to be present in the area for the duration of the trial.
3. A child whose parent or guardian has voluntarily given informed consent.

Exclusion criteria

1. A history of documented hypersensitivity to any component of the vaccine.
2. Prior receipt of any typhoid vaccine in the past three years.
3. A history of a severe allergic reaction with generalized urticarial, angioedema, or anaphylaxis.
4. Any condition determined by the investigator to be likely to interfere with evaluation of the vaccine, to be a significant potential health risk to the child, or to make it unlikely that the child would complete the study.

Temporary exclusion criteria

The following will be considered temporary contraindications to enrollment and vaccination. If these apply, the participant will be temporarily excluded for vaccination until 48 hours has passed. A reassessment will be needed to ensure these temporary exclusion criteria no longer exist.

1. Reported fever within 24 hours prior to vaccination.
2. Use of anti-pyretics within 4 hours prior to vaccination.
3. Receipt of measles-rubella vaccine in the 1 month prior to enrollment, as determined by parental history or vaccination card.

Additional exclusion criteria for immunogenicity and reactogenicity sub-study

1. A known history of diabetes, tuberculosis, cancer, chronic kidney disease, heart disease, liver disease, a progressive neurological disorder, poorly controlled seizures, or a terminal illness.
2. Severe malnutrition, as determined by mid-upper arm circumference < 12.5 cm for children younger than 5 years.
3. The receipt of any other investigational intervention in the prior 6 months or the anticipated receipt during the course of the study.
4. The receipt of blood products in the last 6 months.
5. A known human immunodeficiency virus infection or exposure, or any other immunosuppressive condition.
6. The receipt of systemic immunosuppressants or systemic corticosteroids.
7. The receipt of any measles-rubella–containing vaccine for children younger than 1 year of age.

**Table A1· Anti-Vi IgG antibody immunogenicity 28 and 730-1035 days after vaccination by ELISA in the per protocol population**				
Data are mean (95% CI) or % (95% CI). n=number of participants. N= total number. GMT=geometric mean titre. GMFR=geometric mean-fold rise.				
†Day 730 visits were extended by a year due to COVID-19 restrictions (730-1035 days)				
**Anti-Vi IgG Antibody (ELISA)**	**Group 1: TCV**		**Group 2: MCV-A**	
	**n or n/N**	**Mean or % (95% CI)**	**n or n/N**	**Mean or % (95% CI)**
**All Age Strata**				
**GMT**				
**Day 28**	272	2399·8 (2089·0-2756·8)	265	4·4 (4·1-4·8)
**Day 730†**	221	48·0 (39·9-57·8)	204	4·6 (4·2-5·2)
**GMFR**				
**Day 0 to 28**	269	567·8 (491·9-655·4)	259	1·0 (1·0-1·1)
**Day 0 to730†**	219	11·6 (9·6-13·9)	199	1·1 (1·0-1·2)
**Seroconversion ≥4-fold increase from**				
**Day 0 to 28**	265/269	98·5 (96·2-99·4)	1/259	0·4 (0·1-2·2))
**Day 0 to730†**	175/219	79·9 (74·1-84·7)	9/199	4·5 (2·4-8·4)
**Age Stratum: 9-11 months**				
**GMT**				
**Day 28**	91	2685·0 (2164·8-3330·2)	77	4·1 (3·7-4·4)
**Day 730†**	60	24·2 (18·3-31·9)	53	3·9 (3·6-4·3)
**GMFR**				
**Day 0 to 28**	90	682·0 (543·6-855·6)	77	1·0 (0·9-1·1)
**Day 0 to 730†**	60	6·2 (4·6-8·3)	53	1·0 (0·8-1·1)
**Seroconversion ≥4-fold increase from**				
**Day 0 to 28**	89/90	99·0 (94·0-99·8)	0/77	0·0 (0·0-4·8)
**Day 0 to730†**	41/60	68·3 (55·8-78·7)	1/53	1.9 (0.3-9.9)
**Age Stratum: 1-5 years**				
**GMT**				
**Day 28**	87	2089·0 (1621·6-2691·1)	95	4·6 (3·9-5·5)
**Day 730†**	74	36·9 (27·1-50·3)	74	4·8 (4·1-5·6)
**GMFR**				
**Day 0 to 28**	86	488·1 (372·7-639·4)	91	1·1 (0·9-1·2)
**Day 0 to 730†**	74	8·9 (6·5-12·1)	71	1·0 (0·9-1·2)
**Seroconversion ≥4-fold increase from**				
**Day 0 to 28**	84/86	97·7 (91·9-99·4)	1/91	1·1 (0·2-6·0)
**Day 0 to730†**	58/74	78·4 (67·7-86·2)	3/71	4·2 (1·5-11·7)
**Age Stratum: 6-12 years**				
**GMT**				
**Day 28**	94	2447·4 (1914·1-3129·4)	93	4·4 (4·0-4·9)
**Day 730†**	87	96·3 (73·2-126·7)	77	4·9 (4·1-6·0)
**GMFR**				
**Day 0 to 28**	93	547·0 (429·6-696·4)	91	1·0 (0·9-1·1)
**Day 0 to 730†**	85	22·9 (17·5-29·8)	75	1·2 (1·0-1·4)
**Seroconversion ≥4-fold increase from**				
**Day 0 to 28**	92/93	98·9 (94·2-99·8)	0/91	0·0 (0·0-4·1)
**Day 0 to730†**	76/85	89·4 (81·1-94·3)	5/75	6·7 (2·9-14·7)

Table A2 Anti-Vi IgG antibody immunogenicity before vaccination (day 0), 28 and 730 days after vaccination, by sex in per protocol population				
Data are mean (95% CI) n=number of participants. GMT=geometric mean titre. CI=confidence interval.				
†Day 730 visits were extended by a year due to COVID-19 restrictions (730-1035 days)				
	**Group 1: TCV**		**Group 2: MCV-A**	
	**n**	**GMT (95% CI)**	**n**	**GMT (95% CI)**
**Male**				
Day 0	168	4·2 (3·9-4·5)	140	4·3 (3·9-4·6)
Day 28	152	2482·6 (2117·7-2910·3)	131	4·3 (3·9-4·6)
Day 730†	120	51·1 (40·6-64·4)	95	4·6 (4·1-5·2)
**Female**				
Day 0	132	4·2 (3·8- 4·5)	147	4·3 (3·9-4·7)
Day 28	120	2298·9 (1808·4-2922·3)	134	4·5 (4·0-5·1)
Day 730†	101	44·5 (33·1-59·8)	109	4·6 (4·0-5·3)

Table A3. Anti-Measles (PRN) and Anti-Rubella (ELISA) IgG antibody immunogenicity 28 days after vaccination, age stratum 9-11 months in per protocol population				
Data are mean (95% CI) or % (95% CI). n=number of participants. N= total number. GMT=geometric mean titre. CI= confidence intervals.				
	**Group 1: TCV**		**Group 2: MCV-A**	
	**n or n/N**	**Mean or % (95% CI)**	**n or n/N**	**Mean or % (95% CI)**
**Anti-Measles Antibody (PRN)**				
**GMT (mIU/mL)**	90	250·9 (206·4-304·9)	75	304·1 (242·3-381·7)
**Percent Seropositive (≥120 mIU/mL)**	76/90	84·4 (75·6-90·5)	67/75	89·3 (80·3-94·5)
**Anti-rubella Antibody (ELISA)**				
**GMT (IU/mL)**	90	17·4 (13·6-22·3)	75	16·5 (12·6-21·4)
**Percent Seropositive (≥10 IU/mL)**	66/90	73·3 (63·4-81·4)	60/75	80·0 (69·6-87·5)

Table A4. Summary of reactogenicity and safety parameters (adverse events) by vaccine group and age stratum 9-11 months in the ITT population

Data are n (%, 95%CI). n=number of participants. CI=confidence interval.

		Group 1: TCV	Group 2: MCV-A
**Age Stratum: 9-11 months**			
**Local Reactions at injection site**			
**Day 0**		n=105	n=93
	**Pain/tenderness**	4 (3·8, 1·5-9·4)	1 (1·1, 0·2-5·8)
	**Swelling**	0 (0·0, 0·0-3·5)	0 (0·0, 0·0-4·0)
	**Erythema**	0 (0·0, 0·0-3·5)	0 (0·0, 0·0-4·0)
	**Any local reaction**	4 (3·8, 1·5-9·4)	1 (1·1, 0·2-5·8)
**Day 3**		n=98	n=90
	**Pain/tenderness**	1 (1·0, 0·2-5·6)	0 (0·0, 0·0-4·1)
	**Swelling**	0 (0·0, 0·0-3·8)	0 (0·0, 0·0-4·1)
	**Erythema**	0 (0·0, 0·0-3·8)	0 (0·0, 0·0-4·1)
	**Any local reaction**	1 (1·0, 0·2-5·6)	0 (0·0, 0·0-4·1)
**Day 7**		n=101	n=89
	**Pain/tenderness**	0 (0·0, 0·0-3·7)	0 (0·0, 0·0-4·1)
	**Swelling**	0 (0·0, 0·0-3·7)	0 (0·0, 0·0-4·1)
	**Erythema**	0 (0·0, 0·0-3·7)	0 (0·0, 0·0-4·1)
	**Any local reaction**	0 (0·0, 0·0-3.7)	0 (0·0, 0·0-4·1)
**Days 0, 3, and 7**	**Any local reaction**	4 (3·8, 1·5-9·4)	1 (1·1, 0·2-5·8)
**Systemic Reactions**			
**Day 0**		n=105	n=93
	**Fever**	10 (9·5, 5·3-16·7)	6 (6·5, 3·0-13·4)
	**Irritability**	6 (5·7, 2·6-11·9)	1 (1·1, 0·2-5·8)
	**Malaise**	3 (2·9, 1·0-8·1)	3 (3·2, 1·1-9·1)
	**Myalgia**	1 (1·0, 0·2-5·2)	0 (0·0, 0·0-4·0)
	**Arthralgia**	1 (1·0, 0·2-5·2)	0 (0·0, 0·0-4·0)
	**Any systemic reaction**	10 (9·5, 5·3-16·7)	8 (8·6, 4·4-16·1)
**Day 3**		n=98	n=90
	**Fever**	4 (4·1, 1·6-10·0)	5 (5·6, 2·4-12·4)
	**Irritability**	1 (1·0, 0·2-5·6)	3 (3·3, 1·1-9·4)
	**Malaise**	1 (1·0, 0·2-5·6)	1(1·1, 0·2-6·0)
	**Myalgia**	0 (0·0, 0·0-3·8)	0 (0·0, 0·0-4·1)
	**Arthralgia**	0 (0·0, 0·0-3·8)	0 (0·0, 0·0-0-4·1)
	**Any systemic reaction**	4 (4·1, 1·6-10·0)	7 (7·8, 3·8-15·2)
**Day 7**		n=101	n=89
	**Fever**	8 (7·9, 4·1-14·9)	6 (6·7, 3·1-13·9)
	**Irritability**	3 (3·0, 1·0-8·4)	2 (2·3, 0·6-7·8)
	**Malaise**	1 (1·0, 0·2-5·4)	1 (1·1, 0·2-6·1)
	**Myalgia**	1 (1·0, 0·2-5·4)	0 (0·0, 0·0-4·1)
	**Arthralgia**	1 (1·0, 0·2-5·4)	0 (0·0, 0·0-4·1)
	**Any systemic reaction**	9 (8·9, 4·8-16·1)	7 (7·9, 3·9-15·4)
**Days 0, 3, and 7**	**Any systemic reaction**	18 (17·1, 11·1-25·5)	19 (20·4, 13·5-29·8)
**Adverse Events, unsolicited**			
	**Related**	3 (2·9, 1·0-8·1)	3 (3·2, 1·1-9·1)
	**Not Related**	30 (28·6, 20·8-37·9)	16 (17·2, 10·9-26·1)
	**Any Adverse Event, unsolicited**	33 (31·4, 23·3-40·8)	18 (19·4, 12·6-28·5)

Table A5. Summary of reactogenicity and safety parameters (adverse events) by vaccine group and age stratum 1-5 years in the ITT population			
Data are n (%, 95%CI). n=number of participants. CI=confidence interval.			
		**Group 1: TCV**	**Group 2: MCV-A**
**Age Stratum: 1-5 years**			
**Local Reactions at injection site**			
**Day 0**		n=99	n=101
	**Pain/tenderness**	0 (0·0, 0·0-3·7)	0 (0·0, 0·0-3·7)
	**Swelling**	0 (0·0, 0·0-3·7)	0 (0·0, 0·0-3·7)
	**Erythema**	0 (0·0, 0·0-3·7)	0 (0·0, 0·0-3·7)
	**Any local reaction**	0 (0·0, 0·0-0·0)	0 (0·7, 0·1-2·4)
**Day 3**		n=97	n=100
	**Pain/tenderness**	0 (0·0, 0·0-3·8)	0 (0·0, 0·0-3·7)
	**Swelling**	0 (0·0, 0·0-3·8)	0 (0·0, 0·0-3·7)
	**Erythema**	0 (0·0, 0·0-3·8)	0 (0·0, 0·0-3·7)
	**Any local reaction**	0 (0·0, 0·0-3·8)	0 (0·0, 0·0-3·7)
**Day 7**		n=95	n=100
	**Pain/tenderness**	0 (0·0, 0·0-3·9)	0 (0·0, 0·0-3·7)
	**Swelling**	1 (1·1, 0·2-5·7)	0 (0·0, 0·0-3·7)
	**Erythema**	0 (0·0, 0·0-3·9)	0 (0·0, 0·0-3·7)
	**Any local reaction**	1 (1·1, 0·2-5.7)	0 (0·0, 0·0-3·7)
**Days 0, 3, and 7**	**Any local reaction**	1 (1·0, 0·1-5·5)	0 (0·0, 0·0-3·7)
**Systemic Reactions**			
**Day 0**		n=99	n=101
	**Fever**	3 (3·0, 1·0-8·5)	4 (4·0, 1·6-9·7)
	**Irritability**	2 (2·0, 0·6-7·1)	2 (2·0, 0·5-6·9)
	**Malaise**	1 (1·0, 0·2-5·5)	1 (1·0, 0·2-5·4)
	**Myalgia**	1 (1·0, 0·2-5·5)	0 (0·0, 0·0-3·7)
	**Arthralgia**	0 (0·0, 0·0-3·7)	0 (0·0, 0·0-3·7)
	**Any systemic reaction**	5 (5·1, 2·2-11·3)	4 (4·0, 1·6-9·7)
**Day 3**		n=97	n=100
	**Fever**	1 (1·0, 0·2-5·6)	1 (1·0, 0·2-5·5)
	**Irritability**	0 (0·0, 0·0-3·8)	2 (2·0, 0·6-7·0)
	**Malaise**	0 (0·0, 0·0-3·8)	0 (0·0, 0·0-3·7)
	**Myalgia**	0 (0·0, 0·0-3·8)	0 (0·0, 0·0-3·7)
	**Arthralgia**	0 (0·0, 0·0-3·8)	0 (0·0, 0·0-3·7)
	**Any systemic reaction**	1 (1·0, 0·2-5·6)	2 (2·0, 0·6-7·0)
**Day 7**		n=95	n=100
	**Fever**	1 (1·1, 0·2-5·7)	4 (4·0, 1·6-9·8)
	**Irritability**	0 (0·0, 0·0-3·9)	0 (0·0, 0·0-3·7)
	**Malaise**	0 (0·0, 0·0-3·9)	0 (0·0, 0·0-3·7)
	**Myalgia**	0 (0·0, 0·0-3·9)	0 (0·0, 0·0-3·7)
	**Arthralgia**	0 (0·0, 0·0-3·9)	0 (0·0, 0·0-3·7)
	**Any systemic reaction**	1 (1·1, 0·2-5·7)	4 (4·0, 1·6-9·8)
**Days 0, 3, and 7**	**Any systemic reaction**	6 (6·1, 2·8-12·6)	7 (6·9, 3·4-13·6)
**Adverse Events, unsolicited**			
	**Related**	4 (4·0, 1·6-9·9)	1 (1·0, 0·2-5·4)
	**Not Related**	24 (24·2, 16·9-33·5)	21 (20·8, 14·0-29·7)
	**Any Adverse Event, unsolicited**	27 (27·3, 19·5-36·8)	22 (21·8, 14·9-30·8)

**Table A6. Summary of reactogenicity and safety parameters (adverse events) by vaccine group and age stratum 6-12 years in the ITT population**			
Data are n (%, 95%CI). n=number of participants. CI=confidence interval			
		**Group 1: TCV**	**Group 2: MCV-A**
**Age Stratum: 6-12 years**			
**Local Reactions at injection site**			
**Day 0**		n=100	n=99
	**Pain/tenderness**	3 (3·0, 1·0-8·5)	1 (1·0, 0·2-5·5)
	**Swelling**	0 (0·0, 0·0-3·7)	1 (1·0, 0·2-5·5)
	**Erythema**	0 (0·0, 0·0-3·7)	0 (0·0, 0·0-3·7)
	**Any local reaction**	3 (3·0, 1·0-8·5)	1 (1·0, 0·2-5·5)
**Day 3**		n=100	n=97
	**Pain/tenderness**	0 (0·0, 0·0-3·7)	1 (1·0, 0·2-5·6)
	**Swelling**	0 (0·0, 0·0-3·7)	0 (0·0, 0·0-3·8)
	**Erythema**	0 (0·0, 0·0-3·7)	0 (0·0, 0·0-3·8)
	**Any local reaction**	0 (0·0, 0·0-3·7)	1 (1·0, 0·2-5·6)
**Day 7**		n=98	n=99
	**Pain/tenderness**	0 (0·0, 0·0-3·8)	0 (0·0, 0·0-3·7)
	**Swelling**	0 (0·0, 0·0-3·8)	0 (0·0, 0·0-3·7)
	**Erythema**	0 (0·0, 0·0-3·8)	0 (0·0, 0·0-3·7)
	**Any local reaction**	0 (0·0, 0·0-3.8)	0 (0·0, 0·0-3·7)
**Days 0, 3, and 7**	**Any local reaction**	3 (3·0, 1·0-8·5)	2 (2·0, 0·6-7·1)
**Systemic Reactions**			
**Day 0**		n=100	n=99
	**Fever**	2 (2·0, 0·6-7·0)	0 (0·0, 0·0-3·7)
	**Irritability**	1 (1·0, 0·2-5·5)	0 (0·0, 0·0-3·7)
	**Malaise**	1 (1·0, 0·2-5·6)	0 (0·0, 0·0-3·7)
	**Myalgia**	2 (2·0, 0·6-7·0)	0 (0·0, 0·0-3·7)
	**Arthralgia**	1 (1·0, 0·2-5·5)	0 (0·0, 0·0-3·7)
	**Any systemic reaction**	3 (3·0, 1·0-8·5)	0 (0·0, 0·0-3·7)
**Day 3**		n=100	n=97
	**Fever**	1 (1·0, 0·2-5·5)	0 (0·0, 0·0-3·8)
	**Irritability**	1 (1·0, 0·2-5·5)	0 (0·0, 0·0-3·8)
	**Malaise**	1 (1·0, 0·2-5·5)	0 (0·0, 0·0-3·8)
	**Myalgia**	1 (1·0, 0·2-5·5)	0 (0·0, 0·0-3·8)
	**Arthralgia**	1 (1·0, 0·2-5·5)	0 (0·0, 0·0-3·8)
	**Any systemic reaction**	1 (1·0, 0·2-5·5)	0 (0·0, 0·0-0·0)
**Day 7**		n=98	n=99
	**Fever**	0 (0·0, 0·0-3·8)	1 (1·1, 0·2-5·5)
	**Irritability**	0 (0·0, 0·0-3·8)	0 (0·0, 0·0-3·7)
	**Malaise**	0 (0·0, 0·0-3·8)	0 (0·0, 0·0-3·7)
	**Myalgia**	0 (0·0, 0·0-3·8)	0 (0·0, 0·0-3·7)
	**Arthralgia**	0 (0·0, 0·0-3·8)	0 (0·0, 0·0-3·7)
	**Any systemic reaction**	0 (0·0, 0·0-3·8)	1 (1·1, 0·2-5·5)
**Days 0, 3, and 7**	**Any systemic reaction**	3 (3·0, 1·0-8·5)	1 (1·0, 0·2-5·5)
**Adverse Events, unsolicited**			
	**Related**	1 (1·0, 0·2-5·6)	4 (4·0, 1·6-9·9)
	**Not Related**	13 (13·0, 7·8-21·0)	6 (6·1, 2·8-12·6)
	**Any Adverse Event, unsolicited**	14 (14·0, 8·5-22·1)	9 (9·1, 4·9-16·4)

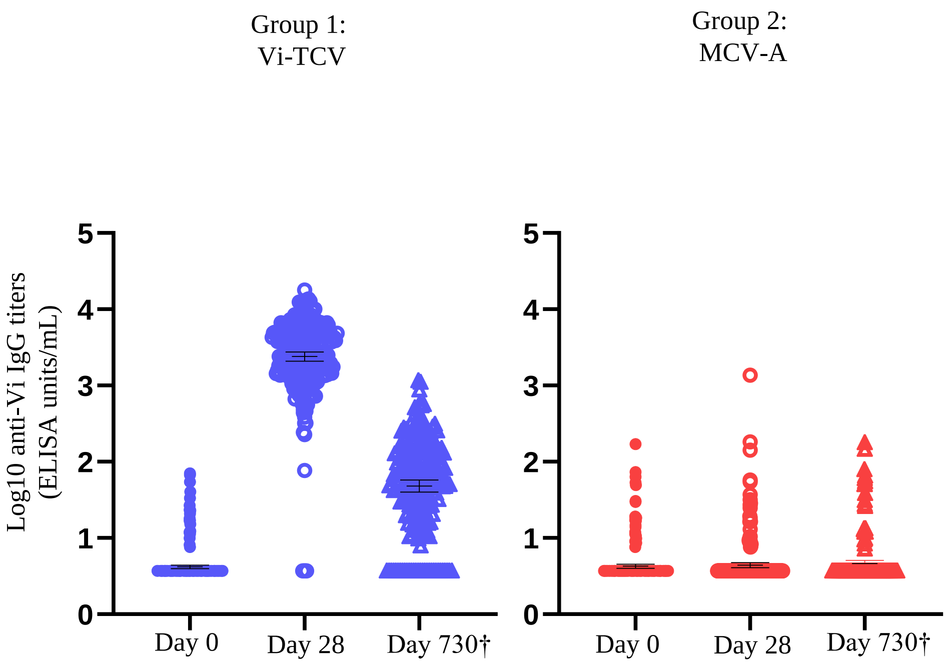

Supplemental Figure A1a. Anti-Vi IgG antibody titres before vaccination (day 0), 28 and 730-1035 days after vaccination

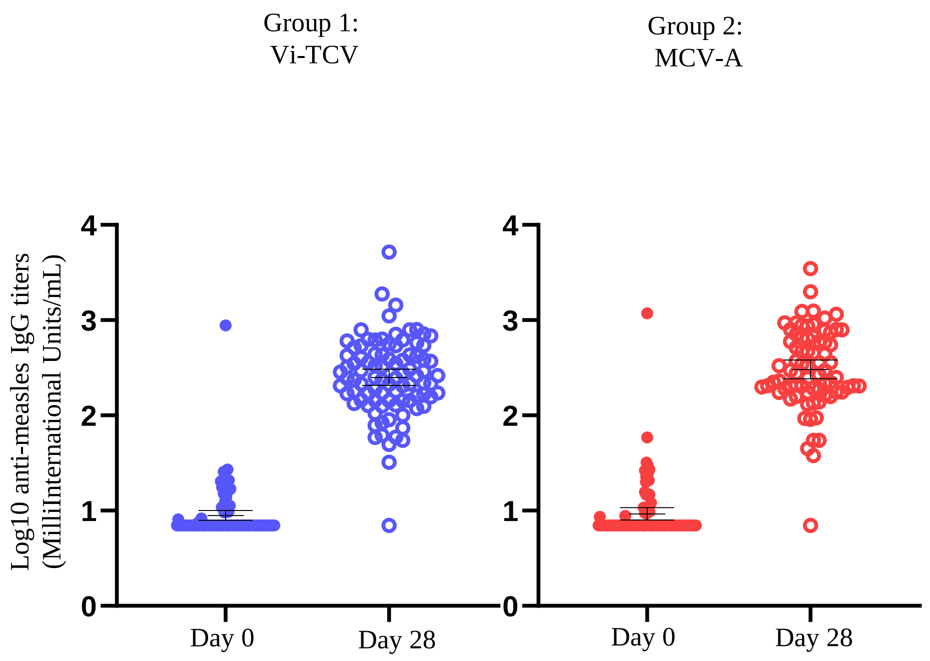

Supplemental Figure A1b. Anti-Measles antibody titres before vaccination (day 0) and 28 days after vaccination

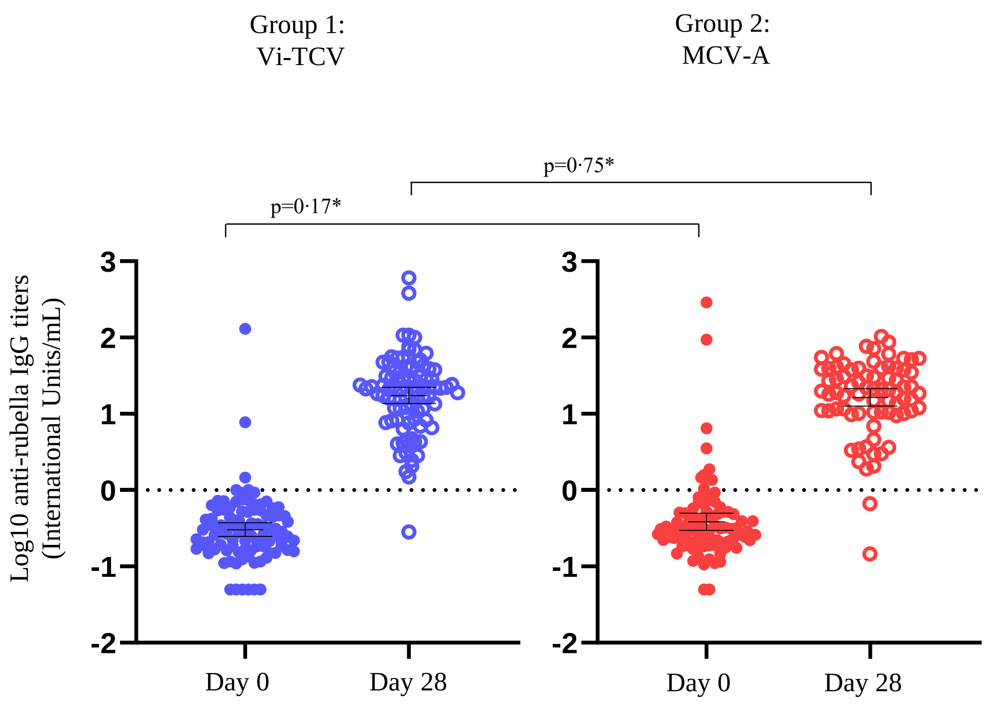

Supplemental Figure A1c Anti-rubella antibody titres before vaccination (day 0) and 28 days after vaccination

Blue and red shapes represent the antibody titre result for each individual participant in each corresponding vaccine group. Bar in the middle of individual participant results for each day represents the mean antibody titre value with 95% confidence interval.

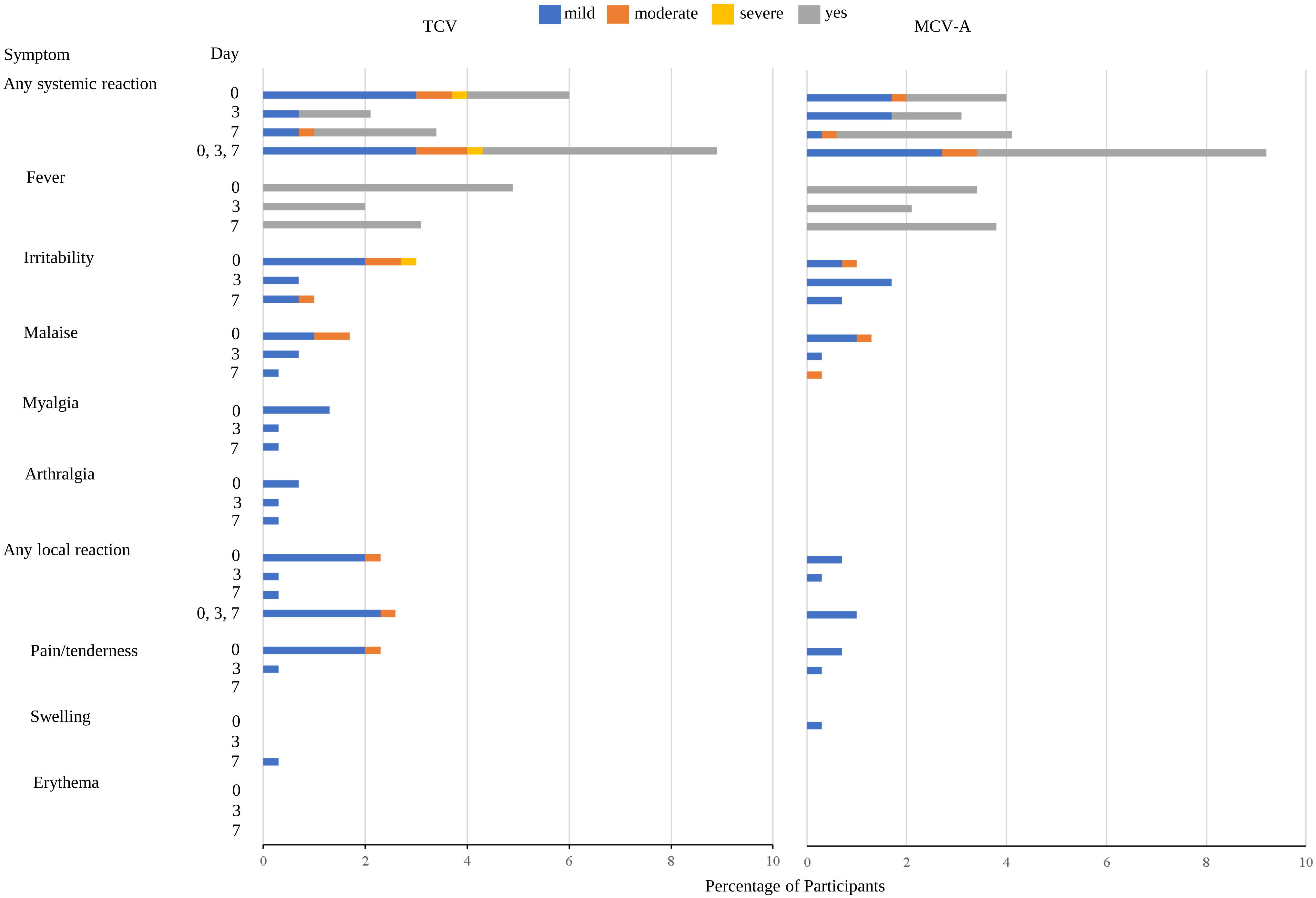
mild
moderate
severe
yes
TCV
MCV-A
Symptom
Day
Any systemic reaction
0
3
7
0, 3, 7
Fever
Irritability
Malaise
Myalgia
Arthralgia
Any local reaction
Pain/tenderness
Swelling
Erythema
0
2
4
6
8
10
Percentage of Participants

A Phase III Randomized, Double-Blind, Controlled Trial of the Clinical Efficacy of Typhoid Conjugate Vaccine (Vi-TCV) among Children Age 9 Months through 12 Years in Blantyre, Malawi

Protocol Number: NHSRC:17/07/1866

Sponsor: Center for Vaccine Development (CVD), University of Maryland School of Medicine, Baltimore, MD, USA

Funding Agency: Center for Vaccine Development (CVD), University of Maryland School of Medicine, Baltimore, MD, USA

Medical Investigator Responsible for Participants: Melita Gordon, M.D., Malawi Liverpool Wellcome Trust Clinical Research Programme, Institute for Infection and Global Health, University of Liverpool, UK

Sponsor Investigator: Kathleen Neuzil, M.D.

Co-Investigators: Karen Kotloff, M.D., Matthew Laurens, M.D., Robert Heyderman, MBBS., Chisomo Msefula, PhD, Tonney Nyirenda, PhD, James Meiring, MBChB.

Version Number: 14.0

Date: 21 Jun 2021

Statement of Compliance

This study will be carried out in accordance with the protocol, the International Conference on Harmonization Good Clinical Practice E6 (ICH-GCP); U.S. Code of Federal Regulations (CFR) applicable to clinical studies (45 CFR 46 and 21 CFR including parts 50 and 56 concerning informed consent and IRB regulations); and completion of Human Subjects Protection Training.

The study informed consent documents will embody the elements of consent as described in the Declaration of Helsinki and International Conference on Harmonization Good Clinical Practice. Compliance with these standards provides public assurance that the rights, safety and well-being of study subjects are protected, consistent with the principles that have their origin in the Declaration of Helsinki.

All key personnel (individuals responsible for the design and conduct of this study) will have completed Human Subjects Protection Training before interaction with any participants or to having access to their confidential study data.

SIGNATURE PAGE

The signatures below constitute the approval of this protocol and all attachments and provide the necessary assurances that this trial will be conducted according to all stipulations of the protocol, including all statements regarding confidentiality, and according to local legal and regulatory requirements and applicable guidelines.

Investigator Responsible for Participants:

Signed: Date:

_______________________________________ _______________

Melita Gordon, MD
Medical Officer, MLW

Sponsor Investigator:

Signed: Date:

_______________________________________ _______________

Kathleen Neuzil, MD
Medical Officer, CVD

TABLE OF CONTENTS

Statement of Compliance ii
TABLE OF CONTENTS iv
LIST OF ABBREVIATIONS ix
Protocol Summary xi
1. Key Roles 1
2. Background Information and Scientific Rationale 4
2.1. Background and Significance 4
2.1.1. Introduction and Literature Review 4
2.1.2. Burden of Typhoid Fever 5
2.1.3. Prevention of Typhoid Fever with Vaccines 6
2.2. Rationale 7
2.2.1. Rationale for Study Site 7
2.2.2. Rationale for Study Vaccine 8
2.2.3. Rationale for Control Vaccine 8
2.3. Potential Risks and Benefits 8
2.3.1. Potential Risks 8
2.3.2. Known Potential Benefits 10
3. Objectives 11
3.1. Study Objectives 11
3.1.1. Primary Objective 11
3.1.2. Secondary Objectives 11
3.1.3. Exploratory Objectives 12
3.2. Study Outcome Measures 13
3.2.1. Primary Outcome Measure 13
3.2.2. Secondary Outcome Measures 13
4. Overview of Study Design 14
4.1. Efficacy Study 14
4.2. Immunogenicity and Reactogenicity Substudy 14

4.3. Antibody Kinetics Substudy ... 15
4.4. HIV-exposed Vaccine Immunogenicity and Reactogenicity Substudy ... 16
4.5. Human Genetics ... 17
5. Enrollment and Withdrawal ... 17
5.1. Subjects ... 17
5.1.1. Inclusion Criteria ... 18
5.1.2. Additional inclusion criteria for HIV-exposed vaccine safety and immunogenicity substudy ... 18
5.1.3. Exclusion Criteria ... 18
5.1.4. Temporary Exclusion Criteria ... 18
5.1.5. Additional Exclusion Criteria for Safety and Immunogenicity Substudy ... 19
5.1.6. Additional Exclusion Criteria for HIV-exposed Vaccine Safety and Immunogenicity Substudy ... 19
5.2. Treatment Assignment Procedures ... 20
5.2.1. Randomization ... 20
5.2.2. Masking ... 21
5.2.3. Adherence to Randomization ... 21
5.3. Subject Withdrawal ... 21
5.3.1. Reasons for Withdrawal ... 21
5.3.2. Handling of Withdrawals ... 22
5.4. Termination of the Trial ... 22
5.4.1. Termination According to the Protocol ... 22
5.4.2. Suspension and/or Premature Termination ... 22
6. Study Vaccines ... 23
6.1. Vaccine Descriptions ... 23
6.1.1. Typbar-TCV™ ... 23
6.1.2. MenAfriVac Meningococcal Group A Conjugate Vaccine ... 23
6.1.3. Acquisition of Study Vaccines ... 23
6.1.4. Packaging and Labelling ... 24
6.1.5. Storage and Stability ... 24
6.2. Dosage, Preparation, and Administration of Study Vaccines ... 24

6.2.1. **Dosage** 24
6.2.2. **Preparation** 24
6.2.3. **Administration** 24
6.3. **Accountability procedures for the Study Vaccines** 25
6.4. **Concomitant Medications and Vaccinations** 25
7. **Study Schedule** 25
7.1. **Recruitment and Screening** 25
7.2. **Vaccination** 26
7.2.1. **Enrolment/Vaccination (Day 0)** 26
7.2.2. **Post-Vaccination Monitoring** 26
7.3. **Surveillance** 26
7.4. **Immunogenicity and Reactogenicity Substudy** 28
7.4.1. **Enrolment/Vaccination** 28
7.4.2. **Active Follow-Up** 28
7.5. **HIV-exposed Vaccine Safety and Immunogenicity Substudy** 28
7.5.1. **Enrolment/Vaccination** 28
7.5.2. **Active Follow-Up** 29
8. **Study Evaluations** 29
8.1. **Clinical Evaluations** 29
8.1.1. **Clinical Case Definitions** 29
8.2. **Laboratory Evaluations** 31
8.2.1. **Clinical Laboratory Evaluations** 31
8.2.2. **Blood Specimens** 31
8.2.2.1. **Collection of Clinical Specimens** 31
8.2.2.2. **Conditions for Handling, Transport and Storage of Clinical Specimens** 32
8.2.3. **Assays** 32
8.2.3.1. **Anti-Vi IgG Antibody ELISA** 32
8.2.3.2. **Measles PRN Assay** 32
8.2.3.3. **Measles ELISA** 32
8.2.3.4. **Rubella PRN Assay** 32

8.2.3.5. Rubella ELISA 32
8.2.3.6. HIV testing 33
9. Assessment of Safety 33
9.1. Methods and Timing for Assessing Safety Parameters 33
9.1.1. Post-Vaccination 33
9.1.2. Note on Timing 33
9.1.3. Definition of Adverse Events 33
9.2. Reporting Procedures 34
9.2.1. Adverse Events 34
9.2.2. Serious Adverse Events 34
9.3. Data and Safety Monitoring Board 34
10. Monitoring 35
10.1. Monitoring Plan 35
10.1.1. Site Initiation Visit 35
10.1.2. Follow Up Visits 35
10.1.3. Close-Out Visit 35
10.2. Inspections 36
10.3. Archiving 36
11. Statistical Considerations 36
11.1. Study Hypotheses 36
11.1.1. Primary Study Hypothesis 36
11.1.2. Secondary Study Hypotheses 36
11.2. Definition of Analysis Sets 37
11.2.1. Intention-to-Treat Population 37
11.2.2. Per-Protocol Population 37
11.3. Analysis Plan 37
11.3.1. Efficacy 37
11.3.2. Safety 38
11.3.3. Additional outcomes 39
11.4. Sample Size and Power Considerations 39

12. Quality Control and Quality Assurance 44
12.1. General 44
12.2. Trainings 44
13. Ethics and Human Subjects Protections 44
13.1. Ethics Standards 44
13.2. Institutional Review Board(s) 44
13.3. Recruitment 45
13.4. Informed Consent Process 45
13.5. Subject Confidentiality 45
13.6. Study Discontinuation 45
13.7. Sharing Study Results 45
13.8. Use of Stored Specimens 46
13.9. Compensation 46
14. Data Handling and Record Keeping 46
14.1. Source Data and Access to Data 46
14.2. Data Recording and Recordkeeping 46
14.3. Procedures for Reporting Protocol Deviations 47
15. Publication Policy 47
16. Literature References 47

LIST OF ABBREVIATIONS

AE	Adverse Event
BMGF	Bill and Melinda Gates Foundation
CFR	U.S. Code of Federal Regulations
CI	Confidence Interval
CRF	Case Report Form
CVD	Center for Vaccine Development
DSMB	Data and Safety Monitoring Board
˚C	Degrees Celsius
EDTA	Ethylenediaminetetraacetic acid
ELISA	Enzyme-Linked Immunosorbent Assay
EPI	Expanded Programme on Immunization
GCP	Good Clinical Practice
GMC	Geometric Mean Concentration
GMT	Geometric Mean Titer
HSA	Health Surveillance Assistant
ICF	Informed Consent Form
ICH	International Conference on Harmonisation
IDR	Incidence Density Ratio
IgA	Immunoglobulin A
IgG	Immunoglobulin G
IgM	Immunoglobulin M
IND	Investigational New Device
iNTS	Invasive Nontyphoidal *Salmonella*
IRB	Institutional Review Board
ISM	Independent Safety Monitor
LPS	Lipopolysaccharide
MCV-A	Meningococcal A Conjugate Vaccine
mIU/mL	Milli-International Units per Milliliter
MUAC	Middle Upper Arm Circumference
Mg	Micrograms
Mg	Milligrams
mL	Milliliters
MLW	Malawi Liverpool Wellcome Trust Clinical Research Programme
NHSRC	National Health Science Research Committee
NIH	National Institutes of Health
PI	Principal Investigator
PMPB	Pharmacy, Medicines and Poisons Board
PRN	Plaque Reduction Neutralization titers (for measles)
PS	Polysaccharide
QECH	Queen Elizabeth Central Hospital
S. Typhi	*Salmonella enterica* serovar Typhi
SAE	Serious Adverse Event

SMP	Site Monitoring Plan
STRATAA	Strategic Alliance on Typhoid across Africa and Asia
SUSAR	Suspected Unexpected Serious Adverse Reactions
TCV	Typhoid Conjugate Vaccine
TSAP	Typhoid Surveillance in Africa Program
TyVAC	Typhoid Vaccine Acceleration Consortium
VE	Vaccine Efficacy
Vi	Virulence
Vi-PS	Vi-Polysaccharide
Vi-rEPA	Vi conjugated to exoprotein A from *Pseudomonas aeruginosa*
Vi-TCV	Vi-Typhoid Conjugate Vaccine

PROTOCOL SUMMARY

**Title:**	A Randomized, Double-Blind, Controlled Trial of the Clinical Efficacy of Typhoid Conjugate Vaccine (Vi-TCV) among Children Age 9 Months through 12 Years in Blantyre, Malawi
**Phase:**	III
**Vaccinated Population:**	Up to 28,500 healthy children 9 months through 12 years of age in Blantyre, Malawi.
**Number of Sites:**	Study is multi-center – Blantyre and Thyolo districts
**Study Duration:**	Approximately 48 months.
**Participation Duration:**	The study will continue until all children have been followed for a minimum of 36 months after vaccination AND a minimum of 30 cases that meet the primary outcome definition (symptomatic, blood-culture confirmed *S*. Typhi infection) - occur. Each participant will be enrolled for approximately 39-48 months, depending on when vaccination occurs and when the case total is reached, and to allow for an extended efficacy follow-up period as an exploratory analysis. Participants in the HIV-exposed substudy will be enrolled for approximately 7- 12 months.
**Description of Agent or Intervention:**	One 0.5 mL dose of either Typhoid Conjugate Vaccine (Vi-TCV) (Typbar-TCV™, Bharat Biotech, Hyderabad, India) *or* Meningococcal A Conjugate Vaccine (MCV-A) (MenAfriVac®, Serum Institute of India, Limited, Pune, India). Participants in the HIV-exposed substudy will receive either one dose of TCV at 9 months of age, one dose of TCV at 15 months of age, or 2 doses of TCV at 9 and 15 months of age.
**Objectives:**	**Primary Objective:** To determine the efficacy of Vi-TCV in reducing rates of symptomatic, blood culture-confirmed *S.* Typhi infection among children who receive Vi-TCV compared to children who receive MCV-A<br><br>Efficacy will be evaluated by comparing the incidence rate of symptomatic, blood culture-confirmed *S*. Typhi infection meeting specimen collection criteria among children who

Objectives, cont.:

receive Vi-TCV compared to children who receive MCV-A during the study period. The specimen collection criteria will be defined as positive blood culture for *S.* Typhi in association with:

1. Subjective fever for ≥72 hours OR
2. Objective fever ≥38 ˚C axillary OR
3. Hospitalization with history of fever of any duration

Vaccine efficacy (VE) will be calculated as one minus the relative rate of symptomatic typhoid fever in the Vi-TCV group compared to that in the MCV-A group. Participants in the HIV-exposed substudy will not be assessed for vaccine efficacy.

Secondary Objectives:

1. 1. To determine the safety profile of vaccination with Vi-TCV or MCV-A.

The safety profile of Vi-TCV will be measured by comparing the proportions of participants who experience solicited and unsolicited local and systemic reactions among children who receive Vi-TCV and those who receive MCV-A in the following categories:

a. Immediate reactions that occur within 30 minutes of administration of study vaccine, observed and evaluated by study staff or reported by the subject.
b. Local and systemic solicited and unsolicited events that occur within 7 days of receipt of study vaccine in a subset of participants, observed by study staff or reported by the subject's parent/guardian to study staff.
c. Other non-serious adverse events that occur within 28 days of receipt of study vaccine in a subset of participants, observed by study staff or reported by the subject's parent/guardian to study staff.
d. Serious adverse events that occur within six months of receipt of study vaccine that are observed by study staff or reported by the subject's parent/guardian to study staff.

Objectives, cont.:

2. To determine the immunogenicity of Vi-TCV in a subset of participants, by age group and HIV status, as measured by serum anti-Vi IgG antibodies (percent seroconversion and Geometric Mean Titer (GMT)) at approximately 28 days following vaccination.
3. To determine the number of blood culture-confirmed cases of typhoid fever prevented during the study period by comparing the incidence of blood culture-confirmed typhoid fever in participants receiving Vi-TCV to the incidence in participants receiving MCV-A. Participants in the HIV-exposed substudy will not be assessed for blood culture-confirmed typhoid fever.

Exploratory Objectives (excluding HIV-exposed substudy participants, except as noted below**):**

1. To determine the effect of Vi-TCV on hospitalizations due to blood culture-confirmed typhoid fever and the number of hospitalizations due to typhoid fever prevented by Vi-TCV.
2. To determine the effect of Vi-TCV on the duration of hospitalization for febrile illness during the study period.
3. To determine the effect of Vi-TCV for preventing all-cause hospitalizations and the number of hospitalizations prevented during the study period.
4. To describe the clinical characteristics of blood culture-confirmed typhoid fever in study participants, including the percentage of participants with specific signs and symptoms.
5. To determine the effect of Vi-TCV on outpatient visits for fever and the number of visits prevented during the study period.
6. To determine the effect of Vi-TCV on number of outpatient and hospitalized cases of clinically diagnosed typhoid fever.
7. To determine the effect of Vi-TCV on hospitalizations for febrile illness and the number of hospitalizations prevented during the study period.

Objectives, cont.:

8. To determine the effect of Vi-TCV on antibiotic usage and the number of antibiotic courses and days of antibiotic use prevented during the study period.
9. To determine the effect of Vi-TCV on all-cause mortality and the number of deaths prevented during the study period.
10. To compare the number of episodes of illness for which blood cultures are collected during the study period between Vi-TCV and MCV-A groups.
11. To compare the incidence of hospitalizations for meningitis between Vi-TCV and MCV-A groups.
12. To evaluate the rate and recurrence of ileal perforations secondary to typhoid fever.
13. To determine the effect of Vi-TCV on the incidence of complications of typhoid fever (e.g., perforations, acute abdominal procedures, death) and the number of complications prevented during the study period.
14. To determine the persistence of serum anti-Vi IgG antibodies in a subset of participants, by age group, at approximately 2-3 years following vaccination.
15. To evaluate the efficacy and above outcomes by age groups.
16. To compare the anti-measles IgG seroprotection and Geometric Mean Concentrations (GMCs), plaque reduction neutralization (PRN) titers, among children receiving Vi-TCV + measles-rubella vaccine, and children receiving MCV-A + measles-rubella vaccine, including children enrolled in the HIV-exposed substudy.
17. To compare the anti-rubella IgG seroprotection and GMCs among children receiving Vi-TCV + measles-rubella vaccine and children receiving MCV-A + measles-rubella vaccine, including children enrolled in the HIV-exposed substudy.
18. To determine the immunogenicity of Vi-TCV in a subset of participants, by age group and HIV status, as measured by assays of cellular and antibody functional immunity at approximately

Objectives, cont.:

28 days following vaccination, including children in both immunogenicity substudies.

19. To evaluate the relationship between serum anti-Vi IgG at 28 days post-vaccination and the development of symptomatic, blood culture-confirmed *S.* Typhi infection among children in the immunogenicity subset.
20. To quantify typhoid-specific immune responses among a subset of children with blood culture confirmed S. Typhi infection and compare between children receiving Vi-TCV and children receiving MCV-A.
21. To determine the rate of serum anti-Vi IgG antibody decay over the study period among a subset of children meeting blood-culture-collection criteria.
22. To study serological exposure over the study period to Invasive Nontyphoidal *Salmonella* (iNTS) and novel Coronavirus SARS-CoV-2 (COVID-19), among a subset of children meeting blood-culture-collection criteria.
23. To assess host factors affecting acquisition of enteric fever through the identification of human genes that either increase or decrease the risk of getting typhoid and understanding how these gene variants affect biological function.
24. To determine the number of blood culture-confirmed cases of typhoid fever by study site and by high and low incidence settings.
25. To determine the extended efficacy of Vi-TCV in reducing rates of symptomatic, blood culture-confirmed S. Typhi infection among children who receive Vi-TCV compared to children who receive MCV-A

Description of Study Design:

This study is a double-blind, individually randomized, controlled, clinical efficacy trial with two vaccine groups: Vi-TCV and MCV-A. Participants (up to 28,500) will be randomized in a 1:1 ratio. Children 9 months through 12 years of age in the Blantyre area who meet the inclusion criteria will be eligible for enrollment.

Participants will be unaware of which study vaccine, Vi-TCV or MCV-A, is received. A subset of children will have home or

clinic visits on days 3, 7, 28 and 180 post-vaccination to assess select solicited events, unsolicited events, and all serious adverse events. SAEs in all participants will be monitored through the end of the trial.

An additional subset of up to 325 HIV-exposed and unexposed children aged 9-12 months enrolled in Blantyre and Thyolo will receive one or two doses of TCV vaccine and have phone or clinic visits 7 days after each vaccination to assess solicited events, unsolicited events, and all serious adverse events up to 7 months after first vaccinations. This subset will also have clinic visits 28 days after each vaccination to assess immunogenicity.

For the evaluation of efficacy, passive surveillance will be conducted for up to 48 months to identify cases among vaccinated subjects. Children who meet the protocol-defined specimen collection criteria will have a blood culture collected. Additional information will be collected from any child who has a blood culture obtained. This will include information about the signs and symptoms of the illness and treatment given. Likewise, any child with blood culture-confirmed typhoid fever will have follow-up until the illness resolves. Additional information on the illness, treatment and complications will be recorded. Vaccine efficacy will be evaluated based on cases accrued at three timepoints: on through April 3, 2020 when all participants have been followed for a minimum of 18 months; through September 30, 2021, the end date for the full analysis of all subgroups, when all participants have been followed for a minimum of 36 months; and until the end of the extended surveillance period, through December 31, 2021.

A subset of 600 children (200 in each of three age groups: 9-11 months, 1-5 years, and 6-12 years) will be included in an additional **Vaccine Immunogenicity and Reactogenicity Substudy**. More stringent exclusion criteria will apply for this subset. The purpose of this detailed evaluation is to assess the reactogenicity of the vaccine and the immune responses to Vi-TCV. Serum specimens will be collected on day 0 (before vaccination) and on post-vaccination days 28 and between days 730 and day 1095 from all children included in the sub-study. For the children in the 9-11 month group, Vi-TCV or

MCV-A will be administered with measles-rubella-containing vaccine, as per Malawi Expanded Programme on Immunization (EPI) schedule. These 9-11-month-old children will have antibody to measles and rubella assessed on days 0 and 28. All children in the sub-study will be assessed at days 3 and 7 following vaccination for solicitation of local and systemic adverse events. Serious and non-serious adverse events will be assessed at days 28 and 180.

An additional subset of -up to 225 HIV-exposed-but-uninfected and up to 100 HIV-unexposed children ages 9-11 months will be included in an additional **HIV-exposed Vaccine Immunogenicity and Reactogenicity Substudy**. The purpose of this detailed evaluation is to assess the reactogenicity of the vaccine and the immune responses to one or two doses of Vi-TCV in HIV-exposed children. Up to 225 HIV-exposed children in this substudy will be randomized 1:1:1 to receive either TCV at 9-11 months and TCV at 15 months (Group 1), TCV at 9-11 months only (Group 2), or TCV at 15 months only (Group 3). A separate group of about 100 HIV-unexposed children will serve as controls and receive TCV at 9-11 months and TCV at 15 months (Group 4). Serum specimens will be collected on day 0 (before vaccination) and on 28 days after each vaccination from all children included in the sub-study. For this substudy, Vi-TCV will be administered with measles-rubella-containing vaccine #1 at 9-11 months and/or #2 at 15 months, as per Malawi EPI schedule. Due to the impact of the prolonged interruption of surveillance activities caused by COVID-19, the Vi-TCV dose that was to be administered to some participants at 15 months was not administered. Participants who had not received any Vi-TCV will be provided with an opportunity to receive it once study activities are safe to resume. These 9-11-month-old children will have antibody to typhoid, measles, and rubella assessed on day 0 and 28 days after each vaccination. All children in the sub-study will be assessed at 7 days after each vaccination for solicitation of local and systemic adverse events. Serious and non-serious adverse events will be assessed up to the last study visit for HIV-exposed substudy participants. This substudy will run until March 30, 2022.

**Inclusion and Exclusion Criteria:**<br><br>**Inclusion and Exclusion Criteria, cont.**	**Inclusion Criteria:** Each subject receiving study vaccine (Vi-TCV or MCV-A) must satisfy the following inclusion criteria at study entry:<br>• Male or female child at least 9 months of age, in good health[1], and no older than 12 years and 364 days of age at the time of study vaccination.<br>• A child whose parent or guardian resides primarily within the Ndirande or Zingwangwa study areas at the time of study vaccinations and who intends to be present in the area for the duration of the trial.<br>• A child whose parent or guardian has voluntarily given informed consent.<br>• Additionally, for the HIV-exposed substudy, HIV-exposed children whose parents or guardians reside primarily within Bvumbwe study area at the time of study vaccinations and who intend to be present in the area for the duration of the trial<br><br>**Exclusion Criteria:** No subject receiving study vaccine (Vi-TCV or MCV-A) may have any of the following exclusion criteria at study entry:<br>• History of documented hypersensitivity to any component of the vaccine.<br>• Prior receipt of any typhoid vaccine in the past 3 years.<br>• History of severe allergic reaction with generalized urticarial, angioedema, or anaphylaxis.<br>• Any condition determined by the investigator likely to interfere with evaluation of the vaccine or to be a significant potential health risk to the child or make it unlikely that the child would complete the study.<br><br>The following will be considered temporary contraindications to enrollment and vaccination. If these apply, the participant will be temporarily excluded for vaccination until 48 hours has passed. A re-assessment will be needed to ensure these temporary exclusion criteria no longer exist:<br>• Reported fever within 24 hours prior to vaccination<br>• Use of anti-pyretics within 4 hours prior to vaccination

[1] *As determined by medical history to evaluate acute or currently ongoing chronic medical diagnoses or conditions. Chronic medical diagnoses or conditions, including HIV infection, should be stable for the last 60 days.*

An additional temporary exclusion criterion will be:

- Receipt of measles-rubella vaccine in the one month prior to enrollment, as determined by parental history or vaccination card.

Estimated Time to Complete Enrollment:

Full enrollment is anticipated over 8 months

Statistical Analyses:

The primary study hypothesis is that Vi-TCV is significantly efficacious in reducing rates of symptomatic, blood culture-confirmed *S.* Typhi infection, meeting the case definition among children receiving Vi-TCV as compared to children receiving MCV-A.

Data analysis will be conducted by the Center for Vaccine Development, University of Maryland School of Medicine, in Baltimore, MD. The statistician will remain blinded until the Statistical Analysis Plan has been finalized and the database has been frozen. The Sponsor and investigators will participate in the review and approval of the Statistical Analysis Plan. The efficacy analysis will be performed on an intention-to-treat basis. Supportive per-protocol analyses will also be conducted which include all cases meeting study outcome definitions and occurring more than 14 days after receipt of study vaccine.

Vaccine efficacy (VE) is defined as the reduction in incidence rates of first episodes of culture-confirmed typhoid fever:
Two sets of vaccine efficacy (VE) analyses will be conducted. Due to the impact of the prolonged interruption of surveillance activities caused by COVID-19, the first set of VE analyses will use data collected through April 3, 2020 by which date participants had been followed for a minimum of 18 months and the results will be treated as the primary results. The second set of VE analyses will use data collected through September 2021 and will be used to evaluate the duration of vaccine protection. Subgroups analyses will be conducted to calculate and compare VE by age group (<2 , 2-<5 and 5+) and (<5 vs. 5+). If there are a minimum of 30 symptomatic, blood-culture confirmed *S*. Typhi infection cases in each age subgroup, then this will be done for the 18-month analysis

(through April 3, 2020). surveillance endpoint. If not, this age group analysis will be done at the end of the September 2021 surveillance period, regardless of number of cases in each age group. An extended analysis of efficacy will include all cases through December 2021.

Statistical Analyses, cont.:

In addition, VE will be computed 1 to 48 months after vaccination using the life table method.

We assume the number of first episodes of culture-confirmed typhoid fever follows a Poisson distribution in each of the Vi-TCV and MCV-A groups and define x_1 and x_2 as the numbers of first episodes in recipients of Vi-TCV and MCV-A vaccines, respectively. Then the number of first episodes in Vi-TCV recipients, conditional on the total first episodes $X = x_1 + x_2$, follows a binomial distribution with parameters X and $P = x_1 / (x_1 + x_2)$. Now letting h = (total follow-up time among MCV-A recipients) / (total follow-up time among Vi-TCV recipients), we have

$$VE = (1 – R) \times 100\%, \text{ where } R = hP / (1-P).$$

Then we can test the null hypothesis that the vaccine has no protective efficacy, H_0: VE ≤ 0, by testing the equivalent hypothesis that $P \geq P_0$, where $P_0 = 1 / (h+1)$; we expect h to be approximately 1 and P_0 to be approximately 0.5.

A two-sided 95% confidence interval (CI) for VE is calculated by first obtaining an exact two-sided 95% CI for P and then transforming the limits of that interval, using the relationship

$$VE = 1 – hP / (1-P) \times 100\%.$$

The null hypothesis can be tested by an exact one-sided p-value; $p < 0.025$ will indicate statistically significant efficacy. Equivalently, significant efficacy will be indicated by a lower limit > 0 for the 95% CI for VE. With assumption of 80% vaccine efficacy, the minimum number of cases needed to evaluate the primary outcome with 90% power is 30. With 28,500 children enrolled, this is likely to be achieved with an average of 24 months follow-up per participant. Lower efficacy, fewer children enrolled, or a lower than assumed incidence will require longer follow-up to reach the minimum number of cases.

Table 1. Schedule of Events

**Study Visit Type**	**Recruitment**	**Screening**	**Eligibility, enrollment, vaccination**	**Follow up©**	**Follow up©11**	**Follow up©11**	**Follow up©, vaccination #2 11**	**Follow up 11**	**Follow up 11**	**Follow up©12**	**Early termination**
**Study Visit Number**		**V0 0**	**V0 1**	**V0 2**	**V0 3**	**V0 4**	**V0 5**	**V0 5a**	**V0 5b**	**V0 6**	
**Study Time point**			**D0**	**D3**	**D7**	**D28**	**D180**	**D187**	**D208**	**D730 to D1095**	
Study introduction, recruiting	X	X[2]	X[2]								
Obtain informed consent		X	X[23]								
Review eligibility criteria		X	X[24]								
Review medical history		X	X[24]	X[4]	X[4]	X[4]	X[4]	X[4]	X[4]	X[4]	X[4]
Review concomitant meds		X	X[24]	X[4]	X[4]	X[4]	X[4]	X[4]	X[4]	X[4]	X[4]
Review vaccination history		X	X[24]	X[4]	X[4]	X[4]		X[4]	X[4]		X[4]
Axillary temperature		X	X[56]			X[6]	X[11]		X[6]	X[6]	X[6]
Height and weight and Middle Upper Arm Circumference (MUAC)[″]		X	X[5]							X	X
Targeted physical exam		X	X[245˚]			X[4˚]			X[4]	X[4˚]	X[4˚]
Vaccination			X								
30-min post-vaccination evaluation			X[Ω]								
Post-vaccination reactogenicity assessment[^]				X	X					X	X
Venous blood collection for immunogenicity ^			X[57]			X[7]			X[7]	X[7]	X[7]
Assess and record SAEs			X[8]	X	X	X	X	X	X		X[9]
Assess and record solicited AEs			X[8]	X	X			X[11]			X[10]

© This visit will occur in the immunogenicity and reactogenicity substudy.
[2] If not performed at previous visit.
[3] Confirm informed consent for study procedures.
[4] Review/confirm information or activity in participants previously consented and screened.
[5] Prior to study vaccination and used as a baseline.
[6] Participants must not eat or drink anything hot or cold or smoke within 10 minutes prior to taking oral temperature.
″ Height and weight and MUAC will only be measured for children in the immunogenicity and reactogenicity substudy who are younger than 5 years at time of enrollment.
˚ Targeted physical exam if indicated based on interim medical history.
Ω Only in immunogenicity and reactogenicity substudy.
^ Reactogenicity and immunogenicity will be studied in the same subset of children (N=600).
[7] 5ml of blood collected for immunogenicity and measles-rubella-noninterference testing
[8] If AE/SAE occurs post-vaccination
[9] Only if visit occurs within 6 months after study vaccination.
[10] Only if visit occurs within 7 days after study vaccination.
[11] This visit will occur in the HIV-exposed substudy.
[12]. This visit will occur up to day 1095 for participants who could not make the day 730 visit due to the COVID-19 interruption.

1. Key Roles

Individuals

Sponsor Investigator:

Kathleen Neuzil, M.D.
Center for Vaccine Development (CVD)
University of Maryland School of Medicine
685 West Baltimore St, Rm 480
Baltimore, MD 21201
Phone: +1 410 706-3502
Fax: +1 410 706-6205
Email: kneuzil@som.umaryland.edu

Investigator Responsible for Participants:

Melita Gordon, M.D.
Malawi Liverpool Wellcome Trust Clinical Research Programme, Queen Elizabeth Central Hospital
College of Medicine
P.O. Box 30096
Chichiri, Blantyre 3, Malawi
Phone: +265 1874628
Email: magordon@liverpool.ac.uk

Other investigators:

Robert Heyderman, M.D.
University College London
Division of Infection & Immunity
90 Gower Street
London, WC1E 6BT, UK
Phone: +44 20 3108 2133
Email: Robert.Heyderman@uclh.nhs.uk

Chisomo Msefula, PhD
University of Malawi, College of Malawi
Private Bag 360
Chichiri, Blantyre 3, Malawi
Phone: +265 1871911
Email: cmsefula@mlw.mw

Tonney Nyirenda, PhD
University of Malawi, College of Malawi
Private Bag 360
Chichiri, Blantyre 3, Malawi
Phone: +265 1871911
Email: tnyirenda@mlw.mw

James Meiring, M.D.
Malawi Liverpool Wellcome Trust Clinical Research
Programme, Queen Elizabeth Central Hospital
College of Medicine
P.O. Box 30096
Chichiri, Blantyre 3, Malawi
Phone: +265 1874628
Email: james.meiring@paediatrics.ox.ac.uk

Karen Kotloff, M.D.
CVD, University of Maryland School of Medicine
685 West Baltimore St, Rm 480
Baltimore, MD 21201
Phone: +1 410 706-5328
Fax: +1 410 706-6205
Email: kkotloff@som.umaryland.edu

Matthew Laurens, M.D.
CVD, University of Maryland School of Medicine
685 West Baltimore St, Rm 480
Baltimore, MD 21201
Phone: +1 410 706-5328
Fax: +1 410 706-6205
Email: mlaurens@som.umaryland.edu

Institutions

Sponsor:

Center for Vaccine Development (CVD), University of
Maryland School of Medicine
685 West Baltimore St, Rm 480
Baltimore, MD 21201
Contact: Kathleen Neuzil, MD
Phone: +1 410 706-3502
Fax: +1 410 706-6205

Email: kneuzil@som.umaryland.edu

Funding Agency:

Center for Vaccine Development (CVD), University of Maryland School of Medicine
685 West Baltimore St, Rm 480
Baltimore, MD 21201

Institutional Review Boards:

University of Maryland Institutional Review Board (IRB)
620 W. Lexington St
Baltimore, MD 21201
phone: +1 410 706-5037
email: hrpo@umaryland.edu

Pharmacy, Medicines & Poisons Board (PMPB)
PO Box 30241
Off Chilambula Road, Capital City
Lilongwe 3
Malawi
Tel: +265 1 750 108/+265 1 755 165/+265 1 755 166
Fax: +265 1 755204
admin@pmpb.malawi.net

National Health Science Research Committee (NHSRC)
PO Box 30O377
Lilongwe 3
Malawi
Phone: +26 560 1726422
Email: directorgeneral@ncst.mw

Research Ethics Sub-Committee for Physical Interventions
University of Liverpool
Foundation Building
Liverpool L69 7ZX
United Kingdom
Phone: +44 151 794 8290
Email: ethics@liverpool.ac.uk

2. Background Information and Scientific Rationale

2.1. Background and Significance

2.1.1. Introduction and Literature Review

Typhoid fever is an acute infection with *Salmonella enterica* serovar Typhi (*S.* Typhi) acquired through exposure to food or water contaminated with human feces. Humans are the sole reservoir of *S*. Typhi. In endemic areas, the incidence of typhoid fever has traditionally been highest in school-age children (5-19 years), and is being increasingly recognized in the under 5 years age group, based on passive surveillance of patients who seek health care. In countries with inadequate sanitation and water contamination, infections may be acquired from water and food contaminated with human waste. In addition, typhoid may be transmitted via a food vehicle handled by an individual who is a chronic carrier of *S*. Typhi.

An estimated 22 million cases of typhoid fever and 200,000 related deaths occur worldwide each year. Vaccine field trials in Latin America, Asia, and South Africa found incidence rates ranging from 200 to 800 cases per 100,000 persons. Areas with an incidence of >100/100,000 are considered as high endemic. However, it should be recognized that estimating typhoid fever incidence is challenging in the developing world due to the non-specific clinical presentation of the disease, which mimics many other common clinical syndromes (e.g. malaria), and poor access to reliable laboratory based diagnostics.

Typhoid fever may be asymptomatic or present clinically with mild to severe disease. Clinical disease includes high fever, headache, rash, abdominal pain, malaise, myalgia, and anorexia. Serious complications of typhoid fever include intestinal perforation and hemorrhage or neurological complications; this is primarily observed in those whose illness has been untreated for longer than 2 weeks. An estimated 2-5% of patients become chronic carriers of *S*. Typhi, with the bacteria colonizing the gall bladder of affected individuals with persistent shedding of the bacteria in the stool.

S. Typhi organisms express an immunogenic lipopolysaccharide (LPS) O antigen and flagellar H antigen. In addition, strains recovered from patients express a surface polysaccharide capsule designated the Vi (for virulence) antigen. In mice, Vi antigen enhances the pathogenicity of *S*. Typhi.[1] Measurement of the titer of serum Vi antibodies is useful for detection of chronic *S.* Typhi carriers. Vi antigen is also important because when given as a vaccine, Vi antigen stimulates a protective serum immune response.

2.1.2. Burden of Typhoid Fever

2.1.2.1. Typhoid Fever in Africa

The epidemiology of typhoid fever in Africa has been less well studied than in Asia, although the disease is endemic on both continents. The recent Typhoid Fever Surveillance in Africa Program (TSAP) measured the incidence of invasive *Salmonella* bloodstream infections across 13 sites in 10 countries in Africa over a 27-month period. [2] A major finding from these studies was that the overall adjusted incidence of typhoid fever was 2-3 times higher than previous estimates.[3] These investigators found the overall annual incidence was 10-100 cases/100,000 people comparable to those previously reported in Asia. However, there was marked heterogeneity across the continent and even within countries such as Ghana, Burkina Faso, and Kenya. Incidence was highest in the 2-14 year old children. Importantly, 47% of *S*. Typhi isolates were multidrug resistant.

In sub-Saharan Africa the annual mortality rate from typhoid fever is 3.3 per 100,000 people, accounting for 250 years of healthy life lost per 100,000 people. The mortality rate is highest for boys and girls age 1-4 years. Outbreaks of antibiotic-resistant typhoid fever have occurred throughout sub-Saharan Africa in recent years, including in Malawi, Uganda, Zimbabwe, Zambia, and the Democratic Republic of Congo.[4-8]

2.1.2.2. Typhoid Fever in Malawi

Starting in 1998 investigators at the Malawi Liverpool Wellcome Trust Clinical Research Programme (MLW) have systematically collected blood and cerebrospinal fluid for culture from febrile patients at the Queen Elizabeth Central Hospital (QECH) in Blantyre, Malawi. [9] Between 1998 and 2010, there were approximately 14 cases of *S*. Typhi per year with 6.8% demonstrating multi-drug resistance. A rapid increase in cases began in 2011, peaking in 2014 with 782 cases providing a minimum incidence estimate for Blantyre of 184/100,000 person-years of observation. These infections have resulted in a high mortality rate (2.5%) despite use of fluoroquinolone antibiotics. Much of this increase is likely due to the emergence and spread of the multidrug-resistant H58 haplotype of *S*. Typhi, with mathematical modelling suggesting the incidence will decline to a more stable endemic level. In Malawi, typhoid is isolated throughout the year but does have a seasonal pattern, peaking at the end of the wet season and during the early dry season. [10] Through the Strategic Typhoid Alliance across Africa and Asia (STRATAA) program, detailed epidemiology and transmission studies are currently ongoing and have provided estimates of disease burden in the populations that will serve as the site for this vaccine trial.

In addition to the high disease burden in Africa, high levels of resistance to multiple antibiotics limit the efficacy of treatment of cases. Since 2012, 90-100% of *S*. Typhi isolates in Blantyre, Malawi are multiply drug resistant. As the prevalence of antibiotic resistance increases, typhoid will become increasingly difficult or even impossible to treat. In the pre-antibiotic era, the case fatality rate was 15%.

2.1.3. Prevention of Typhoid Fever with Vaccines

2.1.3.1. Previous Vaccines

Two typhoid vaccines are pre-qualified by the World Health Organization (WHO) and have been widely available for the prevention of typhoid fever: a live oral vaccine strain Ty21a and a parenteral purified Vi polysaccharide (Vi-PS) vaccine. Each of these vaccines mediates protection by a distinct mechanism. Parenteral Vi-PS vaccine elicits serum Vi antibody, and a Vi antibody concentration $\geq$ 1.0 µg/ml has been proposed as a correlate of protection.[1] In contrast, Ty21a does not express Vi capsule and therefore does not elicit Vi antibodies. Rather, the protection conferred by Ty21a is believed to be mediated by serum and mucosal antibodies to other antigens of *S.* Typhi and by cell-mediated immune responses, including cytotoxic T lymphocytes.[11] A drawback of Ty21a is that 3-4 doses must be administered, it is not licensed in children younger than 6 years of age, and should not be used in pregnant women, immunocompromised persons, or those receiving antibiotics.

Vi antigen, which comprises the capsular polysaccharide of *S*. Typhi, is an essential virulence factor in the pathogenicity of *S*. Typhi. Purified Vi-PS vaccine mediates protection against typhoid fever by stimulating serum IgG which acts upon organisms that gain access to the bloodstream. Multiple efficacy trials in Asia, South Africa, and China have demonstrated the safety and immunogenicity of Vi vaccine.[12-14] Vi-PS vaccine is well tolerated and confers 65-70% protective efficacy, although immunity is short-lived and repeat vaccination is recommended every 2 years. Vi-PS vaccine is poorly immunogenic in children younger than 2 years of age and is not licensed for that age group.

Polysaccharide vaccines, such as those developed against *Neisseria meningitidis, Streptococcus pneumoniae, Haemophilus influenzae* type b (Hib), as well as *S.* Typhi, prevent infection by inducing an immune response against specific capsular polysaccharides. Like other pure polysaccharide vaccines, Vi polysaccharide vaccine does not induce immunologic memory and, as mentioned above, is poorly immunogenic in young children under 24 months of age. These deficiencies have been addressed by conjugating Vi to a carrier protein. A typhoid conjugate vaccine was developed by the US National Institutes for Health (US NIH) in 1994 utilizing Vi-polysaccharide conjugated with a recombinant exoprotein A from *Pseudomonas aeruginosa* (rEPA). A two-dose schedule six weeks apart was shown to be highly immunogenic with a protective efficacy of 91.1% in children aged 2 to 5 years in a trial in Vietnam. [9, 10] Fewer than 2% of children 2-5 years of age experienced adverse events (none serious). A second study demonstrated its immunogenicity in infants. [11] However, no trials were conducted to assess the level of protection induced by this vaccine in 6-23-month-olds. The licensure of Vi-rEPA has been delayed due to lack of regulatory precedent for the use of rEPA carrier based vaccines.

2.1.3.2. Typbar Typhoid Conjugate Vaccine

A typhoid Vi conjugate vaccine (Typbar-TCV™) has been developed by Bharat Biotech International, Hyderabad, India. This vaccine consists of 25 µg of Vi polysaccharide conjugated to a nontoxic tetanus toxoid protein carrier. Because the polysaccharide antigen is conjugated to an immunogenic protein, Vi-TCV stimulates a T cell-dependent response that results in immune responses in young children and immunologic memory. Vi-TCV elicits a stronger anti-Vi response than unconjugated Vi polysaccharide and the elicited antibodies have higher avidity than those detected after the unconjugated Vi vaccine.

The safety and immunogenicity of Vi-TCV has been studied in adults and children down to 6 months of age in India, a typhoid endemic area.[15] Vi-TCV given as a single dose via the intramuscular route was well tolerated and induced strong serum anti-Vi IgG responses in children at least 6 months of age and in adults.[15] A single dose of Vi-TCV elicited 4-fold seroconversion rates of 98.05%, 99.17% and 92.13% in subjects ≥6 months to 2 years, >2 to 15 years and >15 to 45 years respectively.[15]

In an adult volunteer challenge study at the University of Oxford, a single dose of Vi-TCV was well tolerated and induced 54.6% protective efficacy for the conjugate (and 52.0% efficacy for the Vi polysaccharide alone) at 28 days after vaccination. [16, 17]

In summary, this Vi conjugate vaccine is a promising candidate for the control of typhoid fever in Africa because of its one-dose schedule and its demonstrated immunogenicity and safety profile in children. To date, no studies of the field efficacy of typhoid conjugate vaccines in Africa have been conducted. This protocol is designed to test the efficacy of Vi-TCV in an endemic setting in Malawi.

2.2. Rationale

2.2.1. Rationale for Study Site

As above, prior typhoid conjugate vaccines have proven efficacy in Asian populations, and Vi-TCV has been shown to be safe, well-tolerated and immunogenic in Indian children. However, typhoid conjugate vaccines have not been tested in African children. While other conjugate vaccines (pneumococcal, Hib, meningitis) have performed favorably in children in Africa, data on typhoid conjugate vaccines are needed to inform decisions on vaccine introduction in Malawi and other Africa countries.

Recent data support an under recognized burden of typhoid fever in African countries, recent introductions to Africa of the multidrug resistant H58 strain of *S*. Typhi, and, as has been shown in Malawi, a relatively large burden of disease in young children. Blantyre, Malawi is a favorable location for this trial given the demonstrated high burden of typhoid fever, the established typhoid surveillance on-going in the area (including over 20 years of passive surveillance at QECH and over 18 months of enhanced surveillance, mapping, and serological studies by the STRATAA study), and the experience and capacity of the investigators and staff to conduct the

study. Another site in Thyolo district, Malawi run by the same investigators with similar capacity will be used as an additional location for immunogenicity and reactogenicity substudies in HIV exposed infants. In addition, Malawi has historically been an early introducer of new vaccines, including rotavirus vaccine.

This study is part of a multi-institutional program called the Typhoid Vaccine Acceleration Consortium (TyVAC), funded by the Bill and Melinda Gates Foundation (BMGF), and led by the University of Maryland, designed to generate evidence for the impact of Vi-TCV to support the use of Vi-TCV in countries with endemic typhoid. This study in Malawi will be one of 3 sites with endemic typhoid chosen for field efficacy studies in the TyVAC program; the other sites are Kathmandu, Nepal and Dhaka, Bangladesh. The sites differ by demography, geography, and epidemiological settings, which will enhance the generalizability of the results of these studies across populations.

2.2.2. Rationale for Study Vaccine

Vi-TCV (TypBar-TCV™) is an attractive vaccine because of its favorable safety profile, one dose schedule, and licensure down to 6 months of age, making it feasible for incorporation into the routine EPI schedule. Further, Vi-TCV is licensed in India and several other countries in Asia and is the only TCV currently undergoing review by WHO for pre-qualification, which is anticipated to occur prior to or during the course of this study. In December 2017 the WHO pre-qualified Typbar-TCV™ and in March 2018 recommended the introduction of the prequalified Typbar-TCV™ for infants and children beginning at six months of age in campaigns and routine vaccinations in endemic countries. Two countries in Africa, Liberia and Zimbabwe, introduced TCV into their immunization programs in 2021. Malawi has made a decision to introduce TCV into their immunization program and has received financial support from Gavi . There will be a national campaign among children beginning at 9 months of age, followed by an introduction in the National EPI programme. The anticipated start of the campaign is likely to be September 2022.

2.2.3. Rationale for Control Vaccine

The meningococcal group A conjugate vaccine (MCV-A), trade name MenAfriVac®, is a single dose vaccine, licensed for use from 9 months of age that is pre-qualified by WHO and provides protection against disease caused by group A *N. meningitidis*. Meningococcal A infection is a serious disease with high case fatality rates and long-term sequelae among survivors. Since 2010, MCV-A has been administered to over 250 million children in sub-Saharan Africa, where it has been well-tolerated and highly effective at preventing meningococcal A infection in those populations.[18]

2.3. Potential Risks and Benefits

2.3.1. Potential Risks

2.3.1.1. Potential Risks of TCV

Like any vaccine, Vi-TCV may cause side effects in some children. The safety of Vi-TCV, manufactured by and licensed by Bharat-Biotech, has been assessed in clinical trials in thousands of subjects. [15] The adverse reactions were predominantly minor and transient. Local reactions such as injection site pain, erythema, and induration usually resolved within 48 hours of vaccination. Elevated oral temperature, at least 38 ˚C, has been observed in approximately 1-4% of vaccinees. [19]

A post-licensure study of co-administration of Vi-TCV with measles-rubella containing vaccine was conducted, as was a phase IV comparator study with the WHO pre-qualified Vi-PS vaccine. In both of these studies, no safety signals were reported and the safety profile of Vi-TCV was comparable to the respective comparator vaccine within the respective age groups. Post-marketing surveillance of Vi-TCV based on approximately 3000 reports has shown fever, pain and swelling around the injection site to occur in 1-10% of vaccinees in any age group with no serious adverse events reported to the manufacturer. [20] In the Oxford challenge study [17], vaccination was well tolerated with no side effects above those shown by comparator Vi-PS vaccines. WHO's Global Advisory Committee on Vaccine Safety (GACVS) evaluated data presented on Vi-TCV and found no safety signals related to the vaccine. [20] Trained clinical staff will be on-site during immunizations, conducted at premises equipped for any immediate and/or emergency care that may be needed. If care is needed, the participant will be transported to the nearest and most appropriate health facility for care. If additional urgent care is needed, the subject will be transported to the regional tertiary hospital (Queen Elizabeth Central Hospital) for emergency care. The study will provide this transportation at no cost to the participant's family. If Vi-TCV causes an immediate reaction or unknown side effect, the participant will be assessed and treated at no cost to the participant or his/her family.

2.3.1.2. Potential Risks of MCV-A

The most common reaction following receipt of MCV-A (control vaccine) was tenderness, occurring in 2-30% of recipients between 1 and 29 years of age. Other reactions noted were injection site pain and erythema of mild to moderate severity and headache of mild to moderate severity. [21]. Anaphylaxis occurs at a rate of 1 per 500,000 doses distributed. MCV-A is prequalified by the WHO.

2.3.1.3. Potential Risks to Participant Privacy

To ensure follow-up of the correct study participant, personal identifiers, including name, birth date, sex, name of parents/guardians, and location/address of residence will be collected and recorded on electronic data collection forms. As a result, a potential risk for "loss of confidentiality" exists. To avoid this risk, participants will be assigned a unique study participant number that will be used to identify the participant and link, using a master linking document, an individual to his/her study data and/or biological specimens. Whenever feasible, use of identifiers will be avoided and the unique study participant numbers will be used instead. Names and addresses will only be viewable to study staff, the Sponsor, and representatives of the Sponsor when

reviewing study data on-site. These identifiers will not be included in study information sent off-site, including copies of the study data and reports/publications. Paper-based records will be kept in a secure location and only be accessible to personnel involved in the study. Computer-based files will only be made available to personnel involved in the study through the use of access privileges and passwords. Individual participants will not be identified in any study-related reports, and all study results will be reported in aggregate only.

Biological specimens will be identified by study participant number and specimen collection date only. The specimens will be stored at the MLW laboratory, where they will be processed and stored at -70˚C. Aliquots from children in the 9-11-month old immunogenicity subset and in the HIV-exposed substudy will be shipped and stored at the University of Maryland CVD for measles and rubella testing. [22]. When stored at MLW or CVD, the specimens will be under the responsibility of the investigator. The links between study data and the study specimens will not be destroyed, but no personal-identifying information associated with the study will be stored with the specimens.

2.3.2. Known Potential Benefits

All participants enrolled will have the benefit of receiving Vi-TCV or MCV-A. The Vi-TCV is anticipated to provide protection against typhoid fever, and the control vaccine can provide protection against group A meningococcal disease. There is no routine vaccination program for typhoid or serogroup A meningitis in Malawi

For the duration of the study, all participants will have access to a study physician and nursing team who will be responsible for the enhanced passive surveillance for febrile illness at their local health center. All study participants will receive the standard of care in Malawi, which entitles them to government-funded universal healthcare.

In the event of illness requiring tertiary care, patients will be referred and transported, if necessary, to the tertiary health care facility (Queen Elizabeth Central Hospital, QECH) where they will receive full standard-of-care treatment, enhanced by additional transport, staffing and diagnostics provided by clinical trial study staff. The enhanced passive surveillance teams are embedded in the routine flow of patients in the health care facility and at QECH, in order to maximize their access to full local standard-of-care while supplementing staffing, diagnostics and transport as an additional benefit to study participants

Additionally, the trial will help improve the understanding of the impact of the Vi-TCV vaccine on typhoid infection rates, and help guide future implementation of Vi-TCV vaccination programs. Whilst participants would not necessarily benefit directly from this, a positive result from this trial could lead to implementation of a Vi-TCV vaccine program in typhoid endemic areas, providing direct and indirect protection to both those close to the participant and those in the wider population.

Interim results from a Phase 3 study conducted in Nepal found that a single dose of typhoid conjugate vaccine was safe and efficacious, preventing 81.6% of typhoid cases among vaccinated children [23]. While these results are important, the critical public health questions now become duration of protection and degree of protection in children younger than 2 years of age. In order to not delay the availability of results for African populations, the study analysis will move forward at the initial timepoint, as planned. In addition, surveillance will continue for an additional year to determine longer-term protection and to increase the power for age-specific analyses.

3. Objectives

3.1. Study Objectives

3.1.1. Primary Objective

To determine the efficacy of Vi-TCV in reducing rates of symptomatic, blood culture-confirmed *S.* Typhi infection among children who receive Vi-TCV compared to children who receive MCV-A.

Efficacy will be evaluated by comparing the incidence rate of symptomatic, blood culture-confirmed *S*. Typhi infection among children who receive Vi-TCV compared to children who receive MCV-A during the study period. The case definition will be defined as:

- Subjective fever for ≥72 hours OR
- objective temperature ≥38˚C axillary OR
- Hospitalization with history of fever of any duration

3.1.2. Secondary Objectives

1. To determine the safety profile of vaccination with Vi-TCV or MCV-A.

The safety profile of Vi-TCV will be measured by comparing the proportions of participants who experience solicited and unsolicited local and systemic reactions among children who receive Vi-TCV and those who receive MCV-A in the following categories:

- Immediate reactions that occur within 30 minutes of administration of study vaccine, observed and evaluated by study staff or reported by the subject.
- Local and systemic solicited and unsolicited events that occur within 7 days of receipt of study vaccine in a subset of participants, observed by study staff or reported by the subject's parent/guardian to study staff.
- Other non-serious adverse events that occur within 28 days of receipt of study vaccine in a subset of participants, observed by study staff or reported by the subject's parent/guardian to study staff.
- Serious adverse events that occur within six months of receipt of study vaccine that are observed by study staff or reported by the subject's parent/guardian to study staff.

2.

3. To determine the immunogenicity of Vi-TCV in a subset of participants, by age group and HIV status, as measured by serum anti-Vi IgG antibodies (percent seroconversion and GMT) at approximately 28 days following vaccination.To determine the number of blood culture confirmed cases of typhoid fever prevented during the study period by comparing the incidence of blood culture-confirmed typhoid fever in participants receiving Vi-TCV to the incidence in participants receive MCV.

3.1.3. Exploratory Objectives

1. To determine the effect of Vi-TCV on hospitalizations due to blood culture-confirmed typhoid fever and the number of hospitalizations due to typhoid fever prevented by Vi-TCV.
2. To determine the effect of Vi-TCV on the duration of hospitalization for febrile illness during the study period.
3. To determine the effect of Vi-TCV for preventing all-cause hospitalizations and the number of hospitalizations prevented during the study period
4. To describe the clinical characteristics of blood culture-confirmed typhoid fever in study participants, including the percentage of participants with specific signs and symptoms.
5. To determine the effect of Vi-TCV on outpatient visits for fever and the number of visits prevented during the study period.
6. To determine the effect of Vi-TCV on number of outpatient and hospitalized cases of clinically diagnosed typhoid fever
7. To determine the effect of Vi-TCV on hospitalizations for febrile illness and the number of hospitalizations prevented during the study period.
8. To determine the effect of Vi-TCV on antibiotic usage and the number of antibiotic courses and days of antibiotic use prevented during the study period.
9. To determine the effect of Vi-TCV on all-cause mortality and the number of deaths prevented during the study period.
10. To compare the number of episodes of illness for which blood cultures are collected during the study period between Vi-TCV and MCV-A groups.
11. To compare the incidence of hospitalizations for meningitis between Vi-TCV and MCV-A groups.
12. To evaluate the rate and recurrence of ileal perforations secondary to typhoid fever.
13. To determine the effect of Vi-TCV on the incidence of complications of typhoid fever (e.g., perforations, acute abdominal procedures, death) and the number of complications prevented during the study period.
14. To determine the persistence of serum anti-Vi IgG antibodies in a subset of participants, by age group, at approximately 2-3 years following vaccination.
15. To evaluate the efficacy and above outcomes by age groups.
16. To compare the anti-measles IgG percent seroprotection and GMCs and PRN titers among children receiving Vi-TCV + measles-rubella vaccine, and children receiving MCV-A + measles-rubella vaccine, including children enrolled in the HIV-exposed substudy.
17. To compare the anti-measles IgG percent seroprotection and GMCs among children receiving Vi-TCV + measles-rubella vaccine, and children receiving MCV-A + measles-rubella vaccine, including children enrolled in the HIV-exposed substudy.
18. To determine the immunogenicity of Vi-TCV in a subset of participants, by age group and HIV status, as measured by assays of cellular and antibody functional immunity at

approximately 28 days following vaccination, including children in both immunogenicity substudies.

19. To evaluate the relationship between serum anti-Vi IgG at 28 days post-vaccination and the development of symptomatic, blood culture-confirmed *S.* Typhi infection among children in the immunogenicity subset.
20. To quantify typhoid-specific immune responses among a subset of children with blood culture confirmed S. Typhi infection and compare between children receiving Vi-TCV and children receiving MCV-A.
21. To determine the rate of serum anti-Vi IgG antibody decay over the study period among a subset of children meeting blood-culture-collection criteria.
22. To study serological exposure over the study period to iNTS and novel Coronavirus SARS-CoV-2 (COVID-19), among a subset of children meeting blood-culture-collection criteria.
23. To assess host factors affecting acquisition of enteric fever through the identification of human genes that either increase or decrease a person's risk of getting typhoid and understanding how these gene variants affect biological function.
24. To determine the number of blood culture-confirmed cases of typhoid fever by study site and by high and low incidence settings.
25. To determine the extended efficacy of Vi-TCV in reducing rates of symptomatic, blood culture-confirmed S. Typhi infection among children who receive Vi-TCV compared to children who receive MCV-A.

3.2. Study Outcome Measures

3.2.1. Primary Outcome Measure

The primary outcome of interest is the incidence of blood culture-positive typhoid fever among Vi-TCV and MCV-A vaccine recipients in the study population through April 3, 2020. Vaccine efficacy will be calculated as one minus the relative rate of symptomatic typhoid fever in the Vi-TCV group compared to that in the MCV-A group.

3.2.2. Secondary Outcome Measures

3.2.2.1. Secondary Outcome Measure #1

The safety profile of Vi-TCV will be measured by comparing the proportions of participants experiencing solicited and unsolicited local and systemic reactions between children receiving Vi-TCV as compared to children receiving MCV-A according to the following three categories:

1. The proportion of participants who develop adverse events detected in the first 30 minutes after vaccination and for 7 days after vaccination.
2. The proportion of participants who experience serious adverse events within 6 months of vaccination in all participants.
3. The proportion of participants who experience other non-serious adverse events up to 28 days following vaccination, in a subset of participants.

3.2.2.2. Secondary Outcome Measure #2

The immunogenicity of Vi-TCV in a subset of participants measured by ELISA for anti-Vi percent seroconversion and GMTs before and at day 28 after vaccination.

3.2.2.3. Secondary Outcome Measure #3

The number of blood culture-confirmed cases of typhoid fever prevented by Vi-TCV during the study period measured by comparing the incidence of blood culture-confirmed typhoid fever in participants receiving Vi-TCV compared to those receiving MCV-A.

4. Overview of Study Design

4.1. Efficacy Study

The study is a double-blind, individually randomized, controlled, clinical efficacy trial involving up to 28,500 participants with two vaccine groups: Vi-TCV and MCV-A. Participants will be randomized in a 1:1 ratio to receive Vi-TCV or MCV-A. Children 9 months through 12 years of age in the Blantyre area who meet the inclusion criteria (Section 5.1) will be vaccinated. MCV-A does not confer any protection against *S.* Typhi, and so it may be used as a control in the efficacy study.

Participants will be unaware of which study vaccine, Vi-TCV or MCV-A, is received. Unblinded study staff who will not be involved in participant follow-up will prepare and administer vaccine to prevent unblinding of children, parents, or study staff who will participant in participant follow-up. Serious adverse events (SAEs) will be monitored through the end of the trial.

All children will be monitored for 30 minutes following vaccination to monitor for serious adverse events. The parent/guardian of each participant will be given information about how to contact clinic personnel to report side effects to vaccine or symptoms of typhoid fever.

For the evaluation of efficacy, passive surveillance will be conducted throughout the study to identify outcomes among vaccinated subjects. In the study area, all children who meet the protocol-defined specimen collection criteria will have a blood culture collected at the outpatient facilities or QECH. Additional information will be collected from any child who has a blood culture obtained. This will include information about the signs and symptoms of the illness and treatment given. Likewise, any child with blood culture-confirmed typhoid fever will have follow-up until the illness resolves. Additional information on the illness, treatment and complications will be recorded. Two sets of vaccine efficacy (VE) analyses will be conducted. Due to the impact of the prolonged interruption of surveillance activities caused by COVID-19, the first set of VE analyses was evaluated through April 3, 2020, when the pre-specified number of cases is reached after a minimum of 18 months of follow-up on each participant. The second efficacy analysis will occur through September 30, 2021, the end date for the analysis including all subgroups, when all participants have been followed for a minimum of 36 months, and a third analysis will occur at the end of the extended surveillance period, through December 31, 2021.

4.2. Immunogenicity and Reactogenicity Substudy

A subset of 600 children (200 in each of three age groups: 9-11 months, 1-5 years, and 6-12 years) will be included in an additional Vaccine Immunogenicity and Reactogenicity Substudy. The purpose of this detailed evaluation is to assess the immune responses to Vi-TCV, as 2determined by GMT and seroconversion (defined by ≥4-fold response). These children will also undergo additional screening to ensure a subset where immunogenicity can be most accurately determined. In addition to the passive surveillance for typhoid fever, this group will have additional follow-up visits. Serum specimens will be collected on day 0 (before vaccination) and on post-vaccination days 28 and 730 from all children included in the substudy. Due to the study interruption because of the outbreak of COVID-19, the long-term immunogenicity time point will be extended to include specimens drawn at any time from day 730 until day 1095. These specimens will be tested for the presence of serum IgG anti-Vi antibody and for functional assays of humoral and cellular immunity. In the 9-11-month-old age group, specimens on days 0 and 28 will also be tested for measles and rubella antibodies.

In addition, a more detailed assessment of vaccine reactogenicity will be made among these children by actively collecting solicited and unsolicited reactions at each study visit in the week after vaccination (days 3 and 7) and on day 28 and day 180. Serious adverse events will also be solicited on days 28, 180 and 730. Due to the study interruption because of the outbreak of COVID-19, serious adverse events will also be solicited until the final study visit – up to day 1095 for participants.

4.3. Antibody Kinetics Substudy

The duration of vaccine-induced immunity is an important public policy question. While the immunogenicity substudy will help us to understand the immediate antibody response and the remaining antibody at 2-3 years, obtaining additional samples at multiple points in time will allow us to better understand the kinetics of vaccine-induced antibody. An Ethylenediaminetetraacetic acid (EDTA) sample will be requested as an opt-in sample collection for children who meet blood culture collection criteria under the current protocol at every visit to enable us to study the kinetics of vaccine-induced antibody. These samples will also be used to track ongoing serological exposure to iNTS and COVID-19. For parents and children who consent to this substudy, an additional 3-5 ml of blood will be obtained. Based on the current rate of febrile illness and blood cultures taken, this will provide up to several thousand additional blood samples at various points in time, from approximately 3 months to 45 months post-vaccination. Samples will be tested for serum IgG anti-Vi antibody and other novel antigens allowing us to estimate antibody decay over time in the population. Depending on the number of typhoid-positive cases, these samples may allow us to identify a correlate of protection. Permission will also be sought from parents/children to use some amount of this EDTA sample for human genetics, described in section 4.5. All children in the main study will be eligible for this substudy, and no additional procedures or visits, other than the additional blood draw, are required.

4.4. HIV-exposed Vaccine Immunogenicity and Reactogenicity Substudy

An additional subset of up to 325 HIV-exposed and HIV-unexposed children ages 9-11 months will be included in an additional **HIV-exposed Vaccine Immunogenicity and Reactogenicity Substudy**. The purpose of this detailed evaluation is to assess the reactogenicity of the vaccine and the immune responses to one or two doses of Vi-TCV in HIV-exposed and HIV-unexposed children. HIV-exposed children in this substudy will be randomized 1:1:1 to receive either TCV at 9-11 months and TCV at 15 months (Group 1), TCV at 9-11 months (Group 2), and TCV at 15 months only (Group 3). An additional group of HIV-unexposed children will be given TCV at 9-11 months and TCV at 15 months (Group 4). Serum specimens will be collected on day 0 (before vaccination) and on 28 days after each vaccination from all children included in the sub-study, and will be tested for immune responses to measles, rubella, and typhoid (groups 1-4) and presence of HIV RNA (groups 1-3). Group 4 will also have an additional sample collected at 15 months prior to second vaccination. For this substudy, Vi-TCV will be administered with measles-rubella-containing vaccine #1 at 9-11 months and/or #2 at 15 months, as per Malawi EPI schedule. These 9-11-month-old children will have antibody to measles and rubella assessed on day 0 and 28 days after each vaccination. All children in the sub-study will be assessed at 7 days after each vaccination for solicitation of local and systemic adverse events. Serious and non-serious adverse events will be assessed up to the last study visit for HIV-exposed substudy participants. Analyses will use all data collected through March 2022 and will be a secondary outcome of the study.

Table 2: Visit schedule for HIV-exposed sub-study

Study Visit Number	Study Time point	Study procedures	Group 1 (TCV at 9-11months and 15 months)	Group 2 (TCV at 9-11months)	Group 3 (TCV at 15 months)	#Group 4 (TCV at 9-11months and 15 months)
V1	D0	*Serum for immunogenicity assessment	X	X	X	X
		Serum for testing presence of HIV RNA	X	X	X	
		Vaccination (9-11 months)	√	√		√
		Assess and record SAEs	X	X	X	X
		Assess and record solicited AEs	X	X	X	X
V2	D7	Assess and record solicited AEs	X	X	X	X

V3	D28	**Serum for immunogenicity assessment	X	X	X	X
		Assess and record SAEs	X	X	X	X
		Assess and record solicited AEs	X	X	X	X
V4	D180	*Serum for immunogenicity assessment				X
		Vaccination (15 months)	√		√	√
		Assess and record SAEs	X	X	X	X
		Assess and record solicited AEs	X	X	X	X
V5	D187	Assess and record solicited AEs	X	X	X	X
V6	D208	**Serum for immunogenicity assessment	X	X	X	X
		Serum for testing presence of HIV RNA	X	X	X	
		Assess and record SAEs	X	X	X	X
		Assess and record solicited AEs	X	X	X	X

X Sample collection
√ Vaccination
*Pre-vaccination
**Post-vaccination
Mothers will have a rapid HIV test as well

4.5. Human Genetics

Human genetics will be done to establish the host factors that determine susceptibility to enteric fever. These samples will join a larger dataset to understand this question. In this study, all children whose samples are included in the antibody kinetics sub- study will be eligible for this genomics testing and no additional procedures, visits, and blood draw or samples, are required. Cell pellets will be collected and stored, with specific consent, from the EDTA sample collected at passive surveillance.

5. Enrollment and Withdrawal

5.1. Subjects

Following community sensitization using IRB-approved materials, local community field workers will systematically approach each household to identify children aged 9 months through 12 years. When a household with a potential child participant is identified, the parent/guardian of the child will be given information about the trial orally and in writing. Participants may also be provided information in a group setting, using spoken or approved recorded material. The

parents of eligible participants will be given an opportunity to ask questions and provide written informed consent in a semi-private area prior to enrollment and vaccination.

5.1.1. Inclusion Criteria

Each subject receiving study product (Vi-TCV or MCV-A) must satisfy all of the following inclusion criteria:

- Healthy male or female child between the ages of 9 months and 12 years/364 days at the time of study vaccination.
- A child whose parent or guardian resides primarily within the Ndirande or Zingwangwa study areas at the time of study vaccinations and who intends to be present in the area for the duration of the trial.
- A child whose parent or guardian has voluntarily given informed consent.

5.1.2. Additional inclusion criteria for HIV-exposed vaccine safety and immunogenicity substudy

In addition to the inclusion criteria of the efficacy study, HIV exposed children whose parents or guardians reside primarily within Bvumbwe study area in Thyolo district at the time of study vaccinations and who intend to be present in the area for the duration of the trial may be enrolled into the HIV-exposed vaccine immunogenicity and reactogenicity substudy.

The 100 participants enrolled in group 4 of the HIV-exposed vaccine immunogenicity and reactogenicity substudy must satisfy the following inclusion criteria:

- A child whose biological mother has voluntarily given informed consent for her rapid HIV antibody finger prick test.
- A negative rapid HIV antibody finger prick test for biological mother of child.

5.1.3. Exclusion Criteria

No subject receiving study vaccine (Vi-TCV or MCV-A) may have any of the following exclusion criteria at study entry:

- History of documented hypersensitivity to any component of the vaccine
- Prior receipt of any typhoid vaccine in the past 3 years
- History of severe allergic reaction with generalized urticarial, angioedema, or anaphylaxis
- Any condition determined by the investigator to be likely to interfere with evaluation of the vaccine or to be a significant potential health risk to the child or make it unlikely that the child would complete the study.

5.1.4. Temporary Exclusion Criteria

The following will be considered temporary contraindications to enrollment and vaccination. If these apply, the participant will be temporarily excluded for vaccination until 48 hours has passed. A re-assessment will be needed to ensure these temporary exclusion criteria no longer exist.

- Reported fever within 24 hours prior to vaccination
- Use of anti-pyretics within 4 hours prior to vaccination

An additional temporary exclusion criteria will be:

- Receipt of measles-rubella vaccine in the one month prior to enrollment, as determined by parental history or vaccination card.

5.1.5. Additional Exclusion Criteria for Safety and Immunogenicity Substudy

In addition to the exclusion criteria of the efficacy study, participants enrolled in the immunogenicity and reactogenicity substudy may not have, or have had, any:

- Known history of diabetes, tuberculosis, cancer, chronic kidney, heart, or liver disease, progressive neurological disorders, poorly controlled seizures, or terminal illness
- Severe malnutrition as determined by MUAC< 12.5 cm for children younger than 5 years;
- Receipt of any other investigational intervention in the last 6 months or anticipated during the course of the study.
- Receipt of blood products in the last 6 months.
- Known HIV-infection or exposure or other immunosuppressive conditions.
- Receipt of systemic immunosuppressant or systemic corticosteroids.
- Receipt of any measles-rubella-containing vaccine for children younger than 1 year of age

If a child is not eligible for the immunogenicity and reactogenicity substudy, they will continue in the efficacy follow-up.

5.1.6. Additional Exclusion Criteria for HIV-exposed Vaccine Safety and Immunogenicity Substudy

In addition to the exclusion criteria of the efficacy study, up to 225 participants enrolled in Groups 1-3 of the HIV-exposed vaccine immunogenicity and reactogenicity substudy will have evidence of maternal HIV exposure as demonstrated by documentation in the child’s or mother’s health passport. The 100 participants in Group 4 will have no evidence of maternal HIV exposure as documented in the mother’s health passport (negative HIV test done and recorded in the last 3 months from the time of clinic visit) or determined by mother’s negative point of care test (rapid HIV antibody finger prick test) during the clinic visit for those who do not have a negative HIV test recorded within the last 3 months. In addition, the child may not have, or have had, any:

- Known history of diabetes, tuberculosis, cancer, chronic kidney, heart, or liver disease, progressive neurological disorders, poorly controlled seizures, or terminal illness
- Receipt of any other investigational intervention in the last 6 months or anticipated during the course of the study.
- Receipt of blood products in the last 6 months.
- Receipt of systemic immunosuppressant or systemic corticosteroids.
- Previous receipt of any measles-rubella-containing vaccine
- Known positive HIV RNA test (n=up to 225, Groups 1-3)
- Mother's positive rapid testing for HIV at screening (n=100, Group 4)

5.2. Treatment Assignment Procedures

5.2.1. Randomization

Eligible participants will be randomized by random number generators at a 1:1 ratio to receive Vi-TCV or MCV-A. Randomization will be generated by computer to allocate participants to either the Vi-TCV group of the MCV-A group in a 1:1 ratio using stratified block randomization with varying block sizes from 6-20.

A subset of 600 participants who receive Vi-TCV and MCV-A will be randomly chosen within each age stratum and screened for additional inclusion/exclusion criteria for the safety and immunogenicity substudy. If eligible, they will be further consented and randomization will proceed as above.

An additional subset of up to 325 participants aged 9-11 months will be screened for additional inclusion/exclusion criteria for the HIV-exposed vaccine safety and immunogenicity substudy. If eligible, they will be further consented and randomization for the up to 225 HIV-exposed children will proceed 1:1:1 to receive either TCV at 9-11 months and TCV at 15 months (Group 1, n= up to 42), TCV at 9-11 months (Group 2, n= up to 42), or TCV at 15 months (Group 3, n= up to 42). A fourth group of HIV-unexposed children will receive TCV at 9-11 months and TCV at 15 months (Group 4, n=100).

Study participants, their family members, and study staff will be unaware of the assigned vaccine group. Staff who prepare and administer vaccines will not be involved in subsequent study procedures.

An unblinded version of the allocation sequence (i.e., a table containing the randomization number and vaccine or placebo allocation) will be maintained by an unblinded CVD scientist in a secure location for use in unblinding according to protocol (i.e., for a safety event of concern or following database lock at the end of the trial) or for assignment of replacement vials should the original assigned vaccine be damaged or prepared incorrectly. This CVD staff member will not be part of the clinical team that participates in the trial activities and will not share the list with any CVD or site clinical team members responsible for study conduct. Strict procedures for controlling access to the allocation sequence are part of the confidential SOP (Randomization Plan) for conducting the randomization.

We anticipate that any circumstances warranting unblinding will be rare. However, circumstances may arise where unblinding is needed prior to the end of the study e.g. occurrence of a Suspected Unexpected Serious Adverse Reactions or requirement of a medical intervention that would be influenced by knowledge of which vaccine the individual has received. In such circumstances, the Independent Safety Monitor (ISM), who will be a physician in Malawi who is not an investigator for this study, will assess the child. The details will then be reported to the Sponsor and the Data Safety Monitoring Board and discussed. Then if deemed necessary, the DSMB will recommend unblinding to the study sponsor. Any event of unblinding will be fully documented in the CRF.

5.2.2. Masking

Vi-TCV and MCV-A will have different preparations and presentations in this trial. Therefore, unblinded study personnel will be responsible for vaccine preparation and administration. These unblinded personnel will not be involved in study-related assessments or have contact with subjects for data collection following study vaccination.

The Data and Safety Monitoring Board (DSMB) may receive data in aggregate and presented by treatment arm. The DSMB may also be provided with expected and observed rates of the expected AEs in an unblinded fashion. The DSMB will review grouped and unblinded data in the closed session only.

5.2.3. Adherence to Randomization

Study vaccines will be coded with allocation codes and study vaccine given to each individual subject will be documented on the CRF with the appropriate allocation code. A log will be kept which lists each child’s study number, allocation code received, date of receipt of the allocation, and other relevant information (such as mother, father, village, compound, etc.) to keep track of allocations received by all study participants.

5.3. Subject Withdrawal

5.3.1. Reasons for Withdrawal

Participants’ parents/guardian may withdraw consent at any point.
The Investigator may also discontinue a participant from the trial at any time if the Investigator considers it necessary for any reason including:

- Ineligibility (either arising during the trial or retrospectively having been overlooked at screening),
- Significant protocol deviation,
- Significant non-compliance with trial requirements,
- An adverse event or disease progression resulting in the inability to continue to comply with trial procedures and follow-up,
- Loss to follow up.

5.3.2. Handling of Withdrawals

Withdrawal will result in cessation of any follow-up calls or blood tests (as applicable to the subset), although we will ask permission to continue safety follow-up. Participants' parents/guardians will have the choice when withdrawing, to withdraw from active study procedures only (follow-up calls and immunogenicity blood draws) but remain in the passive surveillance for the primary outcome, (allowing us to access their hospital records and blood test results), or withdraw from all study contact. In the case of a participant withdrawing from all study contact, we will not collect any further data of hospital presentation or blood culture results. Data and blood samples collected prior to the time of participant withdrawal will be kept and analyzed as part of the study data. A participant who withdraws from the study has the option to re-engage at a future date if they choose to do so. All participants who withdraw from the study will be given information on how to re-engage with the study if they so choose. Reasons for withdrawal from the study, if known, will be recorded in the participants CRF.

5.4. Termination of the Trial

5.4.1. Termination According to the Protocol

The study will end once all children have been followed for at least 51 months after vaccination, and the pre-specified number of cases of *S*. Typhi infection is met. Participants in the HIV-exposed substudy will reach study end at 28-180days after the second dose of measles/rubella vaccine #2 scheduled at 15 months of age.

5.4.2. Suspension and/or Premature Termination

The safety oversight and proposed halting rules have been written to protect the safety of trial participants while acknowledging that these trials are being conducted in areas with high childhood morbidity and mortality. Serious adverse events and even deaths will occur with some frequency at the trial site. We have prior information on the safety of the vaccine through previous clinical trials, and the post marketing surveillance in India, with over 3 million doses distributed.

If any of the following halting criteria are met after study product is administered, the study will be suspended and further doses of vaccine will not be administered pending review of data by the DSMB.

- Within 24 hours of receiving vaccine, any two subjects experience a life-threatening anaphylactic[11] reaction related to vaccination.

[11] Anaphylaxis will be defined by Brighton Collaboration criteria. [24. Ruggeberg, J.U., et al., *Anaphylaxis: case definition and guidelines for data collection, analysis, and presentation of*

- Within 7 days of vaccination, three or more subjects experience study-product-related SAEs within a single MedDRA category (e.g. gastrointestinal; respiratory; other infections and infestations).

Subsequent review of serious, unexpected, and related AEs by the ISM, DSMB, ethics review committee, the sponsor(s), or the relevant local regulatory authorities may also result in suspension of further trial interventions/administration of study product at a site.
The regulatory authorities and study sponsor(s) retain the authority to suspend additional enrollment and study interventions/administration of study product for the entire study, as applicable.

6. Study Vaccines

6.1. Vaccine Descriptions

6.1.1. Typbar-TCV™

Typbar-TCV™ consists of 25 µg of Vi polysaccharide conjugated to a nontoxic tetanus toxoid protein carrier. Vi-TCV is given as a 0.5-ml dose by the intramuscular route to infants, toddlers, children, and adults. This vaccine will be shipped from the manufacturer in five-dose, 2.5 mL vials.

6.1.2. MenAfriVac Meningococcal Group A Conjugate Vaccine

The control vaccine for this study is Meningococcal Group A conjugate vaccine (MenAfriVac, Serum Institute of India PVT Ltd). This lyophilized vaccine consists of meningococcal group A polysaccharide conjugated to tetanus toxoid protein in aluminum phosphate adjuvant. This vaccine is produced in two formulations; a standard 10µg/0.5 ml dose for individuals aged ≥1 year of age (for use in campaigns); and a 5µg/0.5ml single dose for individuals aged 9 – 24 months (for use in routine immunization programs).

6.1.3. Acquisition of Study Vaccines

All required doses of the study investigational vaccine will be donated by the manufacturer, Bharat Biotech International (Hyderabad, India). The control vaccine will be purchased by the Sponsor from the manufacturer, Serum Institute of India Limited (Pune, India).

---

immunization safety data. Vaccine, 2007. **25**(31): p. 5675-84. Anaphylaxis is a clinical syndrome characterized by sudden onset, rapid progression of signs and symptoms, major dermatologic involvement (generalized urticaria or erythema, angioedema, generalized pruritis with skin rash) AND major involvement of the cardiovascular system (measured hypotension and uncompensated shock) AND/OR the respiratory system (bronchospasm/stridor/upper airway swelling or respiratory distress with 2 or more of: tachypnea, recession, cyanosis, grunting, increased use of accessory muscles).

6.1.4. Packaging and Labelling

6.1.4.1. Packaging

Typbar-TCV™ is packaged in a five-dose (2.5-mL) vial. MenAfriVac® comes in a 10-dose vial, which contains lyophilized Meningococcal Group A polysaccharide conjugated to tetanus toxoid along with aluminum phosphate. The lyophilate is reconstituted just before use. One ampule with diluent contains 10 doses of vaccine.

6.1.4.2. Labelling

The vaccines will be labelled by the manufacturer and will not be relabeled.

6.1.5. Storage and Stability

Vi-TCV and MCV-A will be stored at 2-8 ˚C in a temperature monitored refrigerator. MCV-A is stable up to 40 ˚ C for a period of 4 days immediately prior to reconstitution. A temperature sensitive label is attached to each vial to indicate heat exposure and likely inactivation. Open vials will be used within 6 hours when stored under refrigeration. Refrigeration temperature and cold chain will be monitored during the study.

6.2. Dosage, Preparation, and Administration of Study Vaccines

6.2.1. Dosage

The appropriate dosage of Vi-TCV is 0.5 mL, which includes 25 µg purified Vi capsular polysaccharide of *S*. Typhi conjugated to tetanus toxoid. Children 1 year of age and over will receive MCV-A a 10µg/0.5 ml dose and children < 1 year of age will receive a 5µg/0.5ml dose.

6.2.2. Preparation

Vi-TCV is a Vi polysaccharide-tetanus toxoid conjugate vaccine which contains purified Vi-capsular polysaccharide of S. Typhi Ty2 conjugated to tetanus toxoid 25 µg, sodium chloride 4.5 mg, 2-phenoxyethanol 5.0 mg, and water for injection 0.5 mL. The vaccine is packaged as a 2.5 mL five-dose vial.

MCV-A comes in a 10-dose vial which contains lyophilized Meningococcal Group A polysaccharide conjugated to tetanus toxoid along with aluminum phosphate. The lyophilate is reconstituted just before use.

6.2.3. Administration

Both study vaccines (Vi-TCV and MCV-A) should be administered as intramuscular injections to either the anterolateral thigh of younger children or upper arm of older children.

6.3. Accountability procedures for the Study Vaccines

The vaccines will be cold-shipped to Malawi and cleared by MLW facilities staff and agents. They will then be transported to MLW and distributed to local clinics and vaccination sites while maintaining the cold-chain (aiming for temperature between 2-8˚C), with the exception of the MCV-A diluent which will be kept at temperatures below 40˚C. The number of doses of study vaccines that are received, used and wasted will be documented daily during the trial and checked weekly. Unused vaccines at the end of the trial may be retained for laboratory use only (such as laboratory assay development). Any recall of study vaccines required for use in the study or reporting of defective vaccines will be performed according to trial SOPs.

6.4. Concomitant Medications and Vaccinations

Concomitant medications and vaccinations will be checked and recorded on CRFs. Measles-rubella vaccine can be immunosuppressive, therefore receipt of measles-rubella vaccine within 30 days prior to study enrollment will be a temporary exclusion criteria. The participant may be enrolled and vaccinated once 30 days have passed since receipt of measles-rubella vaccine. For some participants, study vaccine will be administered when they present to the clinic for the routine 9-month measles-rubella vaccine visit, and study vaccine may be administered on the same day.

7. Study Schedule

7.1. Recruitment and Screening

Recruitment strategies will be broad and varied. The principal form of recruitment will occur in schools, community centers, and healthcare centers. Study field workers may also systematically approach households within the catchment area either house by house or Health Surveillance Assistants (HSA) area to HSA area to identify children aged 9 months through 12 years to determine their eligibility to participate in the study. Parents of age-eligible children will be given general information about the trial and the eligibility criteria. If interested, they will be invited to a study vaccination center. If they are unable to attend the vaccination center, they may alternatively be consented in their home, their child’s school, or at community and healthcare centers by visiting study staff. The study will be explained and the requirements discussed. (See Section 13.4 for more details on informed consent). Study staff will answer any questions about the trial, procedures and risks. The parents/guardians of the children will then have the opportunity to provide informed consent. After informed consent and assent (if applicable) are obtained, each potential participant will be screened for eligibility according to the inclusion/exclusion criteria. Those who are eligible may proceed to enrollment.

Potential participants in the HIV-exposed Safety and Immunogenicity Substudy will undergo HIV testing at screening. For recruitment into groups 1-3 (HIV-exposed), they will have HIV RNA testing to determine HIV infection status, but this will not be used to determine eligibility. For recruitment into group 4, mothers of potential participants who do not have a negative HIV test

done and recorded within the last 3 months will have rapid HIV antibody finger-prick testing after consenting to determine eligibility of their child before enrollment. Those who test HIV-positive but are not yet on antiretroviral treatment according to national guidelines will be counseled per national guidelines and referred immediately to the Ndirande HIV clinic, located on the grounds of the Ndirande Health Centre.

7.2. Vaccination

7.2.1. Enrolment/Vaccination (Day 0)

Screening and enrollment visits may occur on the same day. At the vaccination visit, and prior to vaccination, informed consent will be confirmed. Assent will be obtained for all children 8 years of age and older. Study staff will review eligibility criteria with all participants, review participant medical history, concomitant medications, and measles-rubella vaccination history. Temperature will be taken for all participants. Once this is complete, and all eligibility criteria are verified, participants will be administered either Vi-TCV or MCV-A.

7.2.2. Post-Vaccination Monitoring

All study participants will be asked to remain in the study area for at least 30 minutes immediately following vaccination in the event there is a serious reaction. Any immediate reactions will be assessed and recorded.

7.3. Surveillance

For the evaluation of efficacy, passive surveillance will be conducted throughout the study to identify outcomes among vaccinated subjects. In the study area, all children who meet the protocol-defined specimen collection criteria will have a blood culture collected at the outpatient facilities or QECH. Additional information will be collected from any child who has a blood culture obtained. This will include information about the signs and symptoms of the illness and treatment given. If a blood culture is positive, inpatient participants will be followed up by study staff on the ward or if outpatient contacted by phone and followed up remotely to ensure they are on appropriate treatment. Additional information on blood-culture-confirmed typhoid illness will be obtained, and the participant followed every 2 weeks until asymptomatic. Additional information on the illness, treatment and complications will be recorded. Vaccine efficacy will be evaluated based on cases accrued at three timepoints: on through April 3, 2020 when all participants have been followed for a minimum of 18 months; through September 30, 2021, the end date for the analysis including all subgroups, when all participants have been followed for a minimum of 36 months; and until the end of the extended surveillance period, through December 31, 2021.
In addition to the blood culture, vaccinated children presenting with fever to one of the study sites whose parent/caregiver consents to allow it will have an EDTA sample taken to study the kinetics of vaccine-induced antibody. The additional sample will also be tested for serological exposure to iNTS and COVID-19.

The EDTA sample collected within the antibody kinetic sub study will also be used for host genetics analyses, provided that the parent/caregiver has given permission for genetic testing. The purpose of the host genetics component is to obtain as many confirmed typhoid cases as possible to be combined with samples collected from other studies from multiple sites to enable a sufficiently powered human genetics study. Genome wide association analysis (GWAS) data for 500 Vietnamese cases and 2000 controls genotyped on the Illumina OmniExpress which surveys 733,202 markers, is already available[27] and cases collected as part of this study will be similarly genotyped. In the multi-population GWAS discovery set that will result, we will look for associations of SNP markers using genotypic, allelic and trend tests comparing SNP frequencies in enteric fever cases and population controls across all populations collected. Genetic variants found to be associated with typhoid will be genotyped in independent replicative sample sets to confirm the genetic association. Disease causal gene variants will be identified by fine mapping and/or re-sequencing of the associated gene region/s. Following consent and enrolment into the antibody kinetics substudy, individuals will be asked to provide consent specifically for human genetics. Genomic DNA will be extracted at MLW and shipped to Professor Chiea Chuen Khor at Genome Institute of Singapore for genotyping using Illumina genotyping arrays. Genome sequence data will then be sent to Dr. Dunstan at the University of Melbourne, Australia. Genome wide association analysis will be led by the University of Melbourne in collaboration with the TyVAC trial team.

For the evaluation of the effect of vaccination on the number of outpatient visits, hospitalization and antibiotic usage, passive surveillance will also be conducted throughout the study for all study participants who visit the outpatient facilities (Ndirande, Zingwangwa and QECH) or are inpatients at QECH but do not meet the protocol-defined specimen collection criteria. Information will be collected from any study participant who visits the outpatient clinics or is an inpatient at QECH. This information will include purpose of the visit to the health facility, duration of symptoms, primary diagnosis, prescribed treatment and date of admission and discharge/loss to follow-up/death (in case of inpatient). Inpatient participants will be followed up by study staff on the pediatric wards until discharge/loss to follow-up/death.

The parent/guardian of each participant will be given information about how to contact study or clinic personnel to report side effects to vaccine or symptoms of typhoid fever and where to seek treatment.

Serious adverse events, whether or not associated with study participation, will be noted and recorded. The contact information for study personnel will be provided again and instructions regarding study participation, future visits, and instructions provided.

To ensure effective passive surveillance of vaccinated children, trial staff will use a variety of communication methods, encouraging and reminding family members to bring the children to study facilities during episodes of febrile illness, including;

- Text message reminders sent from central MLW phone number to parents/guardian’s mobile phones.

- Ministry of Health staff such as Healthcare surveillance assistants, and MLW trial staff to visit vaccinated households, schools and communities and distribute printed materials.
- Use of an MLW van with loud-speakers, giving public information to communities.
- Other individual engagement activities and meetings within the community, schools and health centres raising awareness of the study and the availability of passive surveillance for trial participants.

The wording of the messages for such communications and reminders will be approved by the Malawi NHSRC and other regulatory bodies.

7.4. Immunogenicity and Reactogenicity Substudy

7.4.1. Enrolment/Vaccination

Participants in the immunogenicity and reactogenicity substudy will be enrolled according to the same procedures as other study participants. Participants will be randomly invited to participate in the additional substudy. These participants, and their parents/guardians, will be informed of the additional requirements and procedures of this substudy. If the parent/guardian gives consent for these additional procedures, the child will be further enrolled into the substudy, up to a total of 200 in each of three age groups (9-11 months, 1-5 years, and 6-12 years).

7.4.2. Active Follow-Up

Children in the immunogenicity and reactogenicity substudy will have additional home and follow-up visits. Home or clinic visits will take place on days 3, 7, and 180 following vaccination. During these visits, solicited and unsolicited adverse events will be recorded. On days 28 and 730-1095 following vaccination participants in this substudy will come to the clinic for follow up visits during which they will have 5 mL blood drawn to measure typhoid Vi-antibody responses typhoid (all age groups) and measles and rubella antibody responses (9-11-month old age group only), which will be used for the secondary and exploratory objectives as listed. Due to the study interruption because of the outbreak of COVID-19, serious adverse events will also be solicited until the final study visit – up to day 1095 for participants.

Children in the substudy who are less than 5 years of age will have height and weight and MUAC measured at baseline and at two to three years after vaccination.

7.5. HIV-exposed Vaccine Safety and Immunogenicity Substudy

7.5.1. Enrolment/Vaccination

Participants in the HIV-exposed substudy will be enrolled according to the same procedures as other study participants. These participants, and their parents/guardians, will be informed of the requirements and procedures of this substudy. If the parent/guardian gives consent for these procedures, the child will be further enrolled into the substudy, up to a total of 325 children aged 9-11 months.

7.5.2. Active Follow-Up

Children in the HIV-exposed substudy will have additional home and follow-up visits. Home, clinic or telephone visits will take place at 7 and 28 days following each vaccination. During these visits, solicited and unsolicited adverse events will be recorded. At 28 days after each vaccination, participants in this substudy will come to the clinic for follow up visits during which they will have 5 mL blood drawn to measure typhoid Vi-antibody quantitative and functional immunological responses, and measles and rubella antibody responses.

8. Study Evaluations

8.1. Clinical Evaluations

8.1.1. Clinical Case Definitions

During the study period, any child who presents to the clinic with a fever of at least 72 hours or a temperature at least 38˚C axillary or who is hospitalized with a fever of any duration will be assessed. The participant will receive standard of care management. Study staff will collect information about the symptoms, including temperature, duration, hospitalization, antibiotic use.

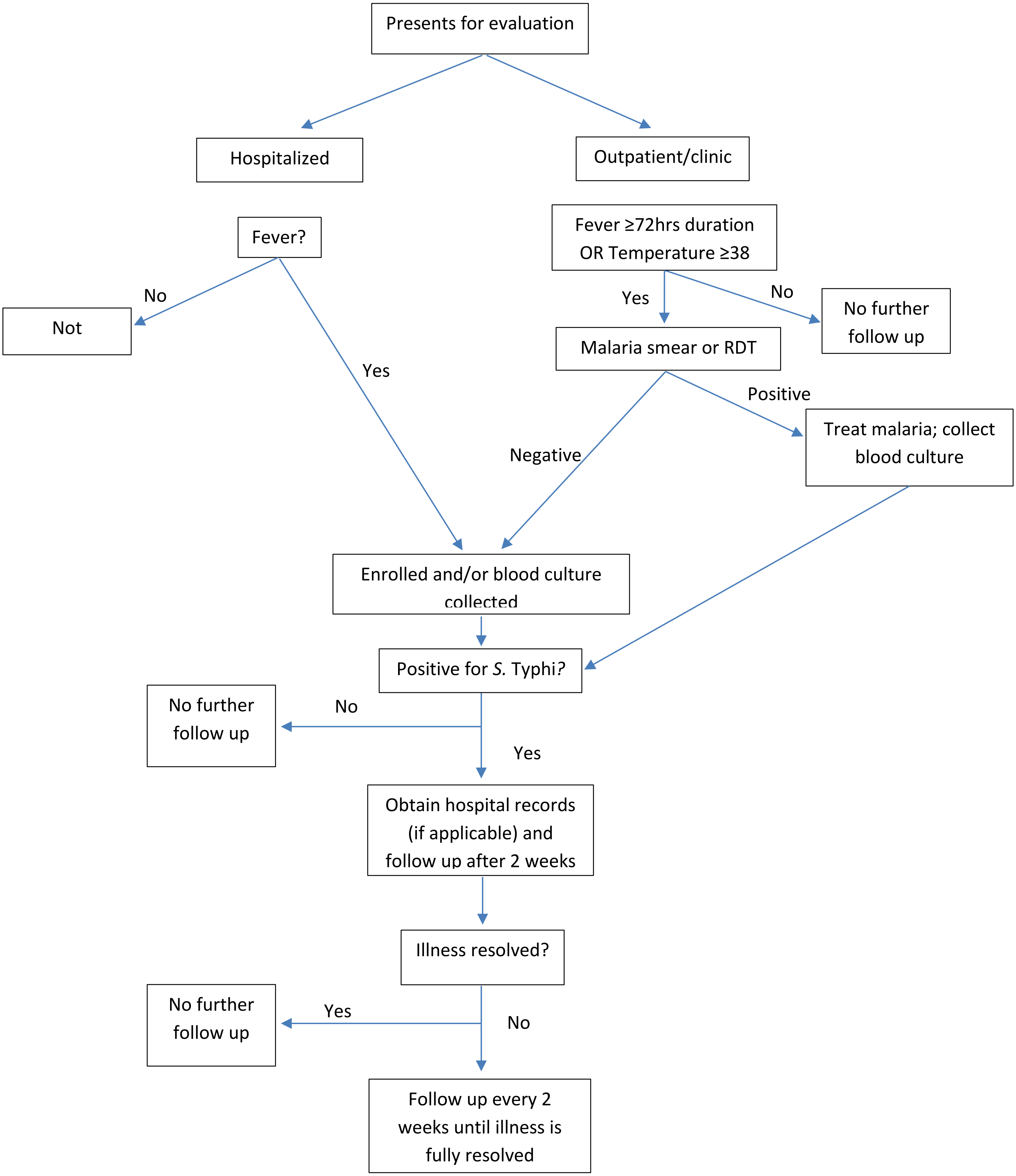
Presents for evaluation
Hospitalized
Outpatient/clinic
Fever?
No
Not
Yes
Fever ≥72hrs duration OR Temperature ≥38
Yes
No
No further follow up
Malaria smear or RDT
Positive
Treat malaria; collect blood culture
Negative
Enrolled and/or blood culture collected
Positive for S. Typhi?
No
No further follow up
Yes
Obtain hospital records (if applicable) and follow up after 2 weeks
Illness resolved?
Yes
No further follow up
No
Follow up every 2 weeks until illness is fully resolved

8.1.1.1. Duration of Typhoid Fever

Participants with symptomatic, blood-culture-confirmed *S*. Typhi infection will be followed up every two weeks until their illness is completely resolved. Additional information on the illness will be recorded and included in their study records.

8.1.1.2. Hospital Records

For those patients who are hospitalized with fever and/or *S*. Typhi infections, their hospital records will be reviewed and relevant information transferred to study case report forms. Hospital records will be included in the participant's study records.

8.1.1.3. Comorbidity of Typhoid Fever and Malaria or other febrile illnesses

In some cases, participants may have co-infection of typhoid fever and malaria. These instances will be recorded and kept as part of a participant's study records. When information is available on other clinical diagnoses (e.g. pneumonia, meningitis or other invasive bacterial infections), this will be record on study case report forms.

8.2. Laboratory Evaluations

8.2.1. Clinical Laboratory Evaluations

Blood cultures will be used in this study to diagnose typhoid fever. Other laboratory assays will be used for research purposes to determine the immunogenicity of Vi-TCV as well as the noninterference of Vi-TCV and measles-rubella containing vaccine.

8.2.2. Blood Specimens

8.2.2.1. Collection of Clinical Specimens

All individuals who meet the specimen collection criteria will have blood drawn for bacterial blood culture and malaria rapid test. For participants whose parent/caregiver consents to the additional antibody kinetics substudy, an EDTA sample of 3-5ml will be collected. Subjects in the Immunogenicity and Reactogenicity Substudy will have 5 mL blood drawn before vaccination and at days 28 and 730 after vaccination to determine vaccine immunogenicity as described above. Due to the study interruption because of the outbreak of COVID-19, EDTA samples will be collected until the final study visit – up to day 1095 for participants. Subjects in groups 1-3 in the HIV-exposed Vaccine Safety and Immunogenicity Substudy will have 5-10 mL blood drawn before vaccination for testing presence of HIV RNA and baseline immunogenicity testing, and 28 days after each vaccination date (9 months and 15 months) to determine vaccine immunogenicity as previously described. Participants enrolled in group 4 will have sample taken for immunogenicity testing at 9, 10, 15 and 16 months. HIV testing for mothers of potential participants in group 4 is mentioned in section 8.2.3.6.

8.2.2.2. Conditions for Handling, Transport and Storage of Clinical Specimens

Blood samples drawn for diagnosis and confirmation of suspected typhoid fever will be handled, stored, processed, in accordance with standard procedures of MLW Microbiology Laboratory.

Blood samples taken for the immunogenicity study will be transported to MLW research laboratories daily where they will be processed by trained study staff in the laboratory before storage at -20°C. For children receiving TCV or MCV-A and measles-rubella vaccination at the same time, samples will be split so that one set of samples can be retained at MLW for typhoid antibody and functional immunity testing, and a second set of samples can be shipped to the University of Maryland laboratory in Baltimore, MD for performance of measles and rubella antibody assays that are unavailable in Malawi.

8.2.3. Assays

8.2.3.1. Anti-Vi IgG Antibody ELISA

The primary laboratory technique performed will be anti-Vi IgG antibody ELISA performed on the extracted plasma sample, using a commercially available assay (Vacczyme Binding Site, or other comparable assay). This assay will be performed according to the manufacturer's instructions on-site at MLW.

8.2.3.2. Measles PRN Assay

The PRN assay measures the ability of measles-specific antibodies to neutralize the macroscopic cytopathology caused by a single virus particle on a monolayer of Vero cells. Serum is serially diluted until antibodies become insufficient to neutralize the virus. An end-point is defined as the inverse of the serum dilution that reduces the number of plaques by at least 50%. Titers are reported in mIU/ml by comparison with an existing, calibrated standard.

8.2.3.3. Measles ELISA

Measles-specific antibody levels can be quantified by indirect ELISA. Serially diluted samples are added to 96-well microtiter plates coated with measles virus lysates, and bound antibodies are detected with horse-radish peroxidase (HRP)-labeled anti-human antibodies.

8.2.3.4. Rubella PRN Assay

Similar to above measles PRN assay.

8.2.3.5. Rubella ELISA

Rubella-specific antibody levels can be quantified by indirect ELISA. Serially diluted samples are added to 96-well microtiter plates coated with measles virus lysates, and bound antibodies are detected with labeled anti-human antibodies.

8.2.3.6. HIV testing

HIV-exposed participants in the HIV-exposed substudy (n=up to 225) will be tested for the presence of HIV RNA. Mothers of potential participants recruited to the HIV unexposed cohort in the HIV-exposed substudy (n=100) who do not have a negative HIV test done and recorded within the last 3 months will be tested for HIV using a rapid HIV test or any licensed ELISA test kit that is confirmed by a second rapid HIV test by a different method, a repeat ELISA, or a Western blot or plasma HIV-1 RNA. Individuals who test positive will be counseled and referred to the Ndirande HIV clinic, located on the grounds of the Ndirande Health Centre.

9. Assessment of Safety

9.1. Methods and Timing for Assessing Safety Parameters

9.1.1. Post-Vaccination

9.1.1.1. Immediate Reactions

All participants will be observed for immediate reactions for 30 minutes after vaccination, with appropriate medical treatment readily available in case of an anaphylactic reaction following the administration of study vaccine. Immediate reactions will be assessed by a study physician or appropriately trained medical staff. All reactions that occur during this time will be recorded on the CRF. Any immediate reaction which meets the criteria for a serious adverse event must also be documented on an SAE form.

9.1.1.2. Solicited Local and Systemic Reactions

Participants in the immunogenicity and reactogenicity substudy will be assessed for local and systemic reactions through 7 days post-vaccination with study vaccine. Using a standardized data collection instrument the following local and systemic reactions will be documented and be graded on predefined scales based on functional assessment or magnitude of reaction: fever, injection site pain, swelling, tenderness, erythema; myalgia; arthralgia.

9.1.2. Note on Timing

In the immunogenicity and reactogenicity substudy, all solicited and unsolicited adverse events occurring through Day 7 post-vaccination will be assessed and recorded. All adverse events that meet the criteria for a Serious Adverse Event and that occur through six months post-vaccination will be assessed and recorded.

9.1.3. Definition of Adverse Events

Adverse event	Any untoward medical occurrence in a participant to whom Vi-TCV or MCV-A has been administered, including occurrences that are not necessarily caused by or related to the vaccine.

	Any untoward and unintended occurrence in a participant who has received Vi-TCV or MCV-A which is related to that vaccine
Serious adverse event	Any untoward medical occurrence that: results in death; is life-threatening; requires inpatient hospitalization or prolongation of existing hospitalization; results in persistent or significant disability.<br>Other important medical events may be considered serious if the event jeopardizes the participant or requires intervention to prevent one of the above consequences.
Suspected Unexpected Serious Adverse Reaction (SUSAR)	A serious adverse reaction, the nature and severity of which is not consistent with the information about the vaccine described in the Investigator's Brochure, that is related to the vaccine

9.2. Reporting Procedures

9.2.1. Adverse Events

All adverse events related to receipt of vaccine that are reported by telephone or home or clinic visits in the first 7 days after vaccination will be recorded, including a description of the event, date of onset and resolution, severity, assessment of relatedness to the study, and action taken.

9.2.2. Serious Adverse Events

Life-threatening SAEs or death must be reported via telephone or email to the Sponsor and the NHSRC and PMPB within 3 business days after notification of the event. All SAEs will be reported to the Institutional Review Board and the manufacturer. Related and unexpected deaths will be reported within 24 hours.

9.3. Data and Safety Monitoring Board

An independent Data and Safety Monitoring Board including physicians and a statistician will be established. This committee will meet before the study starts and will hold regular meetings every six months for the duration of the trial. An ad hoc meeting of the DSMB may be called at any time by the DSMB Chair, funder, sponsor or investigator if imminent participant safety issues arise. If a significant safety concerns arise during the study, the DSMB Chair may convene a meeting to review safety and any other aspects of the study. Significant safety events may include, but are not limited to:

- A death or life-threatening condition sustained by a participant, regardless of causality.
- An unexpected serious safety issue newly identified during the development program that could expose participants to unnecessary risks.
- Any other concern regarding participant safety raised by any DSMB member, funder, investigator or sponsor.

Proposed study amendments that significantly alter the treatment plan and/or deal with participant safety concerns will prompt an ad hoc meeting of the DSMB for review prior to implementation of changes. This may require suspension of enrollment pending DSMB review.

10. Monitoring

10.1. Monitoring Plan

10.1.1. Site Initiation Visit

A team from the sponsor, CVD, will visit the site prior to the start of the study to discuss the protocol and data collection procedures with site personnel. Prior to enrollment of subjects at the study site, specific regulatory documents must be available, such as IRB required approvals, and curriculum vitae for investigator and study staff. The sponsor will inform the investigator which documents need to be provided according to the applicable regulatory requirements.

10.1.2. Follow Up Visits

Monitoring will be conducted according to the sponsor's requirements. The individuals responsible for monitoring the study will periodically review the progress of the study and should have access to all records necessary to ensure the ethical and safety conduct of the study and the integrity/validity of the recorded data.

During site visits and contacts, the monitor will:

- Check and assess the progress of the study.
- Review study data collected.
- Perform source data verification.
- Review regulatory files.
- Identify any issues and address their resolution.

This will be done in order to verify that:

- The data are authentic, accurate and complete.
- The safety and rights of subjects are being protected.
- The study is conducted in accordance with the approved protocol (and any subsequent amendment) and all applicable regulatory requirements.

As part of study conduct, the principal investigator agrees to allow the monitor or sponsor representative direct access to all relevant documents and to allocate his/her time and the time of his/her staff to the monitor to discuss findings and any relevant issues.

10.1.3. Close-Out Visit

Upon completion of the study, the study sponsor will conduct the following activities:

- Data clarification and/or resolution
- Return or destruction of study vaccine
- Destruction of unused stored samples for which participants have not consented storage
- Review of site study records for completeness

10.2. Inspections

For the purpose of compliance with applicable regulatory guidelines it may be necessary for the sponsor or national or foreign regulatory authorities to conduct a site audit. This may occur at any time from start to after conclusion of the study.

The investigator agrees to allow the auditor direct access to all relevant documents and to allocate his/her time and the time of his/her staff to the auditor to discuss findings and any relevant issues.

National and foreign regulatory authorities may conduct a regulatory inspection of this study. If a regulatory authority requests an inspection, the investigator must inform the sponsor immediately about this request. The investigator agrees to allow the inspector(s) direct access to all relevant documents and to allocate his/her time and the time of his/her staff to the inspector(s) to discuss findings and any relevant issues.

10.3. Archiving

The principal investigator will maintain all records pertaining to this study for at least 2 years following the date a marketing application is approved for the vaccine in Malawi, or if no application is to be filed or if the application is not approved, until 5 years following completion of clinical study report.

11. Statistical Considerations

11.1. Study Hypotheses

11.1.1. Primary Study Hypothesis

The primary study hypothesis is that the Vi-TCV vaccine is efficacious in preventing typhoid fever, confirmed by blood culture. We will test the one-sided null hypothesis H_0: VE ≤ 0, where VE is vaccine efficacy. The primary endpoint is clinical typhoid fever, confirmed by blood culture, with cases identified by passive surveillance in hospital and clinics.

11.1.2. Secondary Study Hypotheses

The secondary study hypothesis is that the safety profile of the Vi-TCV vaccine is adequate.

11.2. Definition of Analysis Sets

11.2.1. Intention-to-Treat Population

The intention-to-treat (ITT) population includes all randomized individuals, in the vaccine groups to which they were assigned, and all follow-up time and cases beginning with the time of randomization. The primary analysis will be based on this population.

11.2.2. Per-Protocol Population

The per-protocol (PP) population includes all vaccinated individuals and follow-up time beginning 14 days after vaccination. Typhoid fever cases occurring before 14 days after vaccination will not be included in analysis of the PP population.

11.3. Analysis Plan

11.3.1. Efficacy

Vaccine efficacy will be evaluated based on cases accrued at three timepoints: on through April 3, 2020 when all participants have been followed for a minimum of 18 months; through September 30, 2021, the end date for the analysis including all subgroups, when all participants have been followed for a minimum of 36 months; and until the end of the extended surveillance period, through December 31, 2021. Subgroups analyses will be conducted to calculate and compare VE by age group (<2 vs. 2-<5 vs. 5+ and <5 vs. 5+). If there is a minimum of 30 symptomatic, blood-culture confirmed *S*. Typhi infection cases in each age subgroup, then this will be done at 18 months (through April 3, 2020) surveillance endpoint. If not, this age group analysis will be done at the end of the September 2021 surveillance period, regardless of number of cases in each age group. The third analysis will likewise include all subgroup analyses.

The primary analysis to test the hypothesis that the Vi-TCV vaccine is efficacious will be an intention-to-treat (ITT) analysis, which will include all first episodes of laboratory-confirmed typhoid fever occurring after vaccination.

VE is defined as the reduction in incidence rates of first episodes of culture-confirmed typhoid fever:

VE= (1- incidence rate in Vi-TCV vaccines / incidence rate in MCV-A vaccinees) x 100%,

where in each vaccine group the incidence rate is defined as (number of first episodes of culture-confirmed typhoid fever) / (total follow-up time), and follow-up time for each individual is the smallest of (time to first episode of typhoid fever, time to withdrawal or loss to follow-up, and end of follow-up).

We assume the number of first episodes of culture-confirmed typhoid fever follows a Poisson distribution in each of the Vi-TCV and MCV-A groups and define x_1 and x_2 as the numbers of

first episodes in recipients of Vi-TCV and MCV-A vaccines, respectively. Then the number of first episodes in Vi-TCV recipients, conditional on the total first episodes $X = x_1 + x_2$, follows a binomial distribution with parameters X and $P = x_1 / (x_1 + x_2)$. Now letting h = (total follow-up time among MCV-A recipients) / (total follow-up time among Vi-TCV recipients), we have

$$VE = (1 – R) \times 100\%, \text{ where } R = hP / (1-P).$$

Then we can test the null hypothesis that the vaccine has no protective efficacy, H_0: VE ≤ 0, by testing the equivalent hypothesis that $P \geq P_0$, where $P_0 = 1 / (h+1)$; we expect h to be approximately 1 and P_0 to be approximately 0.5.

A two-sided 95% confidence interval (CI) for VE is calculated by first obtaining an exact two-sided 95% CI for P and then transforming the limits of that interval, using the relationship

$$VE = 1 – hP / (1-P) \times 100\%.$$

The null hypothesis can be tested by an exact one-sided p-value; $p < 0.025$ will indicate statistically significant efficacy. Equivalently, significant efficacy will be indicated by a lower limit > 0 for the 95% CI for VE.

If any randomized study participants fail to receive vaccine or do not receive the vaccine to which they were assigned, or if any are lost to follow-up immediately after vaccination, a modified ITT analysis will be done, in which subjects will be analyzed according to the vaccine received and any who were not vaccinated or provided no follow-up after vaccination will be deleted from the analysis,

VE will also be assessed in a per protocol (PP) analysis, in which the test of significant efficacy and the CI for VE will be just as in the primary (ITT) analysis, except that only first episodes of culture-confirmed typhoid fever occurring at least 14 days after vaccination will be included.

No formal interim analysis of vaccine efficacy is planned.

If at least 5 subjects have more than one episode of culture-confirmed typhoid fever, then an analysis of vaccine efficacy against all episodes will be done. In this analysis, a Poisson model will be used and the vaccine efficacy calculated as (1-IDR) x 100%, where IDR is the incidence density ratio (incidence density for Vi-TCV divided by incidence density to MCV-A). An offset for the length of follow-up will be included. In this analysis, all available follow-up will be used, and all episodes of culture-confirmed typhoid fever with onset after vaccination will be included.

Secondary analyses will adjust efficacy for age at vaccination and sex. Subgroups will include children < 5 years (<2 vs. 2-5 if there are a minimum of 30 symptomatic, blood-culture confirmed S.Typhi infection cases in each age subgroup) and ≥ 5 years and male and female.

11.3.2. Safety

To evaluate safety, proportions of subjects in the Vi-TCV and MCV-A groups experiencing any category of adverse event (AE) will be calculated, with corresponding exact 95% confidence intervals. In addition, the difference in proportions (Vi-TCV – MCV-A), with 95% confidence intervals will be calculated, as well as one-sided p-value for the null hypothesis of more adverse events in Vi-TCV recipients using Fisher's exact test.

The vaccine groups will also be compared with respect to SAEs occurring through one month and six months post vaccination, listed by relationship to vaccination (related or not related). As with all AEs, SAEs will be compared using a one-sided Fisher exact test.

11.3.3. Additional outcomes

Details on statistical analysis for additional outcomes will be provided in the Statistical Analysis Plan.

11.4. Sample Size and Power Considerations

The null hypothesis can be tested by an exact one-sided p-value; $p < 0.025$ will indicate statistically significant efficacy. Equivalently, significant efficacy will be indicated by a lower limit > 0 for the 95% CI for VE. With assumption of 80% vaccine efficacy, the minimum number of cases needed to evaluate the primary outcome with 90% power is 30. With 28,500 children enrolled, this is likely to be achieved with an average of 24 months follow-up per participant. Lower efficacy, fewer children enrolled, or a lower than assumed incidence will require longer follow-up to reach the minimum number of cases. (See tables 2 and 3). Tables below also show cases required for lower limit >30 for the 95% CI for VE. Currently, our study has exceeded the prespecified number of cases (i.e., n=30) for the primary outcome.

We have also calculated the study power based on a 10% decrease in incidence in the control group. The null hypothesis can be tested by an exact one-sided p-value; $p < 0.025$ will indicate statistically significant efficacy. Equivalently, significant efficacy will be indicated by a lower limit > 0 for the 95% CI for VE. With assumption of 80% vaccine efficacy, the minimum number of cases needed to evaluate the primary outcome with 90% power is 30. With 28,500 children enrolled, at an assumed incidence of 90/100,000 (0.0009 per year) this is likely to be achieved with an average of 24 months follow-up per participant. Lower efficacy, fewer children enrolled, a greater loss to follow-up, or a lower than assumed incidence will require longer follow-up to reach the minimum number of cases. (See tables 2b and 3b). Tables below also show cases required for lower limit >30 for the 95% CI for VE.

Table 2a. Cases and total subjects required for at least 90% power to reject the null hypothesis of vaccine efficacy (VE) ≤0 or ≥30% (equivalently, obtain a two-sided 95% confidence interval for VE with lower bound greater than 0 or 30%, 2-year follow-up

Assumptions: Equal numbers of subjects and equal total follow-up time in groups of subjects receiving typhoid conjugate vaccine and control treatment, attack rate 0.001 per year in unvaccinated individuals, type error rate (α) = 2.5%, one-sided , **each subject followed for two years**

	Null hypothesis: VE ≤ 0%					Null hypothesis: VE ≤ 30%				
	Proportion of cases in vaccinated subjects					Proportion of cases in vaccinated subjects				
**VE under alternative hypothesis**	**Under null hypothesis**	**Under alternative hypothesis**	**Number of cases required for 90% power**	**Total subjects (vaccine and control) expected to result in required number of cases**	**Total subjects, allowing for 15% loss to follow-up**	**Under null hypothesis**	**Under alternative hypothesis**	**Number of cases required for 90% power**	**Total subjects (vaccine and control) expected to result in required number of cases**	**Total subjects, allowing for 15% loss to follow-up**
75%	0.5	0.20000	30	24000	28236	0.41176	0.20000	55	44000	51766
80%	0.5	0.16667	23	19168	22552	0.41176	0.16667	41	34168	40198
85%	0.5	0.13043	17	14784	17394	0.41176	0.13043	28	24348	28646
90%	0.5	0.09091	15	13638	16046	0.41176	0.09091	22	20000	23530

Table 2b. Cases and total subjects required for at least 90% power to reject the null hypotheses of vaccine efficacy (VE) ≤ 0 or ≤ 30%
(equivalently, obtain a two-sided 95% confidence interval for VE with lower bound greater than 0 or 30%), 24-month follow-up

Assumptions: Equal numbers of subjects and equal total follow-up time in groups of subjects receiving typhoid conjugate vaccine and control treatment, attack rate **0.0009** per year in unvaccinated individuals, type error rate (α) = 2.5%, one-sided, **each subject followed for 24 months**

	Null hypothesis: VE ≤ 0%					Null hypothesis: VE ≤ 30%				
	Proportion of cases in vaccinated subjects					Proportion of cases in vaccinated subjects				
**VE under alternative hypothesis**	**Under null hypothesis**	**Under alternative hypothesis**	**Number of cases required for 90% power**	**Total subjects (vaccine and control) expected to result in required number of cases**	**Total subjects, allowing for 15% loss to follow-up**	**Under null hypothesis**	**Under alternative hypothesis**	**Number of cases required for 90% power**	**Total subjects (vaccine and control) expected to result in required number of cases**	**Total subjects, allowing for 15% loss to follow-up**
75%	0.5	0.20000	30	26668	31376	0.41176	0.20000	55	48890	57518
80%	0.5	0.16667	23	21298	25058	0.41176	0.16667	41	37964	44664
85%	0.5	0.13043	17	16426	19326	0.41176	0.13043	28	27054	31830
90%	0.5	0.09091	15	15152	17826	0.41176	0.09091	22	22224	26146

Table 3a. Cases and total subjects required for at least 90% power to reject the null hypotheses of vaccine efficacy (VE) ≤ 0 or ≤ 30% (equivalently, obtain a two-sided 95% confidence interval for VE with lower bound greater than 0 or 30%), 2.5 year follow-up

Assumptions: Equal numbers of subjects and equal total follow-up time in groups of subjects receiving typhoid conjugate vaccine and control treatment, attack rate 0.001 per year in unvaccinated individuals, type error rate (α) = 2.5%, one-sided, **each subject followed for 2.5 years**

	Null hypothesis: VE ≤ 0%					Null hypothesis: VE ≤ 30%				
	Proportion of cases in vaccinated subjects					Proportion of cases in vaccinated subjects				
VE under alternative hypothesis	Under null hypothesis	Under alternative hypothesis	Number of cases required for 90% power	Total subjects (vaccine and control) expected to result in required number of cases	Total subjects, allowing for 15% loss to follow-up	Under null hypothesis	Under alternative hypothesis	Number of cases required for 90% power	Total subjects (vaccine and control) expected to result in required number of cases	Total subjects, allowing for 15% loss to follow-up
75%	0.5	0.20000	30	19200	22590	0.41176	0.20000	55	35200	41412
80%	0.5	0.16667	23	15334	18040	0.41176	0.16667	41	27334	32158
85%	0.5	0.13043	17	11828	13916	0.41176	0.13043	28	19480	22918
90%	0.5	0.09091	15	10910	12836	0.41176	0.09091	22	16000	18824

Table 3b. Cases and total subjects required for at least 90% power to reject the null hypotheses of vaccine efficacy (VE) ≤ 0 or ≤ 30%
(equivalently, obtain a two-sided 95% confidence interval for VE with lower bound greater than 0 or 30%), 30-month follow-up

Assumptions: Equal numbers of subjects and equal total follow-up time in groups of subjects receiving typhoid conjugate vaccine and control treatment, attack rate **0.0009** per year in unvaccinated individuals, type error rate (α) = 2.5%, one-sided, **each subject followed for 30 months**

	Null hypothesis: VE ≤ 0%					Null hypothesis: VE ≤ 30%				
	Proportion of cases in vaccinated subjects					Proportion of cases in vaccinated subjects				
VE under alternative hypothesis	Under null hypothesis	Under alternative hypothesis	Number of cases required for 90% power	Total subjects (vaccine and control) expected to result in required number of cases	Total subjects, allowing for 15% loss to follow-up	Under null hypothesis	Under alternative hypothesis	Number of cases required for 90% power	Total subjects (vaccine and control) expected to result in required number of cases	Total subjects, allowing for 15% loss to follow-up
75%	0.5	0.20000	30	21334	25100	0.41176	0.20000	55	39112	46016
80%	0.5	0.16667	23	17038	20046	0.41176	0.16667	41	30372	35732
85%	0.5	0.13043	17	13142	15462	0.41176	0.13043	28	21644	25464
90%	0.5	0.09091	15	12122	14262	0.41176	0.09091	22	17778	20916

12. Quality Control and Quality Assurance

12.1. General

The study will be conducted in accordance with the procedures specified in the protocol and staff will be guided by the study manual of procedures (MoP). Study data collection forms are designed to guide staff study conduct; forms also include areas for documenting that activities did occur (even if these activities did not require recording of data) and in the appropriate sequence. All study staff, including temporary staff such as nurses and interviewers, must attend mandatory training prior to participant enrollment.

Individual SOPs will be developed and documented for key study procedures and refined/revised as necessary. These SOPs will be included in the study MoP at the site or in the laboratory.

Site monitoring will be conducted to ensure that human subject protection procedures and study procedures, including study vaccine administration and clinical data and biological specimen collection, are of high quality and that the study is conducted in accordance with the protocol.

After data have been entered in the study database, they will be checked systematically by data management staff according to a pre-specified data validation plan. All listings of the database will be reviewed and discussed for assessment of consistency and medical plausibility. After resolution of all issues, the Statistical Analysis Plan (SAP) will be finalized and the database will be locked after resolution of any remaining queries. An audit trail will be kept of all subsequent changes to the data.

12.2. Trainings

Trainings for the research team (including the investigator, project manager/study coordinator, researchers, physicians, nurses, and technicians) participating in the clinical trial will include: the basics of research and ethics, information on how to conduct the trial, the standard operation procedures (SOPs) to be used in the trial, and the procedures for study vaccine management and use.

13. Ethics and Human Subjects Protections

13.1. Ethics Standards

The study will be conducted in accordance with the principles of the Declaration of Helsinki and in accordance with principles of Good Clinical Practice.

13.2. Institutional Review Board(s)

This protocol, informed consent document, proposed advertising material, and participant information sheet will be submitted to the University of Maryland Institutional Review Board, the

University of Liverpool (UoL) Ethical Review Board, and the Malawi National Health and Science Review Committee (NHSRC) and appropriate local research ethics committee in Malawi for written approval. Reports will be provided to the University of Maryland IRB, University of Liverpool IRB, and NHSRC annually. Amendments will also be provided to and approved by these parties.

13.3. Recruitment

13.4. Informed Consent Process

This process will include providing basic information in the local language about the purposes, eligibility, procedures, risks, and visits schedule of the trial. Parents will be encouraged to ask questions and answers will be provided as needed. This information will include the purpose of the trial, what will be asked of the participant and family, the side effects of vaccination and other risks of the study, blood draw and storage of blood. The voluntary nature of the study will be emphasized and the right to withdraw for any reason without prejudice will be explained.

The consent form will be signed or a thumbprint provided by the child's parent or guardian before any procedures are conducted. Assent will be obtained from children age 8 and over. The consent/assent form will be dated. A copy will be given to the participant or his/her guardian and a copy will be stored at the study site.

If a parent or guardian is unable to read or sign the consent form, an impartial witness will assist for the entire informed consent process. The parent or guardian will provide a thumbprint, and the impartial witness will sign the consent form, thereby confirming that all information was provided and consent was freely given. The impartial witness can be a family or community member who is independent from the study team.

13.5. Subject Confidentiality

The study records will be stored in a locked office or in a password-protected secure database. No information concerning the study or the data will be released to any unauthorized third party.

The study monitor or other authorized representatives of the sponsor may inspect all documents and records.

13.6. Study Discontinuation

Study discontinuation is not expected to occur. However, if the study is discontinued for safety reasons, parents of participants will be informed of the reasons for discontinuation and of the implications/potential consequences for the child. The study and its team will provide care for any medical needs of a participant as a result of the conduct of this study.

13.7. Sharing Study Results

When the clinical study report is completed, the investigators will share the summary results with the participating communities. Community meetings will be held to share the results of the study and to allow for questions and answers. It is anticipated that the results of this trial may have a significant bearing on policy decisions regarding the licensure of typhoid conjugate vaccines in Malawi and other countries in Africa.

13.8. Use of Stored Specimens

Biological specimens will be maintained until the end of the study. This is to allow time for all study-related testing. The specimens will be maintained at the MLW in Blantyre, Malawi and the University of Maryland CVD. No personal-identifying information associated with the study will be stored on site with the specimens. Following completion of the study, all specimens will be kept in a secure location for 5 years. If, prior to destruction, future unrelated studies are proposed for the specimens, such studies will be initiated with appropriate ethics committee approvals, and, if deemed appropriate by such committees, informed consent from participants.

13.9. Compensation

Participants in vaccine study to receive no financial compensation, but benefits as above. Participants in immunogenicity substudies and mothers who consent to HIV testing in the HIV exposed substudy to receive the currently-recommended compensation for time and trouble (approximately MK 1500, $1.5-2 per visit). Mothers and children (group 4) in the HIV exposed vaccine safety and immunogenicity substudy will receive reimbursement as a single study encounter. From 2019, this reimbursement is updated according to recent National consensus recommendations, to approximately USD $10 per visit (approximately MK 7200).

14. Data Handling and Record Keeping

14.1. Source Data and Access to Data

Research records generated in this study will be stored in a locked room and on a secure electronic database. Only authorized personnel will have access to these data. Authorized representatives of regulatory agencies may examine clinical records for the purposes of quality assurances reviews, audits, and evaluation of the study safety and progress.

14.2. Data Recording and Recordkeeping

Data (safety and immune response data) will be recorded on paper case report forms for scanning into an electronic data base or entered directly into an electronic data based. Case report forms designed specifically to capture data for this study will be generated. The study records will be stored in a locked office or in a password-protected secure database. The study records will be stored by the principal investigator for 5 years following the termination of the study.

14.3. Procedures for Reporting Protocol Deviations

Protocol deviations will be collected in a case report form and kept in the study record. Deviations that affect the scientific integrity of the study or the rights/safety of a volunteer will be reported to University of Maryland and local IRBs.

15. Publication Policy

The principal investigator and sponsor investigator will oversee collection, approvals, and dissemination of data from this study. All publications, including manuscripts, abstracts, oral/slide presentations, book chapters, etc. based on data collected in this study will be reviewed by each co-investigator before submission. Authors will acknowledge that the study was funded by the BMGF. In accordance with BMGF, all publications related to this study will be open access. Authorship will be determined in accordance with the ICMJE guidelines and other contributors will be acknowledged.

16. Literature References

1. Klugman, K.P., et al., *Immunogenicity, efficacy and serological correlate of protection of Salmonella typhi Vi capsular polysaccharide vaccine three years after immunization.* Vaccine, 1996. **14**(5): p. 435-8.
2. Koprowski, H., 2nd, et al., *Attenuated Shigella flexneri 2a vaccine strain CVD 1204 expressing colonization factor antigen I and mutant heat-labile enterotoxin of enterotoxigenic Escherichia coli.* Infect Immun, 2000. **68**(9): p. 4884-92.
3. Crump, J.A., S.P. Luby, and E.D. Mintz, *The global burden of typhoid fever.* Bulletin of the World Health Organization, 2004. **82**(5): p. 346-353.
4. Blum, L.S., et al., *Formative investigation of acceptability of typhoid vaccine during a typhoid fever outbreak in Neno District, Malawi.* Am J Trop Med Hyg, 2014. **91**(4): p. 729-37.
5. Kabwama, S.N., et al., *A large and persistent outbreak of typhoid fever caused by consuming contaminated water and street-vended beverages: Kampala, Uganda, January - June 2015.* BMC Public Health, 2017. **17**(1): p. 23.
6. Polonsky, J.A., et al., *Descriptive epidemiology of typhoid fever during an epidemic in Harare, Zimbabwe, 2012.* PLoS One, 2014. **9**(12): p. e114702.
7. Hendriksen, R.S., et al., *Genomic signature of multidrug-resistant Salmonella enterica serovar typhi isolates related to a massive outbreak in Zambia between 2010 and 2012.* J Clin Microbiol, 2015. **53**(1): p. 262-72.
8. Muyembe-Tamfum, J.J., et al., *An outbreak of peritonitis caused by multidrug-resistant Salmonella Typhi in Kinshasa, Democratic Republic of Congo.* Travel Med Infect Dis, 2009. **7**(1): p. 40-3.
9. Feasey, N.A., et al., *Three Epidemics of Invasive Multidrug-Resistant Salmonella Bloodstream Infection in Blantyre, Malawi, 1998-2014.* Clin Infect Dis, 2015. **61 Suppl 4**: p. S363-71.
10. Feasey, N.A., et al., *Rapid emergence of multidrug resistant, H58-lineage Salmonella typhi in Blantyre, Malawi.* PLoS Negl Trop Dis, 2015. **9**(4): p. e0003748.

11. Salerno-Goncalves, R., M.F. Pasetti, and M.B. Sztein, *Characterization of CD8(+) effector T cell responses in volunteers immunized with Salmonella enterica serovar Typhi strain Ty21a typhoid vaccine.* J Immunol, 2002. **169**(4): p. 2196-203.
12. Acharya, I.L., et al., *Prevention of typhoid fever in Nepal with the Vi capsular polysaccharide of Salmonella typhi. A preliminary report.* N Engl J Med, 1987. **317**(18): p. 1101-4.
13. Klugman, K.P., et al., *Protective activity of Vi capsular polysaccharide vaccine against typhoid fever.* Lancet, 1987. **2**(8569): p. 1165-9.
14. Yang, H.H., et al., *Efficacy trial of Vi polysaccharide vaccine against typhoid fever in south-western China.* Bull World Health Organ, 2001. **79**(7): p. 625-31.
15. Mohan, V.K., et al., *Safety and immunogenicity of a Vi polysaccharide-tetanus toxoid conjugate vaccine (Typbar-TCV) in healthy infants, children, and adults in typhoid endemic areas: a multicenter, 2-cohort, open-label, double-blind, randomized controlled phase 3 study.* Clin Infect Dis, 2015. **61**(3): p. 393-402.
16. Gibani, M.M., et al., *Control of Invasive Salmonella Disease in Africa: Is There a Role for Human Challenge Models?* Clin Infect Dis, 2015. **61 Suppl 4**: p. S266-71.
17. Jin, C., *Data from VAST trial - manuscript in progress.*
18. *Meningitis A nearly eliminated in Africa through Vaccination*. 2016 5/26/2017]; Available from: http://www.gavi.org/library/news/gavi-features/2016/meningitis-a-nearly-eliminated-in-africa-through-vaccination/.
19. Bharat Biotech International, L., *Package insert - Typbar TCV*.
20. WHO, *Weekly Epidemiological Record*. 2017, World Health Organization: Geneva, Switzerland. p. 13-20.
21. Ltd., S.I.o.I., *MenAfriVac package insert*. Pune, India.
22. Levine, M.M., et al., *Safety, infectivity, immunogenicity, and in vivo stability of two attenuated auxotrophic mutant strains of Salmonella typhi, 541Ty and 543Ty, as live oral vaccines in humans.* J Clin Invest, 1987. **79**(3): p. 888-902.
23. Shakya, M., et al., *Phase 3 Efficacy Analysis of a Typhoid Conjugate Vaccine Trial in Nepal.* N Engl J Med, 2019. **381**(23): p. 2209-2218.
24. Ruggeberg, J.U., et al., *Anaphylaxis: case definition and guidelines for data collection, analysis, and presentation of immunization safety data.* Vaccine, 2007. **25**(31): p. 5675-84.

Data and Safety Monitoring Board (DSMB) Charter

Accelerating Availability and Access to Typhoid Conjugate Vaccine funded by the Bill and Melinda Gates Foundation (OPP1151153).

C o n f i d e n t i a l

**Funding agency:**	Funded by **University of Maryland, Baltimore**, as part of a Bill and Melinda Gates Foundation (BMGF) grant for the **Typhoid Vaccine Acceleration Consortium (TyVAC)**
**Protocol Number:**	OPP1151153
**Contacts at UMB:**	Ken Simiyu<br>Project Director, TyVAC<br>University of Maryland School of Medicine<br>Institute for Global Health<br>685 W. Baltimore Street, Room 480<br>Baltimore, MD 21201
**Investigational Medicinal product:**	Vi polysaccharide-tetanus toxoid conjugate vaccine (Vi-TCV)<br>Licensed Trade name: Typbar-TCV®, Bharat-Biotech
**Charter prepared by:**	UMB
**Number of trials:**	3
**Countries:**	Nepal, Malawi, Bangladesh
**Trial designs:**	• Individual randomized controlled trial of Vi-TCV versus meningococcal group A conjugate vaccine in Nepal<br>• Individual randomized controlled trial of Vi-TCV versus meningococcal group A conjugate vaccine in Malawi<br>• Cluster randomized controlled trial of Vi-TCV versus Japanese encephalitis vaccine in Bangladesh
**Date of Charter:**	February 16 2018

University of Maryland Baltimore Signature Page

Data and Safety Monitoring Board (DSMB)
Charter
TyVAC Vaccine effectiveness studies

Reviewed and Accepted at Center for Vaccine Development
University of Maryland Baltimore School of Medicine by:

____ _____________________________
Ken Simiyu Date
Program Director, TyVAC

____ _____________________________
Stephen Peterson Date
Manager, Sponsored Programs Administration

University of Maryland Baltimore Signature Page 2

1 Introduction 4
2 Responsibilities of the DSMB 4
3 Organization and Interactions 4
4 DSMB Members 5
5 Confidentiality 6
6 Scheduling, Timing, and Organization of Meetings 7
7 DSMB Meetings 8
8 Reports of DSMB Deliberations 9
9 Study Halting Criteria 9
10 Statistical Monitoring Guidelines 9
11 Other Party Activities Related to DSMB According to Contract/ CTA SAE Reporting: 9

1 INTRODUCTION

An independent Data and Safety Monitoring Board (DSMB) will be convened to act in an advisory capacity to the University of Maryland (UMB) and the University of Oxford (UofO) (the study sponsors) to monitor participant safety, data quality, and evaluate the progress of the Vaccine impact studies within the Typhoid Vaccine Acceleration Consortium (TyVAC) Vaccine project. The Typhoid Vaccine Acceleration Consortium (TyVAC) is a multi-institutional project funded by the Bill and Melinda Gates Foundation, and led by the University of Maryland. The studies are designed to generate evidence on the impact of Vi-polysaccharide-tetanus toxoid conjugate vaccine (Vi-TCV) to support the use of Vi-TCV in countries with endemic typhoid.

The vaccine impact studies will be carried out in 3 separate trials in Nepal, Bangladesh, and Malawi. The aim of these studies is to measure the protective efficacy/effectiveness of Vi-TCV in preventing typhoid fever in a field setting. The variety of geographic settings, population genetics, and risk factors for disease will provide valuable information about the usefulness of Vi-TCV in diverse populations and epidemiologic settings. The study in Malawi is sponsored by the University of Maryland while those in Nepal and Bangladesh are sponsored by the University of Oxford (UofO) (See Appendix A).

The sponsors believe having one committee reviewing the studies would enhance the safety oversight for the 3 protocols, as the DSMB would be familiar with the products under study, the implementation of all three studies, and the cumulative safety information. Each site will have an Independent Safety Monitor (ISM). For Bangladesh, as per country regulations, a local DSMB will also be set up – the trial sponsor and the DSMB Coordinating Center (CC) will ensure that the Bangladesh DSMB charter is aligned to the international DSMB Charter.

There will be a Coordinating Center (CC) based at the University of Maryland School of Medicine (UMB) that shall provide the logistical management and support to the DSMB.

2 RESPONSIBILITIES OF THE DSMB

The DSMB is an independent group advisory to the sponsors and chief investigators. The primary objective of the DSMB is to monitor the safety of the intervention and the validity and integrity of the data from the clinical studies. Additionally, the DSMB will make recommendations to the study sponsors regarding the continuation, modification, or termination of any or all arms of the studies.

3 ORGANIZATION AND INTERACTIONS

The following chart illustrates the relationship between the DSMB and other entities in this study.

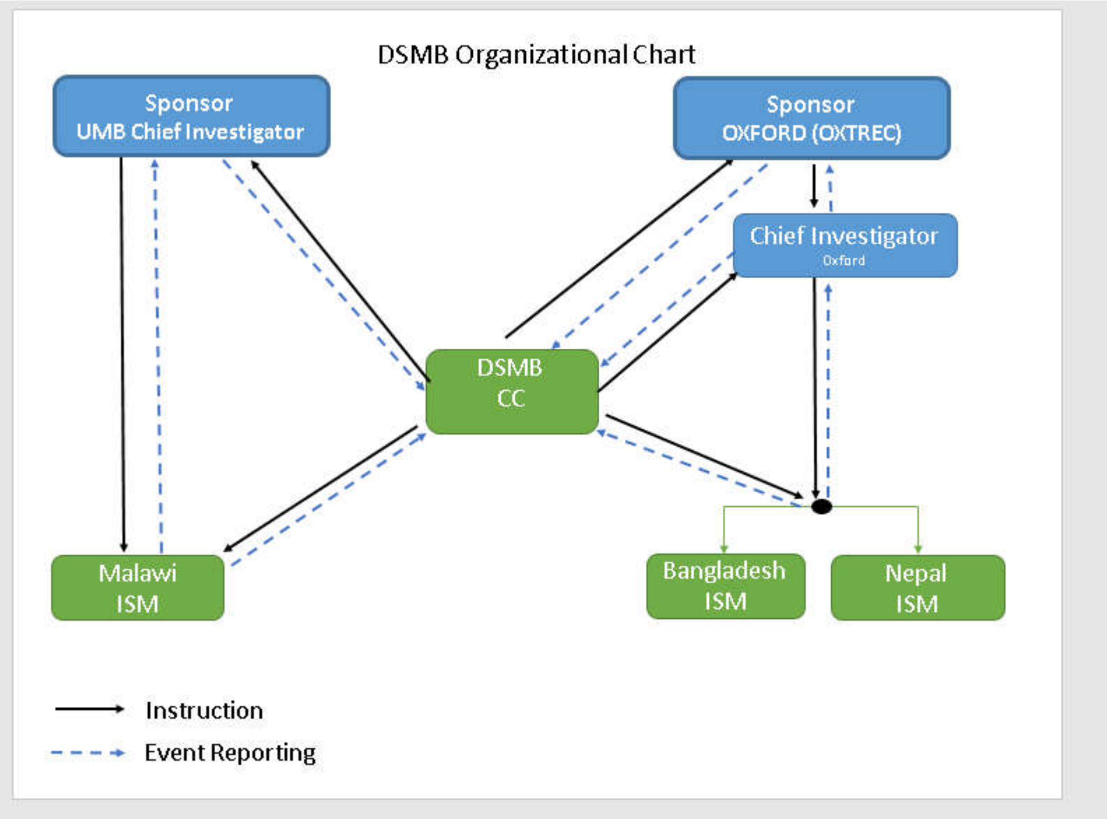

Communication with DSMB members will be primarily through the Sponsors and the Coordinating Center (CC) at the University of Maryland. UMB and UofO will be responsible for preparing tables with interim summaries of safety data from their respective trials and providing open session reports to the Coordinating Center (CC) for distribution to the DSMB at least 48 hours prior to scheduled meetings. It is expected that study investigators will not communicate with DSMB members about the study directly, except when making presentations or responding to questions at DSMB meetings or during conference calls.

4 DSMB MEMBERS

DSMB members and their expertise are listed in Appendix C. The DSMB will consist of 7 members. These will be a Chair, 3 member site representatives from Bangladesh, one member site representative from Malawi, one member site representative from Nepal and a biostatistician knowledgeable about statistical methods for clinical research and analysis of research data. The Coordinating Center (CC) working with the DSMB Chair, will be responsible for assuring the accuracy and timely transmission of final DSMB recommendations through meeting minutes recommending continuing the trialNo member of the DSMB may participate in this study as an investigator or otherwise be involved in any way in the conduct of the study.

Although DSMB members are expected to serve for the duration of the study, in the unlikely event that a member is unable to continue participation, the reason will be documented and a replacement member will be selected by the DSMB Chair in consultation with the investigator(s), funder and sponsor(s). The new member must have comparable expertise and qualifications to the DSMB member he/she is replacing.

4.1 Conflict of Interest

DSMB members will be volunteers. DSMB members should have no other relationship with the sponsor and/or investigator(s) that could impair the members' ability to objectively review study data as set forth below:

- DSMB members must not have any real or perceived scientific, financial, professional, personal, proprietary, or other conflict of interest related to the conduct, outcome, or impact of the study. This may include having been or being employed by the sponsor or investigator(s), having a fiduciary interest in the sponsor, conducting and/or managing the study, and/or having contact with participants during the course of regular clinical care;
- DSMB members must not be engaged in any simultaneously occurring competitive studies in any role that could pose a conflict of interest. DSMB members must also identify and disclose any concurrent service on other DSMBs of the same, related, or competing products.

All DSMB members must disclose all possible conflicts of interest in writing prior to beginning service as a DSMB member, and verbally prior to each meeting.

5 CONFIDENTIALITY

For purposes of this Agreement, the term "Confidential Information" means proprietary or nonpublic information, including information that is written, electronic, oral or visual, that is directly or indirectly furnished, provided to or disclosed by or on behalf of any Agreement Party ("Disclosing Party") to the other party ("Receiving Party"), including information relating to the Disclosing Party's business, operations or technologies.

With respect to any Confidential Information disclosed as part of this Charter Agreement, all materials, discussions, and proceedings of the DSMB are privileged and confidential. DSMB members agree to use this information exclusively to accomplish the responsibilities of the DSMB. No communication of the deliberations or recommendations of the DSMB, either written or oral, may occur except as required for the DSMB to fulfill its responsibilities. Individual DSMB members are expected to maintain confidentiality regarding the studies outside the DSMB (including, but not limited to the investigators, IRBs, or regulatory agencies) except as authorized by the Cordinating Centre or study Sponsors.

Members further agree to:

(a) Maintain Confidential Information in strict secrecy and confidence except as may be expressly authorized in this Agreement, using at least the same degree of care to prevent unauthorized disclosure of Confidential Information as it uses to protect its own information of like nature, and no less than a reasonable degree of care;

(b) Not make any use of the Confidential Information other than as described above without the express written consent of the Disclosing Party;

(c) Not reverse engineer, copy, disassemble or otherwise attempt to reconstruct any physical embodiments, samples or prototypes of such Confidential Information;
(d) Not incorporate, refer to or otherwise utilize all or any part of such Confidential Information to obtain any license or to apply for or to obtain any patent; and
(e) If so requested by the Disclosing Party, return or destroy, and cause its Representatives to return or destroy, any documents containing Confidential Information supplied by the Disclosing Party or any copies thereof or extracts there from made by the Receiving Party and any samples of materials supplied by the Disclosing Party, except for a single copy to be kept in the Receiving Party's confidential file to the extent required for the purpose of determining compliance with its obligations of confidentiality and limited use under this Agreement.

The parties agree that the obligations of confidentiality set forth above shall not apply to (a) Confidential Information which at the time of the disclosure is publicly available; (b) Confidential Information which after disclosure becomes publicly available other than as a result of any act or omission of the Receiving Party; (c) Confidential Information which the Receiving Party can prove to be lawfully in its possession at the time of disclosure and which was not acquired under a confidentiality obligation; (d) Confidential Information which is developed by or on behalf of the Receiving Party independently of any Confidential Information disclosed by the Disclosing Party; or (e) Confidential Information that the Receiving Party receives from a third party whose disclosure does not violate any confidentiality obligation.

6 SCHEDULING, TIMING, AND ORGANIZATION OF MEETINGS

Meetings will be generally conducted via teleconference with review materials provided, which will include a meeting agenda, the DSMB contact list and reports and documents required based on the objectives of the meeting. On occasion, DSMB may determine that a face-to-face meeting is required. Meetings and conference calls will be scheduled by the CC in collaboration with the sponsors. The DSMB will meet before the protocol begins and as needed or at least twice a year thereafter.

The first meeting will be an open session. The purpose of the first meeting is to review and finalize this Charter, to provide an overview of study activities, to review the protocol, and to review the schedule of meetings, points of decision-making, and statistical procedures for making recommendations regarding study continuation.

The Coordinating Centre will present the guidelines and strategy outlined in this Charter.
The following meetings are anticipated:

- To approve the Charter and DSMB procedures before enrollment begins
- To review safety data in the event a halting criteria is met
- To review the overall safety data for the study at study completion
- At any other time when the DSMB Chair or members think a meeting should be convened

The agenda for DSMB meetings and teleconferences will be drafted by the CC in consultation with the sponsors and finalized with the DSMB Chair. The agenda and meeting materials will be distributed at least 48 hours before each meeting or teleconference.

Before each meeting, when the agenda is sent out, the CC will ask all DSMB members to state whether they have developed any new conflicts of interest since the last formal annual report to sponsors. If a new conflict is reported, the Chair and sponsors will determine if the conflict limits the ability of the DSMB member to participate in the discussion. The DSMB will keep all interim study results strictly confidential.

It is expected that all DSMB members will attend every meeting and teleconference. However, it is recognized that this may not always be possible. A quorum of at least 4 DSMB members will be needed for DSMB deliberations; however for a quorum to be properly constituted there needs to be a site representative from at least 2 of the 3 sites. All standing DSMB members are voting members. The Board may also wish to decide in advance whether *ad hoc* members will be invited and their voting privileges.

7 DSMB MEETINGS

DSMB meetings and teleconferences will be organized into open, closed sessions and executive sessions.

- **Open Session**: During the open sessions, information will be presented to the DSMB by the sponsors, the chief investigators and the study staff as appropriate, with time for discussion. Information on the conduct of the trial including recruitment, compliance, data quality, and general operational issues will be discussed. Special precautions will be taken to ensure that the investigators and study staff will remain blinded to the specific results of interim analyses and individual treatment assignments.

- **Closed Session**: The closed session(s) will be attended by the DSMB members and an administrative person who isn't involved in the trialonly. The study statistician(s) will also attend to answer questions and present the data but will not be considered members of the DSMB. Closed sessions will be conducted separately for the Malawi trial and the Nepal/Bangladesh trials as the statisticians attending are not unblinded to all studies.

- **Executive Session**: The DSMB may elect to hold an **executive session** in which only the DSMB members are present in order to discuss study issues independently. DSMB members may be unblinded to individual treatment assignment should they consider it important for making recommendations for the study.

If a closed session or executive session occurs on a conference call, steps will be taken to ensure that only the appropriate participants are on the call, and to invite others to re-join the call only at the conclusion of the executive session.

At the conclusion of the closed and executive sessions, the DSMB Chair will provide a summary of the DSMB's recommendations to the sponsors, chief investigators and CC. This provides an opportunity for the sponsors Staff and the chief investigators to ask questions to clarify the recommendations. The DSMB Chair will take care to keep meeting participants appropriately blinded as described above. The meeting is then adjourned.

8 REPORTS OF DSMB DELIBERATIONS

- Initial summary: The DSMB CC is responsible for assuring the accuracy and transmission of a brief summary of the DSMB's discussion and recommendations for the sponsors and the chief investigators within 48 hours of the meeting or teleconference. The Chief investigators or designee will make the decision to approve or disapprove the recommendation(s), or request additional information. The final decisions regarding study conduct will be communicated to the sites investigators for implementation.

- Action plan: If the DSMB's recommendations require significant changes in study conduct or follow-up, the chief investigators will prepare an action plan outlining the steps required to implement the recommendations, or the rationale for not implementing the recommendations.

- Formal minutes: Open Session: The CC is responsible for the accuracy and transmission of the formal DSMB minutes for the open session, within 15 days of the meeting or teleconference. These minutes will summarize the key points of the discussion, requests for additional information, response to previous recommendations, and the recommendations from the current meeting. These minutes will be reviewed by the sponsors, the chief investigators and the CC before being forwarded to the DSMB Chair for final review and approval. The DSMB Chair may sign the minutes or indicate approval electronically via email. Subsequently, the meeting minutes will be sent back to the sponsors, the chief investigators and CC and will be included in the materials for the subsequent DSMB meeting to be approved by voice vote at that meeting. Once they have been voted and approved by the DSMB, they are considered Fina Closed session: minutes will be taken by the attending study statistician(s), approved by the DSMB and then saved in the confidential statistical trial master file until study closure.

9 STUDY HALTING CRITERIA

The study halting criteria detailed in Appendix B and the respective strudy protocols.

10 STATISTICAL MONITORING GUIDELINES

At the first DSMB meeting, review of the protocol will include review of the safety monitoring plan. The DSMB will discuss the statistical monitoring procedures they propose to follow to guide their recommendations about termination or continuation of the trial. The DSMB will utilize these procedures along with their clinical judgment at subsequent meetings to make recommendations regarding conduct of the study.

11 OTHER PARTY ACTIVITIES RELATED TO DSMB ACCORDING TO CONTRACT/ CTA SAE REPORTING:

12.1 SAE REPORTNG:

Per the CTA:

- For any SAE report that meets all of the following criteria of 1) Serious 2) Unexpected and 3) Suspected Adverse Reaction, the sponsors will provide to Bharat Biotech, Inc. by email, a completed SAE reporting form -
  Send to:
  kmohan@bharatbiotech.com

- As the manufacturer, Bharat-Biotech will, in a timely manner provide the sponsors and investigators with any information it now has or may obtain in the future regarding the safety and/or the toxicity of vaccine. The sponsors and investigators will promptly transmit that information to all Investigators.

12.2 DSMB MEETINGS, REPORTS AND RECOMMENDATIONS:

Per the CTA:

- The final Summary of Recommendations derived from the Closed sessions of the DSMB meetings will be provided to Bharat-Biotech by the DSMB secretariat.
  Email: kmohan@bharatbiotech.com

Appendix A: Synopsis of studies

The trials will be conducted at 3 sites as follows:

Site:	Nepal (Kathmandu)	Bangladesh (Dhaka)	Malawi (Blantyre)
**Protocol Number:**	OVG2017/05	XXXXXXX	XXXXXXX
**Sponsor:**	Oxford University	Oxford University	University of Maryland
**Investigators:**	Chief Investigator:<br>Andrew Pollard<br>Oxford Vaccine Group,<br>Department of Pediatrics,<br>University of Oxford	Chief Investigator:<br>Andrew Pollard<br>Oxford Vaccine Group,<br>Department of Pediatrics,<br>University of Oxford	Chief Investigator:<br>Kathleen Neuzil<br>Center for Vaccine<br>Development,<br>University of Maryland
	Principal Investigator:<br>Professor Buddha Basnyat<br>Oxford University Clinical<br>Research Unit Nepal – Patan<br>Academy of Health Sciences,<br>Kathmandu, Nepal	Principal Investigator:<br>John Clemens<br>icddr,b<br>Bangladesh	Principal Investigator:<br>Melita Gordon,<br>University of Liverpool,<br>Malawi.
**Study Phase:**	III	IV	III
**Trial design:**	Individually randomized controlled trial	Cluster randomized controlled trial	Individually randomized controlled trial
**Investigational Medicinal Product:**	Vi polysaccharide-tetanus toxoid conjugate vaccine (Vi-TCV)<br>Licensed Trade name: Typbar-TCV®, Bharat-Biotech		
**Formulation, Dose, Route of Administration**	A single 0.5ml vaccine dose which contains:<br>Purified Vi-Capsular Polysaccharide of *S.* Typhi Ty2 conjugated to Tetanus Toxoid 25µg<br>Sodium chloride 4.5 mg; Water for Injection q.s. to 0.5ml; Administration by Intramuscular injection; Pre-filled single-dose syringe		
Comparator (control)Treatment	Single dose Serogroup A meningococcal conjugate vaccine<br>Licensed trade name: MenAfriVac™, Serum Institute of India PVT. Ltd.<br>Dose 10µg for participants aged ≥1 year; 5µg for participants aged 9 to <12 months	Single dose Japanese Encephalitis<br>SA14-14-2 Japanese encephalitis vaccine<br>Lyophilized<br>Sterilized water for injection q.s. to 0.5ml<br>Licensed trade name: SA14-14-2 –Japanese Encephalitis Vaccine, Live<br>Administration by subcutaneous injection<br>Pre-filled multi dose	Single dose Serogroup A meningococcal conjugate vaccine<br>Licensed trade name: MenAfriVac™, Serum Institute of India PVT. Ltd.<br>Dose 10µg for participants aged ≥1 year; 5µg for participants aged 9 to <12 months
**Trial Participants:**	20000	43350	24000
**Participant Profile:**	Children 9 months to <16 years of age	Children 9 months to <16 years of age	Children 9 months through 12 years of age
**Study Duration:**	30 months	30 months	36 Months
**Follow-up duration:**	Two years follow-up, post vaccination, for each participant	Up to two years follow-up, post vaccination, for each participant	Two years follow-up, post vaccination, for each participant or until the number of verified cases is reached.

Appendix B: Study Halting Criteria

The safety oversight and proposed halting rules have been written to protect the safety to trial participants while acknowledging that these trials are being conducted in areas with high childhood morbidity and mortality. Serious adverse events and even deaths will occur with some frequency at the trial site. We have prior information on the safety of the vaccine through previous clinical trials, and the postmarketing surveillance in India, with over 3 million doses distributed.

The proposed halting criteria were discussed at the October 20, 2017 DSMB meeting. All SAEs will be reviewed and evaluated by a medically qualified individual for relationship to study vaccination. Any SAEs deemed related to study vaccination will be reviewed within 72 hours by the DSMB Chair.

If any of the following halting criteria are met, then the study will be suspended and further doses of vaccine will not be administered pending review of data by the DSMB.

Within 24 hours of receiving vaccine, any two subjects experience life-threatening anaphylactic * reaction related to vaccination.

- Within 7 days of vaccination, three or more subjects experience study-product-related SAEs within a single MedDRA category (e.g. gastrointestinal; respiratory; other infections and infestations).

Subsequent review of serious, unexpected and related AEs by the ISM, DSMB, ethics review committee, the sponsor(s) or other relevant local regulatory authorities may also result in suspension of further trials interventions/administration of study product at a site.

Anaphylaxis will be defined by Brighton Collaboration criteria. Anaphylaxis is a clinical syndrome characterized by sudden onset, rapid progression of signs and symptoms, major dermatologic involvement (generalized urticarial or erythema, angioedema, generalized pruritis with skin rash) AND major involvement of the cardiovascular system (measured hypotension and uncompensated shock) AND/OR the respiratory system (bronchospasm/stridor/upper airway swelling or respiratory distress with 2 or more of: tachypnea, recession, cyanosis, grunting, increased use of accessary muscles).

* Ruggeberg JU, Gold MS, Bayas JM, Blum MD, Bonhoeffer J, Friedlander S, et al. Anaphylaxis: case definition and guidelines for data collection, analysis and presentation of immunization safety data. Vaccine. 2007; 25:5675–84. [PubMed: 17448577]

Appendix C: DSMB Membership

DSMB Chair
Dr Roma Chilengi
Chief Scientific Officer/Head Enteric Disease & Vaccines Research Unit
Centre for Infectious Disease Research in Zambia (CIDRZ)
Plot 34620 Off Alick Nkhata Rd between ERB and FAZ, Mass Media, PO 34681 Lusaka, ZAMBIA
Cell +260973724935 roma.chilengi@cidrz.org

Voting Members

Nepal
Prof Dr Prakash Ghimire
Professor Microbiology ,
Central Department of Microbiology
Tribhuvan University , Kathmandu
Nepal
Tel 977 1 4331869
Personal 977 14488222
Email : prakashghimire@gmail.com

Bangladesh
Professor AKM Nurul Anwar
Former Director-General
Directorate General of Health Services
Government of the People's Republic of Bangladesh, and
Former Principal, Ibrahim Medical College
Res: Flat # 5B, House # 7, Road 4
Banani DOHS, Dhaka 1212
Cell: +881720018793
Res: +888753411
Email: prof.anwar1944@gmail.com

Professor Jalaluddin Ashraful Haq
Principal
Ibrahim Medical College
BIRDEM, Shahbagh, Dhaka
Res: House-18, Road-2, Block-E
Banasree, Rampura, Dhaka
Cell: +881731852966
Res: +88728 8255
Email: jahaq54@yahoo.com

Dr. Nur Haque Alam, MD
Senior Scientist, Nutrition & Clinical Services Division (NCSD),
icddrb,
Mohakhali, Dhaka, Bangladesh
Email: nhalam@icddrb.org
Tel: +8802 9827035

Malawi
Tisungane Knox Titus Mvalo MD
Pediatrician at Kamuzu Central Hospital, Malawi
Contact Mailing Address : Tidziwe Centre,
UNC Project,
Private Bag A-104,
Lilongwe,
Malawi.
tmvalo@unclilongwe.org
tisungemvalo@yahoo.com
Tel: +26599917355 or +265885024966

Statistician
Mary E Putt, PhD, ScD
Professor of Biostatistics
Department of Biostatistics, Epidemiology & Informatics
621 Blockley Hall
423 Guardian Drive
Perelman School of Medicine
University of Pennsylvania
Philadelphia, PA
19104
Tel: 215-573-7020
Email: mputt@pennmedicine.upenn.edu

CONSERVE Checklists

Use CONSERVE-CONSORT for completed trial reports and CONSERVE-SPIRIT for trial protocols.

**CONSERVE-CONSORT Extension: 8 April 2022**					
**Item**	**Item Title**	**Description**			**Page No.**
I.	Extenuating Circumstances	Describe the circumstances and how they constitute extenuating circumstances.			6
II.	Important Modifications	a. Describe how the modifications are important modifications.			6
		b. Describe the impacts and mitigating strategies, including their rationale and implications for the trial.			6
		c. Provide a modification timeline.			6
III.	Responsible Parties	State who planned, reviewed and approved the modifications.			6
IV.	Interim data	If modifications were informed by trial data, describe how the interim data were used, including whether they were examined by study group, and whether the individuals reviewing the data were blinded to the treatment allocation.			N/A
**CONSORT Number and Item**		For each row, if important modifications occurred check "direct impact" and/or "mitigating strategy" and describe the changes in the trial manuscript or supplement. Check "no change" for items that are unaffected in the extenuating circumstance.			**Page No.**
		**No Change**	**Impact***	**Mitigating Strategy****	
1	Title and abstract	X			
2	Introduction	X			
3	Methods: Trial Design		X	X	6
4	Methods: Participants	X			
5	Methods: Interventions	X			
6	Methods: Outcomes		X	X	6
7	Methods: Sample Size	X			
8-10	Methods: Randomisation	X			
11	Methods: Blinding	X			
12	Methods: Statistical methods	X			

13	Results: Participant flow	X			
14	Results: Recruitment	X			
15	Results: Baseline data	X			
16	Results: Numbers analysed	X			
17	Results: Outcomes and estimation		X	X	7,8
18	Results: Ancillary analyses	X			
19	Results: Harms	X			
20	Discussion: Limitations	X			
21	Discussion: Generalisability	X			
22	Other information: Registration	X			
23	Other information: Protocol	X			
24	Other information: Funding	X			

*Aspects of the trial that are directly affected or changed by the extenuating circumstance and are not under the control of investigators, sponsor or funder.
**Aspects of the trial that are modified by the study investigators, sponsor or funder to respond to the extenuating circumstance or manage the direct impacts on the trial.

The CONSERVE-CONSORT Checklist is licensed by the CONSERVE Group under the Creative Commons Attribution-NonCommercial-NoDerivs 4.0 International license.

CONSORT 2010 checklist of information to include when reporting a randomised trial*

Section/Topic	Item No	Checklist item	Reported on page No
**Title and abstract**			
	1a	Identification as a randomised trial in the title	1
	1b	Structured summary of trial design, methods, results, and conclusions (for specific guidance see CONSORT for abstracts)	3
**Introduction**			
Background and objectives	2a	Scientific background and explanation of rationale	5
	2b	Specific objectives or hypotheses	5
**Methods**			
Trial design	3a	Description of trial design (such as parallel, factorial) including allocation ratio	5
	3b	Important changes to methods after trial commencement (such as eligibility criteria), with reasons	6
Participants	4a	Eligibility criteria for participants	5
	4b	Settings and locations where the data were collected	5
Interventions	5	The interventions for each group with sufficient details to allow replication, including how and when they were actually administered	6
Outcomes	6a	Completely defined pre-specified primary and secondary outcome measures, including how and when they were assessed	6
	6b	Any changes to trial outcomes after the trial commenced, with reasons	6
Sample size	7a	How sample size was determined	6
	7b	When applicable, explanation of any interim analyses and stopping guidelines	NA
Randomisation:			
Sequence generation	8a	Method used to generate the random allocation sequence	6
	8b	Type of randomisation; details of any restriction (such as blocking and block size)	6
Allocation concealment mechanism	9	Mechanism used to implement the random allocation sequence (such as sequentially numbered containers), describing any steps taken to conceal the sequence until interventions were assigned	6
Implementation	10	Who generated the random allocation sequence, who enrolled participants, and who assigned participants to interventions	6

Blinding	11a	If done, who was blinded after assignment to interventions (for example, participants, care providers, those assessing outcomes) and how	6
	11b	If relevant, description of the similarity of interventions	NA
Statistical methods	12a	Statistical methods used to compare groups for primary and secondary outcomes	7
	12b	Methods for additional analyses, such as subgroup analyses and adjusted analyses	7
**Results**			
Participant flow (a diagram is strongly recommended)	13a	For each group, the numbers of participants who were randomly assigned, received intended treatment, and were analysed for the primary outcome	7
	13b	For each group, losses and exclusions after randomisation, together with reasons	7
Recruitment	14a	Dates defining the periods of recruitment and follow-up	7
	14b	Why the trial ended or was stopped	NA
Baseline data	15	A table showing baseline demographic and clinical characteristics for each group	13
Numbers analysed	16	For each group, number of participants (denominator) included in each analysis and whether the analysis was by original assigned groups	3, 7, 8, 13, 14, 15, 16
Outcomes and estimation	17a	For each primary and secondary outcome, results for each group, and the estimated effect size and its precision (such as 95% confidence interval)	3, 7, 8, 13, 14, 15, 16
	17b	For binary outcomes, presentation of both absolute and relative effect sizes is recommended	3, 7, 8, 13, 14, 15, 16
Ancillary analyses	18	Results of any other analyses performed, including subgroup analyses and adjusted analyses, distinguishing pre-specified from exploratory	3, 7, 8, 13, 14, 15, 16, Supplemental tables
Harms	19	All important harms or unintended effects in each group (for specific guidance see CONSORT for harms)	8
**Discussion**			
Limitations	20	Trial limitations, addressing sources of potential bias, imprecision, and, if relevant, multiplicity of analyses	9
Generalisability	21	Generalisability (external validity, applicability) of the trial findings	9
Interpretation	22	Interpretation consistent with results, balancing benefits and harms, and considering other relevant evidence	9
**Other information**			
Registration	23	Registration number and name of trial registry	3, 7
Protocol	24	Where the full trial protocol can be accessed, if available	5
Funding	25	Sources of funding and other support (such as supply of drugs), role of funders	3, 7

*We strongly recommend reading this statement in conjunction with the CONSORT 2010 Explanation and Elaboration for important clarifications on all the items. If relevant, we also recommend reading CONSORT extensions for cluster randomised trials, non-inferiority and equivalence trials, non-pharmacological treatments, herbal interventions, and pragmatic trials. Additional extensions are forthcoming: for those and for up to date references relevant to this checklist, see www.consort-statement.org.
